# Structures of the volume-regulated anion channel LRRC8A/D in activating and inhibiting conditions

**DOI:** 10.1038/s41467-026-77508-x

**Published:** 2026-09-17

**Authors:** Elena F. Lehmann, Fabienne Stierli, Sonja Rutz, Dawid Deneka, Raimund Dutzler

**Affiliations:** Department of Biochemistry University of Zurich, Winterthurerstrasse 190, Zurich, Switzerland

**Keywords:** Cryoelectron microscopy, X-ray crystallography, Permeation and transport, Chloride channels

## Abstract

Volume-regulated anion channels, which are activated in response to hypotonicity, are heteromers composed of LRRC8 family members. They consist of the obligatory subunit LRRC8A and at least another paralog that determines their substrate preference. Here we investigate heteromeric assemblies composed of LRRC8A and D subunits, the latter of which confer permeability to amino acids, osmolytes and anti-cancer drugs. Using patch-clamp electrophysiology, we show that LRRC8A/D channels are reversibly activated by cell swelling and that their response is modulated by sybodies targeting the A subunits, which either potentiate or repress channel activity. Structures of the LRRC8A/D channel in complex with the inhibitory sybody Sb1 define the channel stoichiometry of four A and two D subunits, with Sb1 stabilizing the A subunits in a similar closed channel conformation as found in homomeric LRRC8A assemblies. In LRRC8A/D heteromers, the two D subunits weaken the arrangement of A subunits and thus enhance the activation properties of the channel. This channel conformation is strongly perturbed upon binding of the potentiating sybody Sb4, which disrupts the packing of the cytoplasmic domains linking their mobility to pore opening.

## Abstract (web summary)

Members of the LRRC8 family form heteromeric ion channels that are activated upon cell swelling. Here authors describe the architecture of assemblies of LRRC8A and D subunits and their conformational changes during activation.

## Introduction

Volume-regulated anion channels (VRACs) are activated in response to the swelling of cells in a hypotonic environment^1–3^. As such, they participate in the regulatory volume decrease, a cellular mechanism that leads to the efflux of anions and osmolytes to equilibrate osmotic differences, allowing cells to return to their original volume^4–8^. Besides their role in cell volume regulation, VRACs were proposed to also be involved in other processes including signaling, insulin secretion, cell division and apoptosis^9–12^. These proteins form heteromers of the LRRC8 family, which contains five closely related paralogs in humans termed LRRC8A-E^13^. In a cellular environment, all channels consist of the obligatory LRRC8A (also termed SWELL1) and at least one other family member^13–15^. The subunit composition was found to affect substrate selectivity and activation properties^13,15–17^. Among the five LRRC8 paralogs, LRRC8D is of particular importance as its presence has been shown to increase the permeability of the channel for the neurotransmitters glutamate and GABA, the osmolytes taurine and myo-inositol, the antibiotic blasticidin S and platinum-based anti-cancer drugs^16,18–20^.

Despite the observed role of family members in shaping permeation properties, the exact composition of VRAC channels in a physiological context is still unknown. Although the unconstrained arrangement of subunits could in principle lead to the formation of nearly two thousand unique hexameric assemblies, there appears to be a much lower number of distinct channels formed, whose organizations are dictated by the biophysical properties of the individual building blocks^21,22^. The features of individual subunits were defined in homomeric structures of LRRC8A^23–27^, LRRC8C^21,28^ and LRRC8D^29^, which all oligomerize when expressed on their own. In the case of LRRC8A, different structures have defined the general architecture of the family^23^. LRRC8A assembles as hexameric complex of tightly interacting modular subunits, consisting of a membrane-inserted pore domain (PD) exhibiting six-fold rotational symmetry and cytoplasmic leucine rich repeat domains (LRRDs), which have rearranged to maximize the interaction surface between subunit pairs^23^. These pairs of tightly interacting LRRC8A subunits constitute the modules of a three-fold symmetric hexameric assembly, which functions as anion-selective channel with low open probability and compromised activation properties^15,23,30,31^. Despite the generally similar organization of C and D subunits^32^, their assemblies show structural properties that differ from LRRC8A. In case of LRRC8C, homomers assemble as larger, loosely-packed heptamers with weaker subunit interactions^21,28^. In case of LRRC8D, the hexameric subunits adopt a distorted, two-fold symmetric organization with presumably loose LRRD interactions^29^. The different interaction properties within homomeric assemblies were shown to influence the subunit organization of LRRC8 heteromers. In studies of channels consisting of LRRC8A and C, the A subunits dominate in hexameric arrangements with either 2:1 or 5:1 stoichiometric ratio^21,22^. In channels showing an A:C ratio of 2:1, two tightly interacting LRRC8A pairs enframe a pair of LRRC8C subunits destabilizing the tight interactions of the former^21^. However, it is currently unclear to what extent the observed arrangement also extends to channels with different subunit composition.

Besides their heteromeric organization, the mechanisms underlying channel activation remain poorly understood^33,34^. While the characteristics of open and closed pore conformations have remained elusive, there is increasing evidence for the association of activation with a progressive release of interactions between LRRDs^35–37^. The consequent increase of their mobility has led to their assignment as regulatory modules sensing cellular signals and transmitting them to the PD to trigger the opening of a gate whose identity is still controversial^22,27,38,39^.

In our current study, we were interested in the organization of heteromeric VRACs composed of LRRC8A and D subunits and in the features underlying their activation. To obtain insight into both properties, we engaged in structural and functional investigations of LRRC8A/D channels and their response to specific modulators binding to the cytoplasmic LRRDs^38^. Our study defines the subunit organization of heteromers as an ordered arrangement of four consecutive A and two interspersed D subunits. These adopt a perturbed conformation upon binding of a synthetic nanobody, which acts as a potentiator of channel activation.

## Results

### Functional characterization of LRRC8A/D channels

**Fig. 1:**
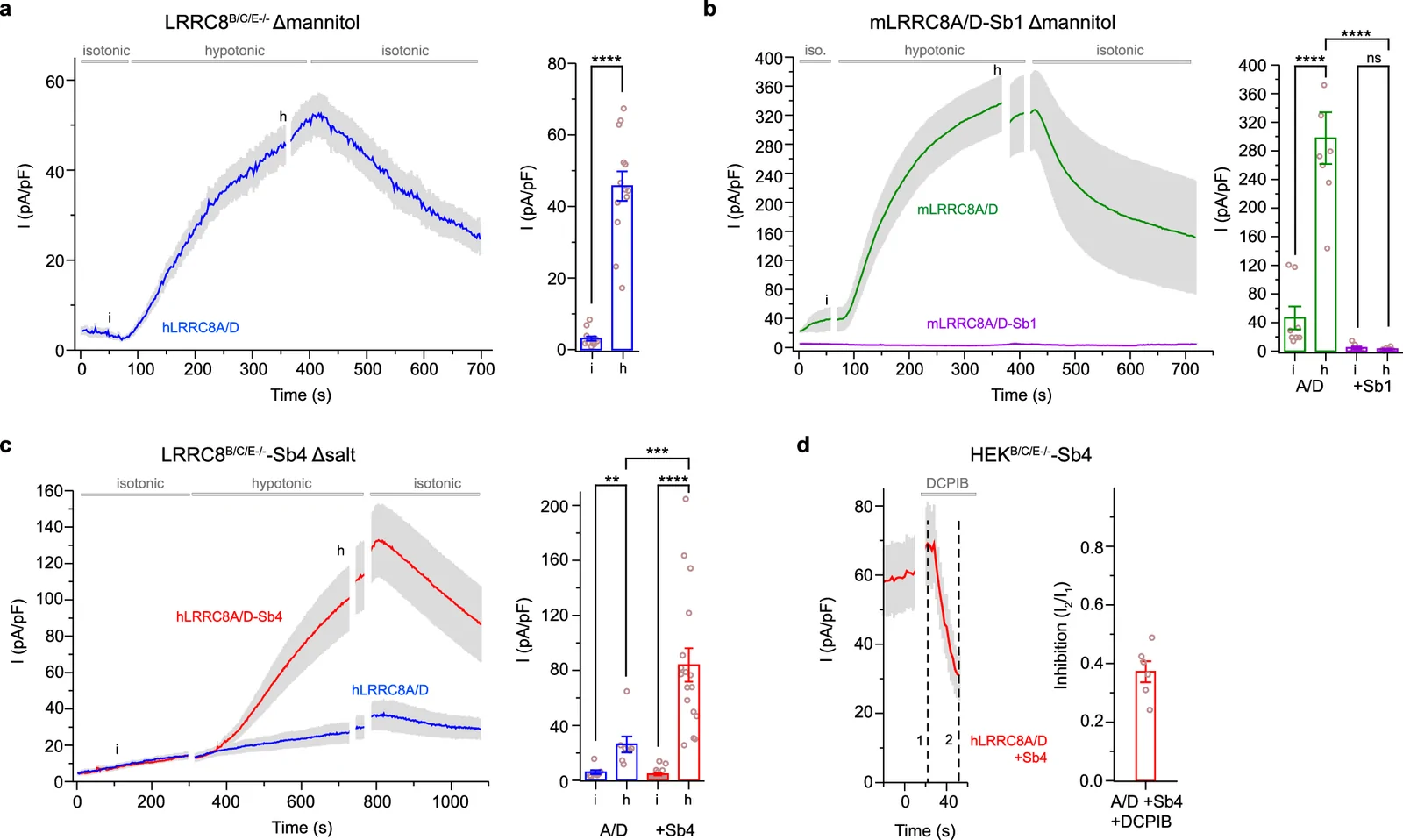
Functional properties of LRRC8A/D channels. **a**–**c** VRAC current response to the osmotic swelling of cells. Shown is the mean current density (left) and individual measurements at indicated time points (right). **a** LRRC8^B,C,E-/-^ cells endogenously expressing A and D subunits recorded in Δmanitol buffers (*n* = 13, *p* < 0.0001). **b** LRRC8^-/-^ cells transfected with mouse LRRC8A and D constructs (green, *n* = 8, *p* < 0.0001) and additionally Sb1 (indigo, *n* = 7, *p* = 0.413) recorded in Δmanitol buffers. The difference between activating responses in presence and absence of Sb1 is statistically significant (*p* < 0.0001). Traces, left, show the mean of 6 (AD) and 7 (AD+Sb1) representative recordings. **c** LRRC8^B,C,E-/-^ cells recorded in absence (*n* = 8, *p* = 0.097) and presence of Sb4 added to the pipette solution (*n* = 17, *p* < 0.0001) in Δsalt buffers. The difference between activating responses in presence and absence of Sb4 is statistically significant (*p* = 0.0003). Traces, left, show the mean of 8 (AD) and 14 (AD+Sb4) representative recordings. **a**–**c** Differences were evaluated by two-tailed unpaired *t* testing with Welch’s correction applied. **d** Inhibition of Sb4-potentiated currents of LRRC8^B,C,E-/-^ cells after exposure to hypotonic conditions (as displayed in Supplementary Fig. 1i) by application of 50 µM DCPIB in the bath solution (*n* = 6). Bars (right) show relative current decline at indicated time points. **a**–**d** Current densities show values at 100 mV recorded with a ramp protocol (Supplementary Fig. 1a). The perfusion with buffers of distinct composition is indicated. Errors are s.e.m. i and h refer to time points for current values displayed in bar graphs.

At the start of our investigation, we aimed to identify conditions that would allow us to stabilize conducting and non-conducting states of LRRC8A/D channels and thus proceeded with their functional characterization. Our studies have relied on a HEK293 cell-line carrying genetic knockouts of LRRC8B, C and E (LRRC8^B,C,E-/-^), which solely expresses LRRC8A and LRRC8D subunits, and another cell-line with complete knockout of all five LRRC8 genes (LRRC8^-/-^) for the heterologous overexpression of human and mouse LRRC8A and D constructs^13^. Both cell lines were used in patch-clamp electrophysiology experiments in the whole-cell configuration, where chloride currents in  response to changes in the osmolarity of the surrounding media were recorded. For our measurements, we have relied on two different buffer compositions termed Δsalt and Δmannitol, with the latter imposing a larger osmotic gradient, leading to enhanced activating effects. The evoked currents were recorded by a consecutive series of ramp protocols that allowed the contiguous monitoring of current-voltage relationships during the activation process (Supplementary Fig. 1a and Supplementary Table 1). When measured in Δmannitol conditions, LRRC8^B,C,E-/-^ cells did not show detectable conduction in isotonic buffer, while they exhibited a substantial increase of currents that developed over a few minutes upon perfusion with hypotonic solutions (Fig. 1a). In parallel, we also probed the response after transfection of human and murine constructs in LRRC8^-/-^ cells in the weaker activating Δsalt conditions. While we found a moderate increase of currents for human LRRC8A/D channels, these were pronounced for their mouse orthologs (Supplementary Fig. 1b). The smaller response of human channels appears to be a consequence of both, their lower surface expression and reduced sensitivity (Supplementary Fig. 1c).

After revisiting the conduction properties of LRRC8A/D channels, we have investigated the effect of specific nanobody-based binders (termed sybodies)^40^ on their activity. The sybodies specifically target the LRRD of the LRRC8A subunit and were previously described to differently modulate VRAC activity in relation to their epitope, with Sb1 diminishing and Sb4 increasing the current response^38^. For these investigations we have applied suitable conditions to best illustrate the effect of the respective binders (Supplementary Table 1).

Due to their higher elicited currents, we have carried out inhibition experiments with the overexpressed murine constructs in Δmannitol conditions, which showed large hypotonicity- evoked currents for cells transfected with LRRC8A and D constructs (Fig. 1b). For comparison, we have investigated cells that were co-transfected with both LRRC8 subunits and additionally the YFP-tagged sybody Sb1 to identify transfected cells. For such cells, we observed a cease of VRAC activity (Fig. 1b), in line with the described role of Sb1 as allosteric inhibitor preventing channel activation^38^.

In our experiments with sybody Sb4, which was previously shown to enhance VRAC activity^38^, endogenous currents were recorded in different HEK293 cell-lines in Δsalt conditions, where the response upon change to hypotonic conditions was weak (Fig. 1c, and Supplementary Fig. 1d). Since the overexpression of Sb4, as carried out for Sb1, affected the cellular fitness, the binder was applied via the pipette solution at a concentration of 1 µM and was allowed to equilibrate with the cytoplasm after establishment of the whole-cell configuration prior to the  application of hypotonic buffer. In such a setup, the addition of Sb4 has strongly potentiated VRAC currents in HEK293 cells and LRRC8A/D currents in the LRRC8^B,C,E-/-^ cell line (Fig. 1c and Supplementary Fig. 1d). In the latter, the application via the patch pipette has led to an unchanged response under isotonic conditions and a pronounced, about 18-fold increase of the current density after the application of hypotonic solution. These currents are more than three times higher than observed in recordings in the absence of the sybody under equivalent conditions (Fig. 1c). In the presence and absence of Sb4, the response carries the characteristics of channels composed of LRRC8A and D subunits^13,16^, which are distinguished by a strong outward-rectification and slight inactivation at positive voltage, properties that generally resemble the VRAC current features observed in HEK293 cells (Supplementary Fig. 1e–h). The LRRC8A/D currents obtained in presence of Sb4 declined upon addition of the VRAC inhibitor DCPIB^41^, illustrating that even in complex with Sb4, the channels have retained their general pharmacological properties (Fig. 1d and Supplementary Fig. 1i). The absent effect at isotonic and large response after hypotonic perfusion underlines the role of Sb4 as potentiator that acts synergistically with cell swelling to increase the open probability of LRRC8A/D channels.

### Characterization of homomeric LRRC8D

**Fig. 2:**
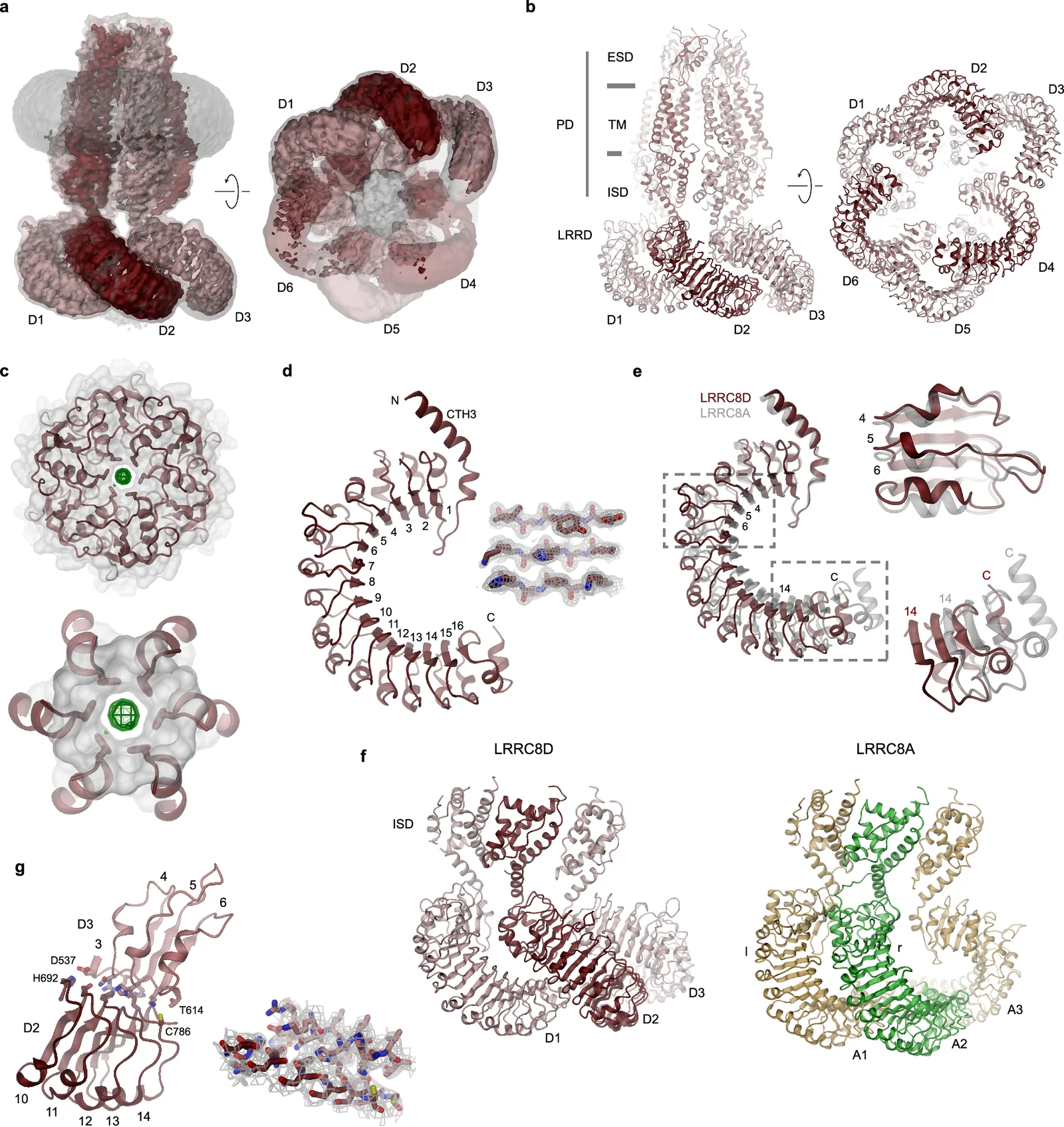
Structure of the LRRC8D homomer. **a** Cryo-EM density of a dataset of the LRRC8D homomer at 3.0 Å (contoured at 10σ) in indicated orientations. A blurred map at lower contour (b=100, 5σ) is superimposed to also show the envelope of regions with weaker density. **b** Ribbon representation of the LRRC8D channel with structural regions and membrane boundaries indicated. **c** View of the ESD forming the selectivity filter from the extracellular side (top). A blow-up of the pore is shown below. The molecular surface is superimposed on a ribbon of the protein. Green mesh shows residual density (contoured at 10σ) found in the pore. **d** Ribbon representation of the X-ray structure of the isolated LRRD of LRRC8D. Inset shows 2Fo-Fc density of a section of the map (at 1.9 Å, contoured at 1σ) superimposed on the refined model. **e** Superposition of the LRRDs of LRRC8A and D on the first three leucine rich repeats. Insets show blow-up of indicated regions. **f** Comparison of the ISD and the LRRD regions of three interacting subunits of LRRC8D (left) and LRRC8A (PDBID: 7P5V). l, and r correspond to the left and right position of a tightly interacting LRRC8A domain pair viewed from the outside of the channel. **g** Interaction region between LRRDs of LRRC8D in D2 and D3 positions. Selected sidechains are shown as sticks. Inset shows density (contoured at 10σ) superimposed on the refined model (right).

After gaining insight into the functional properties of LRRC8A/D channels and their modulation by sybodies targeting the LRRDs of A subunits, we engaged in structural studies of heteromeric assemblies consisting of murine LRRC8A and D orthologs (Supplementary Fig. 2). Initially, we aimed to improve our comprehension of the constituents of the complex. Whereas high resolution structures for both parts of the LRRC8A subunits were available from previous work^38^, a structure of human LRRC8D has provided a view of the homomeric protein at moderate resolution (4.25 Å) with poorly resolved LRRDs^29^. While such homomeric LRRC8D channels do properly assemble, they would not localize at the plasma membrane and are thus presumably not relevant in a cellular setting^13^. To better define this subunit at high resolution, we have purified the full-length homomeric assembly of mouse LRRC8D overexpressed in LRRC8^-/-^ cells and collected data by cryo-EM that permitted the reconstruction of a map at 3.0 Å (Supplementary Fig. 3 and Table 1). This map displays a hexamer with well-resolved PDs and strong density of the LRRDs in three consecutive subunits, whereas the envelope of the weaker defined domains becomes visible only at lower map contour (Fig. 2a, b).

**Table 1:** Cryo-EM data and refinement statistics

	LRRC8A/D/Sb1^c1^ 9T5M	LRRC8A/D/Sb1^c2^ 9T5N	LRRC8A/D/Sb4 9T5O	LRRC8D 9T5P
**Data collection and processing**				
Magnification	130,000	130,000	190,000	130,000
Voltage (kV)	300	300	200	300
Electron exposure (e^-^/Å^2^)	63.03, 59.64	63.03, 59.64	60.65, 61.39	59.28
Defocus range (μm)	−2.4 to −1.0	−2.4 to −1.0	−2.6 to −0.8	−2.6 to −0.8
Pixel size (Å)	0.651	0.651	0.72	0.651
Symmetry imposed	C1	C1	C1	C1
Final particle images (no.)	387,972	266,318	111,259	530,537
Map resolution (Å)	3.02	3.16	3.97	2.99
FSC threshold	0.143	0.143	0.143	0.143
Map resolution range (Å)	1.99–11.2	2.03–10.9	1.88–11.8	2.48–12.1
**Refinement**				
Initial model used (PDB code)	7P5V	7P5V	-	7P5V
Model resolution (Å)	3.4	6.8	4.3	3.3
FSC threshold	0.5	0.5	0.5	0.5
Map sharpening *B* factor (Å^2^)	−50	−52	−35	−60
Model composition				
Non-hydrogen atoms	38’976	31’806	19’782	34’368
Protein residues	4760	3865	2397	4212
Ligands	0	0	2	0
*B* factors (Å^2^)				
Protein	295.44	122.06	171.62	225.52
Ligand			189.92	
R.m.s. deviations				
Bond lengths (Å)	0.004	0.002	0.004	0.002
Bond angles (°)	0.479	0.534	0.570	0.455
Validation				
MolProbitz score	2.21	2.33	3.29	2.20
Clashscore	8.65	9.80	65.68	8.97
Poor rotamers (%)	4.24	6.54	6.83	4.07
Ramachandran plot				
Favored (%)	96.03	96.83	94.73	96.24
Allowed (%)	3.92	3.14	5.22	3.62
Disallowed (%)	0.04	0.03	0.04	0.14

In contrast to the previously determined human LRRC8D structure^29^, which displayed a two-fold relationship of subunit triplets, the mouse LRRC8D does not adopt the same arrangement (as illustrated in the RMSD of 4.2 Å for the superposition of the hexameric PDs, Supplementary Fig. 4a). Instead, it shows a slightly asymmetric assembly that generally resembles the six-fold symmetric organization of LRRC8A homomers^23^ (with PDs superimposing with an average RMSD of 1.7 Å for the hexamer, while values of subunits range between 1.2 and 2.1 Å, Supplementary Fig. 4b).

The extracellular subdomain (ESD) carrying the selectivity filter is tightly packed and symmetric (Fig. 2c and Supplementary Fig. 4c). A unique feature in this region concerns the residue at its constriction, where an arginine in LRRC8A is replaced by a phenylalanine in LRRC8D (Phe 143), which is less extended and adopts fewer possible side-chain rotamer conformations. In this filter, the rigid sidechains of Phe 143 are oriented edge-to-edge to create an aromatic cage that leads to an expanded pore of hydrophobic character with a diameter of 6.6 Å (Fig. 2c). The center of this filter contains an elongated density of unknown origin that could correspond to a component of the buffer solution, a bound detergent or a fatty acid chain (Fig. 2c, Supplementary Fig. 4c). Another difference compared to the A subunits concerns the loop connecting TM1 and 2, which although longer in LRRC8D, is less structured, leading to a lack of density between residues 62 and 133 (Supplementary Fig. 4d). Besides the tightly packed ESD, we also find inter-subunit contacts in the intracellular subdomains (ISDs) and in the transmembrane region (TM, Supplementary Fig. 4e). Unlike in the previously determined structure of human LRRC8D which showed weak density of the N-terminus^29^, the first 14 residues are not defined in mouse LRRC8D, resembling the case of most available LRRC8A structures^23–26^.

In the described map, the LRRDs are less well resolved than the PD, reflecting their higher mobility (Fig. 2a). While three consecutive domains show strong density at a resolution that permits the recognition of structural features, the map is not sufficiently detailed to confidently model their entire structure (Fig. 2a and Supplementary Fig. 4f). We have thus crystallized a construct of the isolated LRRD and determined its structure by X-ray crystallography at 1.96 Å (Fig. 2d and Table 2). These coordinates were subsequently placed into the respective regions of the full-length LRRC8D map and used for the refinement of a model consisting of both parts of the protein to obtain a detailed view of the entire assembly (Fig. 2b). In the full-length protein, all LRRDs show a similar strong tilt by 50° towards the membrane plane but a variable inclination with respect to the pore axis that differs by up to 15° between subunits (Supplementary Fig. 4g).

**Table 2:** X-Ray data and refinement statistics

	LRRC8D LRRD 9T5R	LRRC8A LRRD/Sb4 9T5Q
**Data collection**		
Space group	P212121	P1
Cell dimensions		
a, b, c (Å)	36.0, 89.5, 143.7	55.95, 73.4, 81.92
ɑ, β, γ (°)	90.0, 90.0, 90.0	111.04, 94.35, 110.31
Wavelength (Å)	1	1
Resolution (Å)	44.8 – 1.96	40.08 – 1.6
R_merge_	9.8 (197.7)	10.9 (91.9)
*I*/ σ*I*	22.43 (1.63)	11.74 (2.33)
Completeness (%)	96.6 (99.6)	96.4 (95.2)
Redundancy	26.1 (24.9)	7.2 (7.1)
**Refinement**		
Resolution (Å)	1.96	1.6
No. reflections	34115	142196
R_work_/R_free_	19.37/22.64	19.84/22.89
No. atoms		
Protein	3317	8423
Ligands	0	0
Solvent	326	838
*B*-factors		
Protein	45.04	22.3
Ligands	-	-
Solvent	48.13	29.63
Root-mean-square deviations		
Bond lengths (Å)	0.007	0.006
Bond angles (°)	0.86	0.84
**Validation**		
Clashscore	2.36	3.45
Poor rotamers (%)	0	0.75
Ramachandran plot		
Favored (%)	95.25	96.62
Allowed (%)	4.75	3.38
Disallowed(%)	0	0

In its overall organization, the LRRD of the D subunit closely resembles the equivalent unit of LRRC8A (Fig. 2d, e). Both share the same length, consist of the same number of leucine-rich repeats (i.e., 16 including the deteriorated capping repeat on the C-terminus) and superimpose with a root mean square deviation (RMSD) of 1.6 Å. In this superposition, the largest structural differences between the two protein components are found at the N-terminal part of the domain, which determines the structural relation to the PD. Consequently, the general differences between both domains are better appreciated upon the alignment of their first three repeats (Fig. 2e). The somewhat smaller curvature of the horseshoe-like fold of LRRC8D, which can be attributed to unique features in repeats 4–6, causes an increased separation of repeats culminating in a distance between both C-termini of 8 Å (Fig. 2e). The described structural differences concur with the moderate sequence conservation, with both domains sharing 60% sequence identity (Supplementary Fig. 2). The consequent divergence in their surface properties is manifested in the pronounced selectivity of sybodies targeting the A subunit^38^ as consequence of the altered interaction interface (Supplementary Figs. 2 and 4h, i). Together with features of the preceding PDs, the unique properties of the LRRDs also determine the distinct inter-subunit interactions observed in the structures of LRRC8A^23^ and D homomers (Fig. 2f). These interactions were found to be most extended in the tight interface of LRRC8A, where the LRRDs interact along the entire domain (Supplementary Fig. 4j, k), whereas the interactions between LRRDs of the D-subunit are confined to repeats 10-14 in one domain and 3-6 for its interacting domain at the +1 position. (Fig. 2g).

In summary, despite the generally conserved architecture, the unique features of LRRC8D and its distinct assembly properties are expected to affect the conformational preferences of the subunit in a heteromeric complex and its interactions with still unknown cellular partners.

### LRRC8A/D/Sb1 structure

**Fig. 3:**
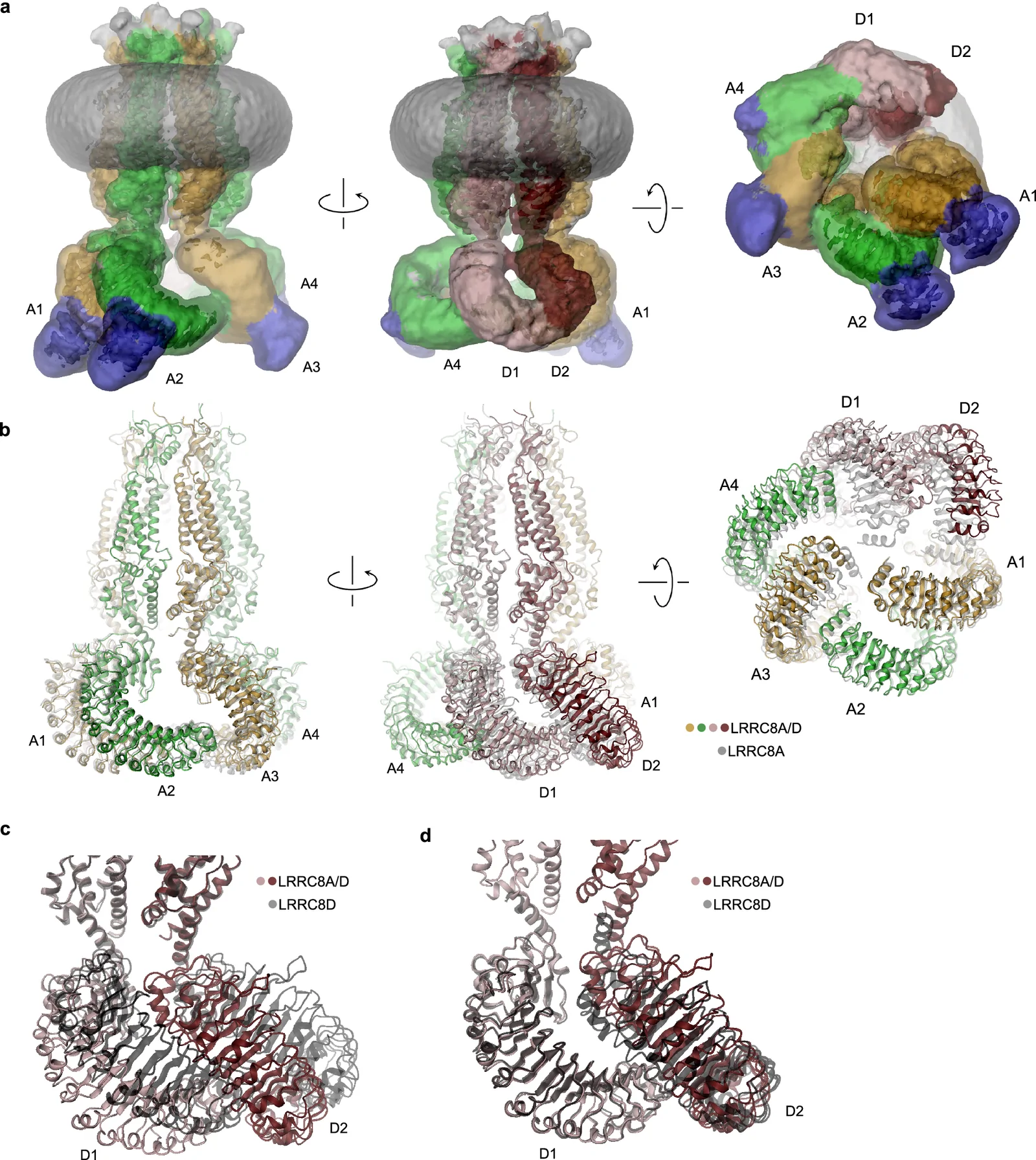
Structure of the LRRC8A/D/Sb1 complex. **a** Cryo-EM density of conformation 1 (c1) from a dataset of the heteromeric LRRC8A/D/Sb1 complex at 3.0 Å (contoured at 10σ) in indicated orientations. A blurred map at lower contour (b=100, 5σ) is superimposed to also show the envelope of regions with weaker density. Density corresponding to bound sybodies is indicated in blue. **b** Ribbon representation of the LRRC8A/D/Sb1^c1^ complex with subunit positions labeled. LRRC8A subunits are colored in orange and green and LRRC8D subunits in pink and red, respectively. The superimposed structure of LRRC8A (PDBID 7P5V) is shown in gray for comparison. **c** View of the ISD and LRRD of two subunits (D2 and D3 position) of the LRRC8D homomer superimposed on the PD of the D1 and D2 subunits of the LRRC8A/D/Sb1^c1^ complex shows a larger tilt of the LRRDs of the homomeric channel towards the membrane. **d** The superposition of the isolated LRRD domain pair of the D2 and D3 subunits of the LRRC8D homomer demonstrates the preservation of the relative orientation between both domains.

With detailed knowledge of the structural features of its constituents, we continued with investigations of LRRC8A/D channels that were overexpressed by co-transfection of LRRC8^-/-^ cells with appropriate constructs and purified via an SBP tag attached to the less abundant LRRC8D subunit (Supplementary Fig. 5a), whose yield was about half of that of homomeric LRRC8A channels if expressed on its own. Based on this observation and the monitoring of the fluorescence ratio of the differentially tagged LRRC8 subunits during purification, we expect a ratio of D:A subunits of 1.75:1 in this preparation procedure. The concentrated LRRC8A/D sample was supplemented with a 1.5 molar excess of the inhibitory Sb1 to label LRRC8A subunits, vitrified for cryo-EM imaging and used for the collection of a large dataset (Supplementary Fig 5b). In this dataset, the presence of Sb1 allowed the unambiguous assignment of A subunits, and it stabilized a non-conducting state of the protein. During data processing, particles of heteromeric LRRC8A/D containing bound Sb1 were readily identified during 2D classification and 3D reconstruction, allowing their separation from particles not containing Sb1 density (Supplementary Fig 5b). The latter presumably represent LRRC8D homomers that constitute about 29% of all particles. From particles of the LRRC8A/D/Sb1 complex, we obtained two reconstructions at 3.0 and 3.2 Å resolution termed LRRC8A/D/Sb1^c1^ and LRRC8A/D/Sb1^c2^, respectively (Supplementary Fig. 5, Table 1). Both structures share a generally similar organization of two consecutive pairs of LRRC8A subunits that are distinguished by bound Sb1 (termed A1-A4, assigned in a clockwise manner when viewed from the intracellular side) and an interspersed pair of subunits with more disordered LRRDs that were assigned as D subunits (i.e., D1 and D2, Fig. 3a, b, Supplementary Fig. 6a, b). The mutual equivalence of the PD in both structures is manifested in the low RMSD of 0.7 Å, whereas the increased RMSD of the full-length protein of 3.0 Å reflects larger conformational differences in the LRRD region. The RMSD of either PD to the equivalent unit of the LRRC8A/Sb1 complex of only 0.8 Å illustrates that the presence of D subunits did not perturb the general 6-fold symmetric organization of the membrane-inserted region of the channel (Fig. 3b and Supplementary Fig. 6b). In both structures, the pair-wise organization of tightly interacting LRRC8A subunits resembles the organization in the LRRC8A/Sb1 structure^38^. In the two reconstructions, alternate pairs of A subunits show stronger density of their LRRDs (i.e., A1 and A2 in LRRC8A/D/Sb1^c1^ and A3 and A4 in LRRC8A/D/Sb1^c2^), whereas the other pair becomes visible at lower map contour, reflecting the effect of neighboring D subunits on the LRRC8A arrangement (Fig. 3a, and Supplementary Fig. 6a). Contrary to the A subunits, the LRRDs of the two D subunits are less well defined and adopt distinct conformations (Fig. 3a, b and Supplementary Fig. 6a). A generally similar subunit arrangement was independently observed in a recent study on the LRRC8A/D complex that used genetically modified A constructs to facilitate their identification^42^.

In LRRC8A/D/Sb1^c1^, the conformations of the strongly defined subunits A1 and A2 are essentially as found in the LRRC8A/Sb1 homomer^38^. In contrast, the conformations in position A3 and A4, have changed moderately to respond to the altered environment of the neighboring D subunits (Fig. 3b). For both D subunits, weak density in the original and a blurred map confine conformations of their LRRDs that are both less inclined compared to their orientation in the homomeric LRRC8D structure but which preserved their mutual interactions (Fig. 3a, c, d). The observed rearrangement is required to accommodate the domains in the constraint space delineated by the neighboring A subunits (Fig. 3b). The observed conformations of the LRRDs of the D subunits do not resemble the arrangement in the tight interface relating A subunits, illustrating that the distinct preferred interactions established by these paralogs are also maintained in a heteromeric assembly, despite the similar arrangement of their PDs (Figs. 2f, 3b).

With respect to its LRRD conformations, the difference between LRRC8A/D/Sb1^c2^ and LRRC8A/Sb1 is larger, where even the better-defined subunit pair encompassing A3 and A4 has changed its conformation by moving towards the neighboring D subunits (Supplementary Fig. 6a, b). A1 and A2 show a stronger rearrangement with the C-terminus of A2 being poorly defined in the density (Supplementary Fig. 6a). Some residual density of the LRRD of D subunits is also observed in this structure, although both domains are not sufficiently well resolved to permit their placement with confidence (Supplementary Fig. 6a).

In line with the observed inhibitory function of Sb1 (Fig. 1b), we expect the observed LRRC8A/D/Sb1 structures to represent non-conducting, closed conformations of the protein.

### Structural features of the pore region

**Fig. 4:**
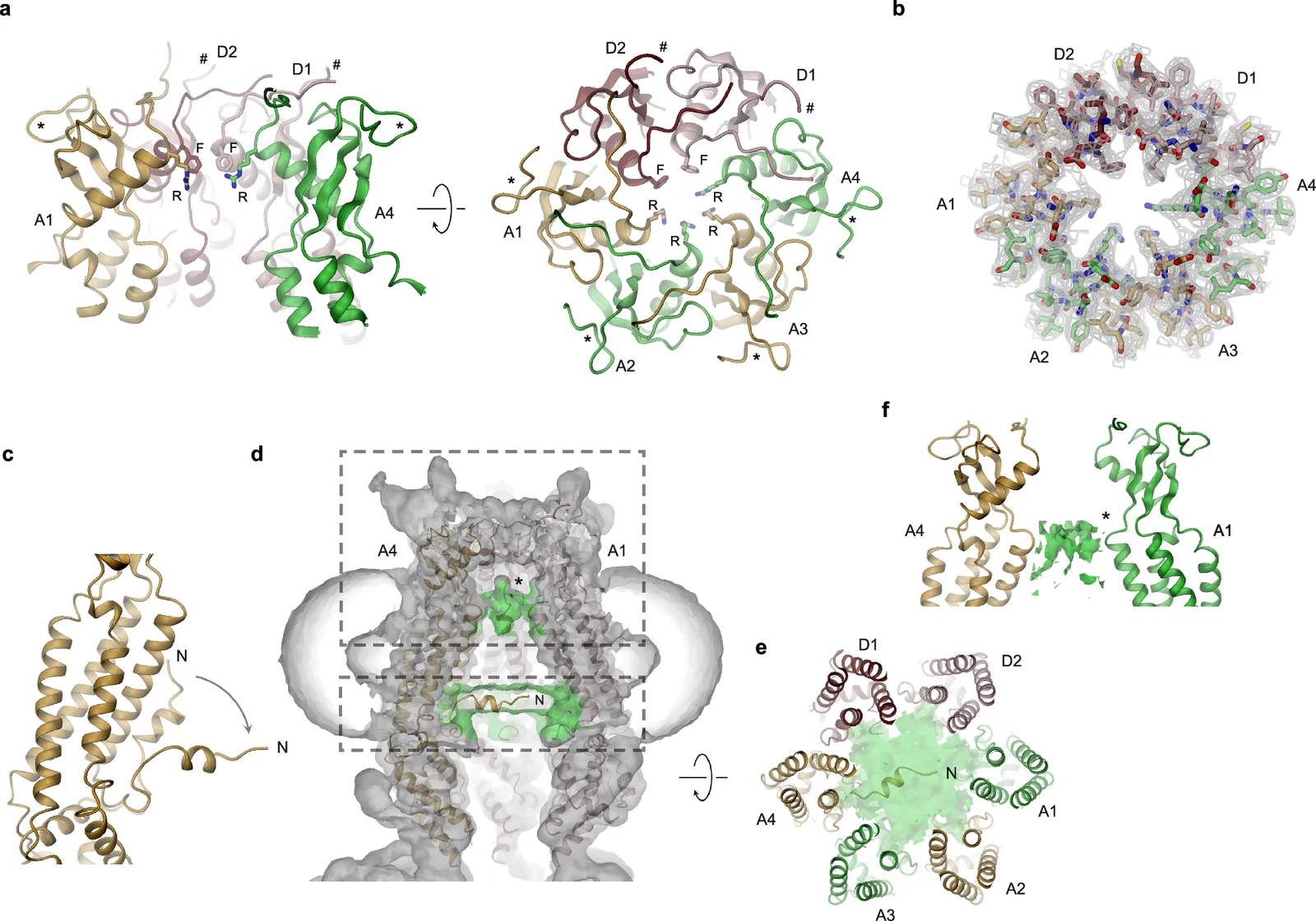
Pore domain features of the LRRC8A/D/Sb1^c1^ complex. **a** Ribbon representation of the ESD viewed parallel to the membrane with two front subunits removed (left) and from the extracellular side (right). Side chains of residues lining the pore constriction are shown as sticks. * Indicates loop region between TM1 and 2 in A subunits, # the same region in D subunits. **b** Extracellular view of the selectivity filter of the LRRC8A/D/Sb1^c1^ complex with cryo-EM density (contoured at 10σ) superimposed on the refined structure. **c** TM of a single subunit of an LRRC8A structure with defined N-terminal region (PDBID: 7XZH), and a modeled potential reorientation in the LRRC8A/D/Sb1^c1^ complex. **d** Blurred map (b=100) of the TM of the LRRC8A/D/Sb1^c1^ complex at low contour (5σ) with residual density in the pore region highlighted in green superimposed on the refined structure with front subunit containing the modeled rearranged N-terminus (shown in **c**) viewed from within the membrane (**e**). TM region of the same structure as in (**d**) viewed from the extracellular side with residual density in the pore region around the N-terminus shown in green. **f** View of residual density below the ESD in the unblurred map (contoured at 5σ) with two opposite subunits displayed.

Besides the unique arrangement of their LRRDs, the LRRC8A/D/Sb1^c1^ structure also defines the PD at sufficiently high resolution to permit an independent assignment of subunits, despite their overall similarity. One characteristic feature distinguishing A and D subunits relates to differences in the conformation of the loop region connecting TM1 and 2. Observed loop density in LRRC8A/D/Sb1^c1^ supports the described arrangement of two LRRC8A pairs framing a pair of LRRC8D subunits as allocated based on the density of the bound sybody (Fig. 3a and Supplementary Fig. 6a, c). This structure has provided a detailed view of the ESD harboring the most constricted part of the pore acting as selectivity filter. In the filter region, the two Phe 143 residues of the D subunits change the chemical nature and expand the cross-section of the filter, which is otherwise defined by the long and flexible Arg sidechains located at equivalent positions on the A subunits. Presumably these features alter the properties of the filter to expand the substrate selectivity towards amino acids and osmolytes as described in previous studies^16,18^ (Fig. 4a, b and Supplementary Fig. 6d).

In contrast to the well-defined density of the PD, except for few flexible loops on the cytoplasmic and extracellular sides, we have in no case observed density for the first 14 residues of A and D subunits, where mutations in LRRC8 proteins were shown to exert a strong impact on conductance properties and channel activity^39^ (Fig. 4c, d). While this region is disordered in most available structures, it was resolved in two cases of A^27^ and D^29^ homomers to line the channel wall and extend towards the extracellular side (Fig. 4c). In both structures of the LRRC8A/D/Sb1 complex, we find diffuse density in the region preceding Pro 15, the first defined residue in the structure, that could be attributed to the disordered N-terminus (Fig. 4d, e). Similarly, we find residual density at the boundary between TMD and ESD that was previously assigned to lipids occupying the pore region^22^, although this density is not sufficiently well defined to justify such assignment in our structure (Fig. 4d, f). Thus, whereas the LRRC8A/D/Sb1^c1^ structure does not reveal obvious features that would allow the assignment of a gate that controls ion permeation, it has defined the altered geometry of the ESD and thus provides initial insight into the features underlying the unique permeation properties of channels containing D subunits.

### LRRC8A/D/Sb4 structure

**Fig. 5:**
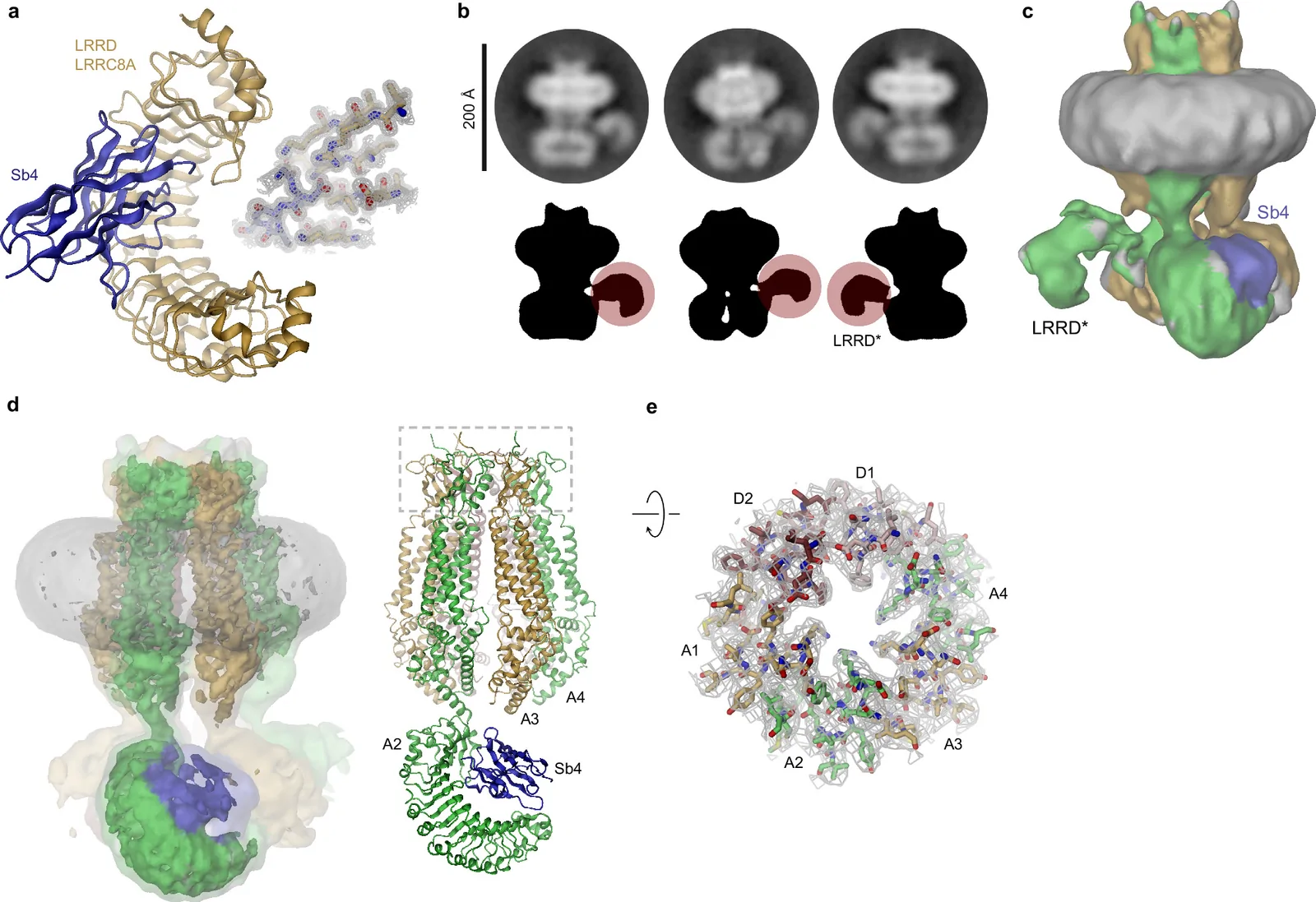
Structure of the LRRC8A/D/Sb4 complex. **a** X-ray Structure the LRRD of LRRC8A in complex with Sb4. Ribbon representation of the isolated LRRD of LRRC8A in complex with Sb4. Inset shows 2Fo-Fc density (at 1.6 Å, contoured at 1σ) of a section of the interaction interface superimposed on the refined model. **b** Selected 2D classes obtained from data of the LRRC8A/D/Sb4 complex depicting a strong reorientation of an LRRD towards the membrane (top) with schematic representation of the molecular envelopes and indicated domains (red) shown below. **c** Initial 3D class showing a similar strong movement of one of the LRRDs as observed in 2D classes. **d** Left, cryo-EM density of the refined map of the LRRC8A/D/Sb4 complex at 4.0 Å (contoured at 10σ). The PDs of all six subunits and an LRRD domain of a single subunit is defined at high map contour. A blurred map at lower contour (b = 100, 5σ) is superimposed to also display the envelope of regions with weaker density. Density corresponding to the bound sybody is indicated in blue. Right, ribbon representation of the refined LRRC8A/D/Sb4 complex. **e** Selectivity filter region of the ESD viewed from the extracellular side with cryo-EM density (contoured at 10σ) superimposed.

After characterizing LRRC8A/D channels in complex with Sb1, we became interested in the structural effects upon binding of the potentiating sybody Sb4 to gain deeper insight into channel activation. When added at a sub-stoichiometric ratio, Sb4 was previously found to occupy alternating subunits in LRRC8A homomers by targeting an epitope located at the concave surface of the LRRD that is exposed in the loose interface whereas it is buried between tightly interacting LRRC8A pairs^38^. As a consequence of the resulting increased flexibility of the LRRDs, the sybody was poorly resolved in the cryo-EM density of the LRRC8A/Sb4 structure, which has prohibited a detailed molecular interpretation of the interaction^38^. We have thus determined the X-ray structure of the LRRD of the A subunit in complex with Sb4 at 1.6 Å resolution (Fig. 5a, Table 2). This structure defines the tight interaction of the sybody with residues that are not conserved between A and D subunits, explaining its high subunit specificity^38^ (Supplementary Figs. 2 and 7). Sb4 contacts residues located on repeats 1–12 of the domain, burying 1756 Å^2^ of the combined molecular surface. The interactions are dominated by salt bridges and H-bonds involving charged residues but they also include few hydrophobic contacts (Supplementary Fig. 7b). Several of these charged residues are altered in LRRC8D, which together with the described altered curvature of its LRRD presumably accounts for the strongly decreased affinity to Sb4^38^ (Fig. 2e, Supplementary Figs. 2 and 7c, d).

After defining the interaction of Sb4 with the A subunits, we continued with the structural characterization of the LRRC8A/D/Sb4 complex. The preparation of this sample has followed the same protocol as used for the equivalent complex with Sb1, except for the lower salt concentration increasing VRAC activity and the higher concentration of the sybody added to the sample to promote its binding to all four LRRC8A subunits, including epitopes which under resting conditions were buried in the dimer interface^38^. A dataset of LRRC8A/D determined in the presence of Sb4 has revealed the structural consequences induced by the sybody, which are pronounced in the region of the LRRDs (Supplementary Fig. 8, Table 1). These are readily apparent in 2D reconstructions showing large changes of their conformation with single domains retracting from the pore axis and moving towards the membrane (Fig. 5b, Supplementary Fig. 8a). A similar displacement was found in classes obtained from an initial 3D reconstruction (Fig. 5c and Supplementary Fig. 8a), illustrating the strong interference of Sb4 with the LRRD organization, as expected upon disruption of their interaction interface. Further refinement yielded a map of the protein complex at 4.0 Å, where we find an equivalent hexameric arrangement of the PDs as in the LRRC8A/D/Sb1^c1^ complex (superimposing with an RMSD of 1.3 Å) but strongly increased heterogeneity in the LRRDs (Fig. 5d and Supplementary Figs. 8, 9a). In this map, a single well-defined LRRD of an A subunit is resolved, with equivalent conformation as found in the r-position of the complex with Sb1 (Figs. 3a and 5d). Its density clearly displays Sb4 binding to the same epitope as defined in the X-ray structure, whereas the other domains and also part of the connecting ISDs are not resolved as a consequence of their increased mobility (Fig. 5a, d). In these cases, residual envelopes become apparent in a blurred map at lower contour (Fig. 5d). While the limited resolution of the PD and the disorder of most LRRDs has prevented a definitive assignment, we have arbitrarily allocated the A2 position to the subunit with observed Sb4 density and numbered the other subunits according to their position in the LRRC8A/D/Sb1^c1^ complex (Figs. 2b, and 5d).

Different from the poorly ordered LRRDs, the densities of all six PDs are well-defined and of sufficiently high quality to permit the interpretation by a model of the protein (Fig. 5d and Supplementary Fig. 9b). Despite the strong influence of sybody-binding on the structural organization of the LRRDs, these PDs show a very similar organization as in the respective LRRC8A/D/Sb1 structures, particularly in their extracellular parts, including the ESD and the TM region (Fig. 5d, e, Supplementary Fig. 9a, b). Remarkably, the ESD harboring the pore constriction and conferring selectivity does not carry any detectable difference from the equivalent Sb1 complex, confirming previous studies, which have defined this region as the structurally most invariant part of the channel^21,43^ (Fig. 5e, and Supplementary Fig. 9a). No conclusion can be drawn with respect to differences between the N-termini encompassing the first 14 residues, which are not defined in either structure but whose mutations have previously been shown to exert a strong influence on channel activity^39^ (Fig. 4d and Supplementary Fig. 9c). In contrast to the well-defined ESD and TM regions, the ISDs connecting to the LRR domains are in most subunits less well resolved, reflecting the higher mobility induced by their proximity to the mobile LRRDs (Supplementary Fig. 9b, c).

Although the LRRC8A/D/Sb4 complex carries features of an activated state, the unaltered effects on the membrane-inserted and extracellular parts of the PD is puzzling, as it does not reveal obvious conformational changes in the pore region that might release a gate to open the ion conduction pathway. Such changes would be particularly relevant in the case of VRACs carrying the LRRC8D subunits, which conduct larger substrates such as amino acids, osmolytes and anti-cancer drugs^16,18^, and which might require a cellular membrane environment to be fully adopted. Still, the binding of Sb4 has led to a clear rearrangement of the tight interactions between A domains, and in light of its potentiating effect on channel activity, our data have reiterated the close relationship between LRRD mobility and channel activation that while apparent for LRRC8A also extends to other subunits as observed in previous studies^21,35,36,38,43^.

## Discussion

**Fig. 6:**
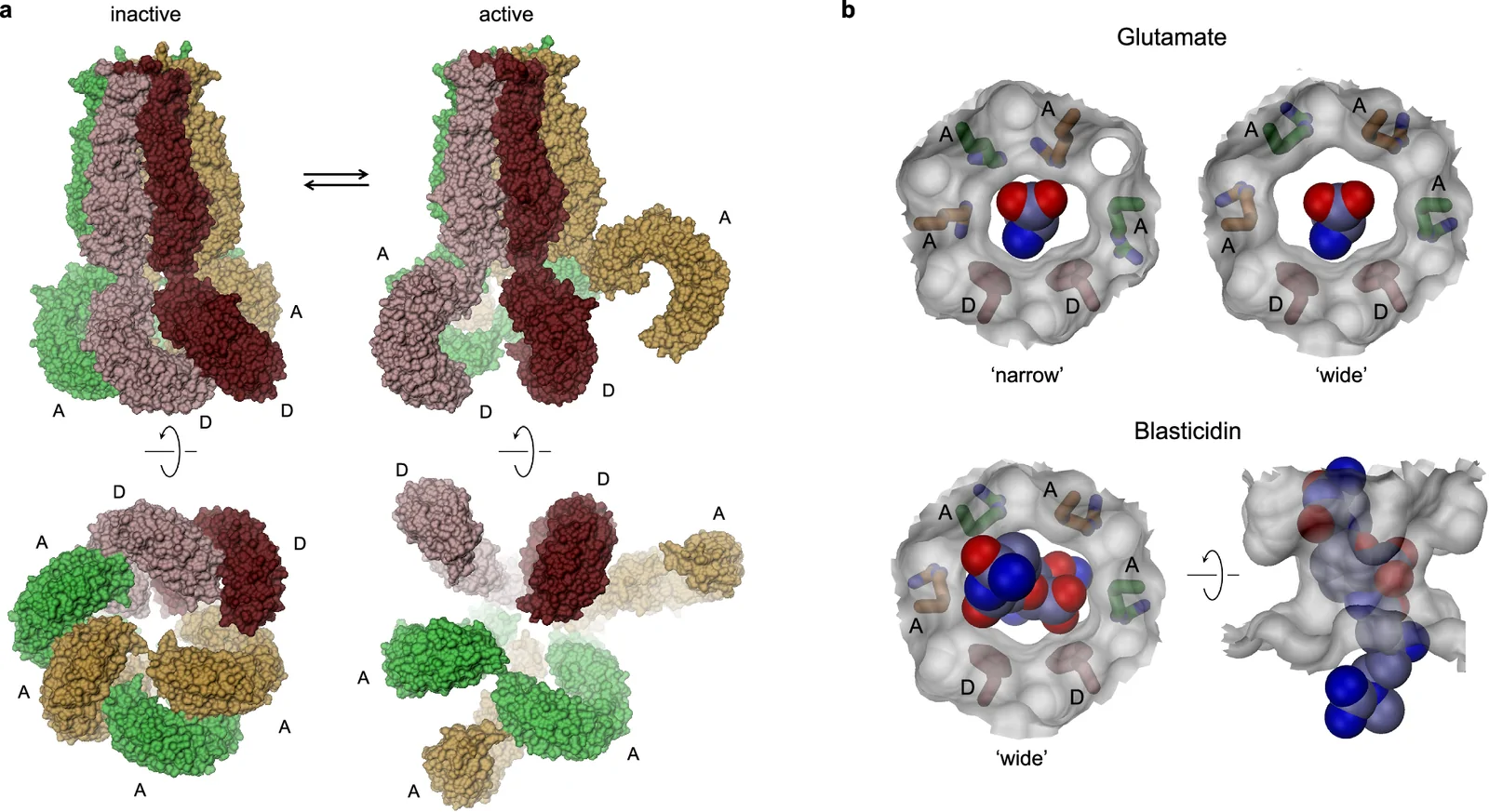
Presumed permeation and gating mechanisms. **a** Schematic model of LRRC8 activation with molecular surface of the channel part of the LRRC8A/D/Sb1^c1^ complex representing an inactive conformation and an approximate model with LRRDs positioned into the low-resolution envelope shown in Fig. 6b representing the activated state. **b** Model of the selectivity filter of the LRRC8A/D channel represented as molecular surface of the pore region superimposed on residues lining the filter and putative permeating substrates shown as space filling model. Top, representation of the pore in the refined structure (left, ‘narrow’) and a potential maximal expansion of the pore obtained by conformational changes of the lining Arg residues (right, 'wide') with the permeant glutamate shown for size comparison. Bottom, relationship between the hypothetical maximally expanded pore and blasticidin S, a proposed VRAC substrate, whose dimensions appear to exceed the observed diameter, viewed from the extracellular side (left) and from within the membrane (right).

Our study on the structural properties of LRRC8A/D defined the subunit organization of this heteromeric anion channel and revealed features of inhibited and activated states. These states were stabilized by two synthetic nanobodies (sybodies), which both specifically target distinct regions in the LRRD of the A subunit^38^. In case of Sb1, this interaction represses, and in case of Sb4, it potentiates LRRC8A/D currents that were evoked in response to the exposure of cells to hypotonic conditions, as demonstrated by patch clamp electrophysiology (Fig. 1b, c). These findings suggested that their addition to the purified channel would influence its conformational properties even in detergent solution. The subunit assignment of the LRRC8A/D complex was facilitated by the application of the inhibitory sybody Sb1 (Fig. 3a and Supplementary Fig. 6a), which rigidifies the LRRDs of the A subunits in a conformation resembling their structure in the homomeric LRRC8A/Sb1 complex^38^. The observed 2:1 stoichiometry of A to D subunits and their distinct arrangement in the hexameric assembly is in agreement with a recent investigation of an equivalent LRRC8A/D complex containing a genetically modified A subunit to facilitate subunit assignment^42^. It also resembles a previous study of a complex consisting of A and C subunits showing a similar arrangement^21^. In the latter, the conformational mismatch of its constituents, with LRRC8C on its own assembling as heptamer^21,28^, has even further disturbed the organization in the heteromeric channel. This disturbance is reflected in the poorly defined ISDs and the absent density of the LRRDs of the C subunits^21^. In all cases, the altered preferred arrangement of the less abundant C and D subunits destabilizes the tight pairwise interaction of A subunits, which is pronounced at the level of their LRRDs, thus increasing the activation properties of the complex (Fig. 3a, b, and Supplementary Fig. 6a, b). Activation is accompanied by a successive destabilization of the LRR domains (Fig. 6a), which has also been observed in an accompanying manuscript describing the properties of point mutations at the interface between the LRRDs and the transmembrane pore^44^. The described increase in domain mobility is emphasized in the structure of the heteromeric LRRC8A/D channel in complex with the activating sybody Sb4, which was added in stoichiometric excess. This binder occupies an epitope that in the resting state of the channel is buried in the tight interface between the LRRDs of the A subunits, while it is accessible in alternate loose interfaces^38^ (Fig. 5a). In our structure of the LRRC8A/D/Sb4 complex, which was determined at a decreased salt concentration to further promote activation, the tight interface between the LRRD of A subunits was presumably released with all binding sites being occupied by Sb4. Consequently, we find a pronounced movement of single LRRDs away from the channel axis, which is manifested in certain 2D and 3D classes (Fig. 5b, c). The increased LRRD mobility is leading to the disappearance of their density upon further map refinement, except from a single subunit, residing in an equivalent conformation as observed for the r position of LRRC8A pairs (Fig. 5c, d). The pronounced tilt of single LRRDs towards the membrane resembles an equivalent conformation observed in the structure of a point mutant in the ISD of LRRC8A with strongly activating phenotype^44^. This further strengthens the link between LRRD mobility and channel activity^21,35,38,43^ and points towards a potential membrane interaction of the strongly inclined LRRD that still needs to be confirmed. However, despite the described structural correlations, the subsequent conformational changes leading to the opening of a gate that impedes ion permeation in closed conformations have remained elusive, as no obvious differences in the membrane-inserted domain constituting the pore were found between different structures (Supplementary Fig. 9a). We thus expect that the LRRC8A/D/Sb4 complex displays a potential intermediate towards activation rather than a fully activated conformation. In this respect, the disordered density inside the channel that is visible in blurred maps immediately above the well-defined region of the PD is striking (Fig. 4d), as it might correspond to the initial 14 residues at the N-terminus of the protein that are not resolved in any of our determined maps and that have previously been shown to influence channel properties^39^. As this N-terminus was observed in certain structures of LRRC8A^27^ and LRRC8D^29^ homomers to line the wall of the pore in extracellular direction, a correlated change in conformation that is related to channel activity appears a plausible scenario. Another feature in the pore concerns weak density below the extracellular ESD (Fig. 4d), which was assigned to bound lipids playing a potential role in channel gating in other studies^22,42^. Here, we refrain from such an assignment due to the limited resolution of the maps and the lack of supporting data.

Our structures of LRRC8A/D channels have also defined the organization of the ESD acting as a selectivity filter of VRACs^23^. The rigid Phe sidechains in consecutive D subunits replacing the long and flexible arginine at the equivalent position of LRRC8A have altered the shape of the pore constriction (Fig. 4a, b). The consequent changes in the chemical properties of the filter, combined with alternative conformations of the remaining arginine side-chains of A subunits, could transiently expand the filter to permit the permeation of larger substrates such as glutamate^16,45^ (Fig. 6b). A similar filter expansion by side chain rearrangements observed in a recent study of the heptameric pannexin PNX1 was proposed to underlie the permeation of ATP in this large-pore channel^46^. However, while such expanded pore geometry in LRRC8A/D channels would be generally compatible with substrates up to the size of acidic amino acids, the dimensions of larger molecules that were also ascribed as substrates of certain VRAC channels, such as blasticidin S^19^, ATP^47,48^ and the dinucleotide cGAMP^11,49^ could even exceed the increased pore diameter resulting from side-chain rearrangements (Fig. 6b). These substrates might either require a strongly altered pore conformation, due to conformational changes in the filter backbone, or a larger oligomeric organization as found in homomeric LRRC8C channels^21,28^. Whereas, in light of the rigid organization of the ESD (Figs. 4a and 5e), large conformational changes in the filter appear unlikely, there is currently also no evidence for a higher oligomeric organization in studies of heteromeric channels composed of two different subunits^21,22,42^ (Figs. 3 and 5). Finally, the poor responsiveness and low current amplitudes of human LRRC8A/D heteromers point towards a possible organization of heteromeric VRACs as assemblies containing more than two different LRRC8 subunits, which was proposed in previous studies^16^. These questions will be subject to future investigations for which the current study provides a solid foundation.

## Methods

### Expression constructs

The constructs encompassing murine LRRC8A, and its cytoplasmic domains LRRC8A^LRRD^ and LRRC8D^LRRD^ were obtained from previous studies^23,38^. Additional constructs were generated by FX cloning^50^. A construct containing the full-length mouse LRRC8D was generated from a synthetic gene coding for the protein (obtained from Genscript). The cytoplasmatic domains LRRC8A^LRRD^ and LRRC8D^LRRD^ were expressed as fusion constructs preceded by an N-terminal Streptavidin-binding peptide (SBP) tag, a myc tag, Venus and a Rhinovirus 3C protease-cleavable linker (3C cleavage site) in a pcDx vector (pcDxn3VMS). For transient overexpression, full-length LRRC8A was cloned into pcDx vectors containing a C-terminal 3C cleavage site followed by mScarlet1, a myc tag and a His10-tag (pcDxc3SMH). Full-length LRRC8D was cloned into a pcDx vector containing a C-terminal 3C cleavage site followed by Venus, a myc tag and an SBP tag (pcDxc3VMS) for transient transfection or into a pEZT-BM vector^51^ containing the same C-terminal 3C cleavage site followed by Venus, a myc tag and an SBP tag (pEZT-BM_C3VMS)^52^ for viral transfection using the BacMam system^53^. For electrophysiology, the above-mentioned murine constructs were used in addition to constructs from synthetic genes coding for human full-length LRRC8A and LRRC8D subunits (GenScript). For electrophysiology experiments, all LRRC8A and LRRC8D constructs were subcloned into pcDx vectors containing a 3C cleavage site followed by a myc tag and an SBP tag (pcDxc3MS). Sb1 in pcDxc3VMS and a Venus-only construct used for co-transfection for patch-clamp recordings contained the Venus gene followed by a myc tag and an SBP tag. For periplasmatic expression of Sb1 and Sb4 in *E.coli*, sybodies were cloned into an L-arabinose-inducible vector containing an N-terminal pelB signal sequence and a His10-tag followed by a 3C cleavage site (pBxnPH3).

### Cell culture

HEK293SGnTl^-^ (CRL-3022) were obtained from ATCC. HEK293 LRRC8^-/-^ and HEK293 LRRC8^B,C,E-/-^ cells were kindly provided by T.J. Jentsch.

HEK293GnTl^-^ and HEK293 LRRC8^-/-^ cell lines were adapted to suspension culture and grown in HyCell HyClone TransFx-H medium (Cytivia) supplemented with 1% fetal bovine serum (FBS, Sigma), 4 mM L-glutamine (Gibco), 100 U/ml penicillin-streptomycin (Sigma) and 1.5% (w/v) Kolliphor P188 (Sigma) at 37 °C and 5% CO_2_ in an orbital shaker incubator (Kuhner). For electrophysiology, adherent HEK293 LRRC8^-/-^ and LRRC8^B,C,E-/-^ were grown in high-glucose DMEM medium (Gibco) supplemented with 10% FBS, 4 mM L-glutamine, 1 mM sodium pyruvate and 100 U/ml penicillin-streptomycin (Sigma) at 37 °C, and 5% CO_2_.

### Protein expression

LRRC8A/D: HEK293 LRRC8^-/-^ suspension cultures were seeded to 0.5 10^6^ cells/ml 24 h prior to transient transfection. The following description refers to the transfection for 300 ml suspension culture. For transfection 1.2 µg of total DNA per 10^6^ cells was diluted in 25 ml high-glucose DMEM media (Gibco) supplemented with 4 mM L-glutamine and 1 mM sodium pyruvate. The ratio (w/w) of LRRC8A in pcDxc3SMH and LRRC8D in pcDxc3VMS was kept at 1:1.5 for all transfections. 3 µg polyethyleneimine (PEI_MAX_ 40 kDa) per 10^6^ cells was diluted separately in 25 ml high-glucose DMEM media (Gibco) supplemented with 4 mM L-glutamine and 1 mM sodium pyruvate. Both solutions were initially incubated at room temperature (RT) for 5 min, mixed and incubated for another 20-30 min. After incubation, 300 ml of HEK293 LRRC8^-/-^ suspension culture was supplemented with the transfection mixture containing DNA and PEI and supplemented with 4 mM valproic acid. Transfected cells were harvested 48-60 h post transfection by centrifugation at 600 g for 15 min at 4 °C. Pellets were washed twice with ice-cold TBS and collected by centrifugation at 600 g for 15 min at 4 °C. After the second washing step, pellets were flash frozen in liq. N_2_ and stored at −80 °C until further use.

LRRC8D: LRRC8D was expressed in LRRC8^-/-^ cell lines using the BacMam system. To prepare bacmid, LRRC8D in pEZT-BM_C3VMS was transformed into heat-competent DH10Bac *E.coli*. Transformed DH10Bac *E.coli* was grown on LB-agar plates supplemented with 50 µg/ml kanamycin, 7 µg/ml gentamycin, 10 µg/ml tetracycline, 100 µg/ml bluo-gal and 40 µg/ml IPTG for blue-white colony selection. After incubation at 37 °C for 48 h, white colonies were selected and cultured. Finally, bacmid was isolated using a QIAprep Spin Miniprep Kit (QIAGEN) followed by DNA precipitation. For the generation of the baculovirus carrying the full-length LRRC8D construct (BacMam virus), Sf9 cells in log phase of growth (1.5 – 2.5×10^6^ cells/mL) were seeded to 0.75×10^6^ cells per well into six-well plates and transfected with purified bacmid using Cellfectin II reagent (Thermo Fisher Scientific). After 4-5 days, the supernatant was filtered and diluted at a ratio of 1:800 to Sf9 cells in suspension at a cell density of 10^6^ cells per ml. After 48 h, the virus-containing supernatant was filtered and stored at 4 °C protected from light. For transduction with the BacMam virus, a HEK293 LRRC8^-/-^ suspension culture was diluted to a cell density of 10^6^ cells per ml. After incubation for 24 h, the viral stock was diluted at a ratio of 1:10 into the expression culture and cells were grown at 37 °C for 24 h in an orbital shaker incubator. The transduced suspension culture was supplemented with 10 mM sodium butyrate and incubated for additional 48-65 h. After completed LRRC8D overexpression, cells were harvested by centrifugation at 600 g for 15 min at 4 °C. Cell pellets were washed twice with ice-cold TBS, prior to being flash frozen in liq. N_2_ and stored at −80 °C until further use.

LRRD constructs: Soluble constructs of the cytoplasmic domains LRRC8A^LRRD^ and LRRC8D^LRRD^ were expressed in HEK293GnTl^-^ suspension cells using transient transfection as described for LRRC8A/D.

Sybodies: For expression, Sb1 and Sb4 in pBxNPH3 were transformed into heat-competent *E.coli* MC1061. A pre-culture was grown in *Luria-Broth* (LB) medium overnight using ampicillin (100 µg/mL) as selection marker. For overexpression, the preculture was diluted 1:100 into *Terrific-Broth* (TB-Gly) medium and cells were grown in a shaker-incubator at 37 °C, to an OD_600_ between 0.8–1.0. Overexpression was induced by addition of L-arabinose at a final concentration of 0.02% (w/v) and the culture was incubated at 22 °C for 16-18 h. Cells were harvested by centrifugation at 5000 g for 20 min at 4 °C. Pellets were flash frozen in liq. N_2_ and stored at −20 °C until further use.

### Protein purification

For the purification of the membrane proteins LRRC8D and LRRC8A/D, all steps were carried out at 4 °C. Cell pellets were thawed and resuspended in extraction buffer (25 mM Tris-HCl pH 8.5, 250 mM NaCl, 2% (w/v) digitonin, 0.05 µg/mL DNaseI, 1 µg/ml Leupeptin, 1 µg/ml Pepstatin, 1 µg/ml Benzamidin, 1 µg/ml PMSF) at a ratio of 30 ml buffer per l of expression culture. LRRC8D and LRRC8A/D were extracted by incubation for 2 h under gentle agitation. The extraction mixture was cleared by centrifugation at 85’000 g for 30 min and subsequently filtered through 5 µm filter. The filtered lysate was mixed with equilibrated Strep-Tactin Superflow resin (1 ml/l of expression culture; high-capacity resin, IBA LifeSciences) and mixed for 1.5-2 h under gentle agitation. The incubated resin was collected and washed with 40 column volumes (CV) of wash buffer (25 mM Tris-HCl, pH 8.5, 250 mM NaCl, 0.1% (w/v) digitonin). Subsequently, LRRC8D-containing VRAC channels were stepwise eluted with elution buffer (25 mM Tris-HCl, pH 8.5, 250 mM NaCl, 0.1% (w/v) digitonin, 10 mM d-Desthiobiotin). Protein-containing fractions were pooled and concentrated to a final volume of 3-5 ml using Amicon Ultracentrifugal concentrators (100 kDa cut-off, Ultracel). The concentrated fraction was supplemented with 1.6 mg of human-rhinovirus (HRV)3 C protease and incubated for 1 h under gentle agitation to remove the C-terminal tag and further concentrated to 500 µl. The concentrated sample was separated on a Superose S6 10/300 GL column (GE Healthcare) that was equilibrated with SEC buffer (25 mM Tris-HCl, pH 8.5, 250 mM NaCl, 0.1% (w/v) digitonin). Fractions corresponding to hexameric LRRC8D or LRRC8A/D assemblies were pooled and concentrated to 3.5–4.5 mg/ml using Amicon Ultracentrifugal concentrators (100 kDa cut-off, Ultracel). The sample of the LRRC8A/D channel purified via a single-step affinity chromatography via the tagged LRRC8D subunits, still contained a fraction of LRRC8D homomers. The LRRC8D fraction was estimated to amount for 40% of total LRRC8 protein isolated during the single-step affinity chromatography. The fraction of LRRC8D was estimated by following the relative fluorescence of differentially tagged subunits and the observation that the expression levels of LRRC8D is usually ~50% of LRRC8A. The detergent glycol-diosgenin (GDN, Anatrace), has replaced digitonin at a concentration of 2% during extraction and 0.05% during further purification steps for the preparation of LRRC8D and the LRRC8A/D/Sb4 complex.

LRRC8D was directly applied on cryo-EM grids at a concentration of 4.5 mg/ml. In case of the LRRC8A/D/Sb1 complex, concentrated fractions of LRRC8A/D were supplemented with a 1.5 molar excess of Sb1 (per LRRC8 subunit) and incubated for 30 min on ice before application on cryo-EM grids. For the preparation of the LRRC8A/D/Sb4 complex, the concentrated LRRC8A/D fraction was diluted with dilution buffer (25 mM Tris-HCl pH 8.5, 0.05% (w/v) GDN) to a final NaCl concentration of 100 mM in the sample. After an additional concentration step, the LRRC8A/D sample was supplemented with a 3x molar excess of Sb4 (per LRRC8 subunit) and incubated on ice for 30 min prior to grid freezing.

Purification of LRRDs: For the preparation of the LRRDs of LRRC8A and LRRC8D, all purification steps were performed at 4 °C. For preparation of the LRRD of LRRC8D, cell pellets were thawed and resuspended in lysis buffer (20 mM Hepes pH 7.4, 150 mM NaCl, 2 % (w/v) n-dodecyl-β-D-maltoside (DDM), 0.05 µg/ml DNaseI, 1 µg/ml Leupeptin, 1 µg/ml Pepstatin, 1 µg/ml Benzamidin, 1 µg/ml PMSF at a ratio of 30 ml of buffer per l of expression culture). The suspension was incubated for 1 h under gentle agitation, cleared by centrifugation at 85’000 g, for 30 min and subsequently filtered through a 5 µm filter. The cleared lysate was incubated with pre-equilibrated Strep-Tactin Superflow resin (high capacity, IBA LifeSciences, at a ratio of 1 ml of resin per l of expression culture) for 1.5 h under gentle agitation. Subsequently, resin was collected and washed with 40 CV of wash buffer (20 mM Hepes, pH 7.4, 150 mM NaCl, 0.013% (w/v) DDM). To elute the LRRD construct of LRRC8D, the resin was supplemented with additional 2 CV of wash buffer and HRV-3C protease at a final concentration of 0.1 mg/ml and incubated for 1 h under gentle agitation. After incubation, the eluate was collected and the resin was washed with an additional CV of wash buffer. The eluted protein was concentrated to a final volume of 500 µl using an Amicon Ultracentrifugal concentrator (35 kDa cut-off, Ultracel) prior to its separation on a Superdex S75 10/300 GL column (GE Healthcare) incubated with SEC buffer (20 mM Hepes pH 7.4, 150 mM NaCl, 0.013% (w/v) DDM). Monomeric protein fractions were pooled and concentrated to a final concentration of 8–12 mg/ml for crystallization.

The preparation of the LRRD of LRRC8A proceeded with the same protocol with minor modifications. The cleared lysate was mixed with pre-equilibrated Strep-Tactin Superflow resin (high capacity, IBA LifeSciences at a ratio of 2 ml/l of expression culture) and incubated for 1.5 h under gentle agitation. The resin was collected and washed with 10 CV of wash buffer (10 mM Tris-HCl pH 8.5, 200 mM NaCl, 0.013% DDM). The protein was retrieved by stepwise elution with elution buffer (10 mM Tris-HCl, 200 mM NaCl, 0.013% DDM, 5 mM Desthiobiotin). Elution fractions containing the protein were pooled and supplemented with HRV-3C protease at a 1:5 molar ratio, incubated for 30 min under gentle agitation and concentrated to 500 µl using Amicon Ultracentrifugal filters (35 kDa cut-off, Ultracel). Subsequently, the concentrated fraction was separated on a Superdex S75 10/300 GL column (GE Healthcare) equilibrated in SEC buffer (10 mM Tris, pH 8.5, 200 mM NaCl, 0.013% (w/v) DDM). Peak fractions were pooled and concentrated using Amicon Ultracentrifugal filters (35 kDa cut-off, Ultracel).

Sybodies Sb1 and Sb4: All purification steps of the sybodies Sb1 and Sb4 were performed at 4 °C. Pellets from overexpression culture were thawed and resuspended in lysis buffer (50 mM Tris, pH 7.5, 150 mM NaCl (TBS), 0.05 mg/ml DNase, 1 µg/ml Leupeptin, 1 µg/ml Pepstatin, 1 µg/ml Benzamidin, 1 µg/ml PMSF). The volume of the resuspension was adjusted to an extrapolated OD_600_ of 50. Cells were disrupted using a high-pressure cell lyser (Maximator, HPL6) and subsequently cleared by centrifugation at 8000 g for 30 min. The cleared lysate was supplemented with imidazole at a final concentration of 15 mM before incubation with pre-equilibrated Ni-NTA resin (1 ml/30 ml sample, Agarose Bead Technologies) for 1.5 h under gentle agitation. After incubation, the resin was collected and washed with 30 CV of wash buffer (TBS, 50 mM imidazole). Bound sybodies were eluted from the Ni-NTA resin by stepwise application of elution buffer (TBS, 300 mM imidazole). The most enriched elution fractions were pooled and supplemented with HRV-3C protease at a 1:5 molar excess and dialysed against TBS overnight. The His-tagged HRV-3C protease and the cleaved His-tag were removed by binding to Ni-NTA resin (Agarose Bead Technologies). Prior to incubation with pre-equilibrated Ni-NTA resin (1 ml/30 ml of sample, Agarose Bead Technologies) for 30 min under gentle agitation, the dialyzed sample was supplemented with imidazole at a final concentration of 30 mM. The flowthrough (FT) was collected and the resin was washed with 2 CV TBS. FT and wash fractions were pooled and concentrated to 2 mg/ml using Amicon Ultracentrifugal filters (10 kDa cut-off, Ultracel). The concentrated sybody fraction was injected into a Sepax SRT-10C SEC100 column (Sepax Technologies) pre-equilibrated in SEC buffer (TBS, 0.5 mM EDTA). Fractions containing monomeric sybody were pooled and concentrated to a final concentration of 4 mg/ml using Amicon Utracentrifugal filters (10 kDa cut-off, Ultracel). Concentrated sybody fractions were supplemented with glycerol at a final concentration of 20% (v/v), flash frozen in liquid N_2_ and stored at −80 °C until further use.

### Crystallization and X-ray crystallography

LRRD of LRRC8D: The purified LRRD of LRRC8D was concentrated to 8–12 mg/ml and used to prepare vapor diffusion crystallization experiments in sitting drops by mixing 200 nl of concentrated protein with 200 nl of reservoir solution containing 0.2 M malonate and 20% (w/v) PEG 3350 and equilibrating against the reservoir. Crystals grew at 4 °C within a week. The grown crystals were fished and flash frozen in liq. N_2_ without further cryo-protection. X-ray diffraction data were collected on frozen crystals on the X06SA beamline at the Swiss Light Source at the Paul-Scherrer Institute on a EIGER 16 M detector (Dectris). Indexing, integration and scaling was performed in XDS^54^ yielding a complete dataset at 1.96 Å (Table 2). The crystals were of Space group P2_1_2_1_2_1_ and contained one copy of the LRRD in the asymmetric unit. Initial phases were obtained by molecular replacement with the program Phaser^55^ implemented in the Phenix suite^56^ using the structure of the LRRD of LRRC8A (PDB 6FNW) as search model. The structure was improved by iterative cycles of manual rebuilding in Coot^57^ followed by refinement in PHENIX^56^ (Table 2).

LRRD of LRRC8A in complex with Sb4: For preparing a sample for crystallization, the purified and concentrated LRRD of LRRC8A was supplemented with a 1.5 molar excess of Sb4 and incubated for 5 min on ice before separation on a Superdex S75 10/300 GL column (GE Healthcare) equilibrated with SEC buffer (10 mM Tris pH 8.5, 200 mM NaCl, 0.013% (w/v) DDM). Peak fractions of the LRRD/Sb4 complex were concentrated using Amicon Ultracentrifugal filters (10 kDa cut-off, Ultracel) to a final concentration of 8-10 mg/ml for crystallization screening.

Crystallization proceeded by vapor diffusion experiments in sitting drops with 100 nl of concentrated protein complex mixed with 100 nl of reservoir solution containing 20% (w/v) PEG 4000, 10 % (v/v) 2-PropOH and 0.1 M HEPES, pH 7.5 and equilibrated against the reservoir. Crystals grew at 4 °C within a week. For cryo-protection crystals were harvested and transferred to a solution composed of the mother liquor supplemented with 0.25 mM DDM and 35 % of ethylene glycol and flash frozen in liquid N_2_. X-ray diffraction data was collected on frozen crystals on the X06DA beamline at the Swiss Light Source at Paul-Scherrer Institute on a PILATUS 2M-F detector. All data sets were indexed, integrated, scaled and merged using XDS^54^. The crystals were of Space group P1, containing two copies of the LRRD/Sb4 complex in the asymmetric unit, and the data extended to 1.6 Å (Table 2). Phases were obtained by molecular replacement in Phaser^55^ using the LRR domain of LRRC8A (PDB ID: 6FNW) as search model. Sb4 was inserted into the complex in alternating cycles of manual model building in Coot^57^ and refinement in Phenix^56^.

### Cryo-EM sample preparation and data collection

For cryoEM sample preparation 2.5 µl aliquots of each sample were applied onto glow-discharged Quantifoil R1.2/1.3 Au 300 mesh grids. Each grid was blotted with blot force of 0 for 2-5 s at 4 °C and 100% humidity and plunge-frozen in ethane-propane gas mix using VitroBot Mark IV (Thermo Fisher Scientific).

Datasets for LRRC8A/D in complex with Sb1 and LRRC8D were collected on a 300 kV Titan Krios G3i (ThermoFisher Scientific) equipped with a 100 µm objective aperture, a 50 µm C2 aperture, a post-column BioQuantum energy filter with a 20 eV slit and a K3 direct electron detection camera (Gatan). All data sets were collected in pseudo super resolution mode using EPU versions v2.9-v3.9 at a defocus range of −1 to −2.4 µm and a magnification of 130’000x corresponding to 0.651 Å/pixel. Each micrograph was exposed for 1.26 s corresponding to a total of 47 frames. Two data sets of LRRC8A/D with Sb1 were collected at total doses of 59.64 e^-^/Å^2^ and 63.03 e^-^/Å^2^, respectively. The data set for LRRC8D was collected with a total dose of 61.53 e^-^/Å^2^.

Data of LRRC8A/D in complex with Sb4 were collected on a 200 kV Glacios G2 cryo electron microscope (ThermoFisher Scientific) with a 20 µm C2 aperture and a Falcon 4i direct electron detection camera. Data were automatically collected using EPU v3.9 with application of AFIS for faster acquisition. All micrographs were collected at −0.8 to −2.6 µm defocus range and a magnification of 190’000x corresponding to 0.72 Å/pix. The data set of LRRC8A/D with Sb4 was collected at a total dose of 60.65 e^-^/Å^2^.

### CryoEM data processing, model building and refinement

In all cases, cryo-EM data was processed in cryo-SPARC version 4 (ref. ^58^). All super-resolution images were submitted to patch motion correction with a Fourier crop factor 1 (pixel size of 0.651 Å/pix) and submitted to patch CTF estimation in cryoSPARC. Micrographs with poor CTF fit, high total frame motion drift and low-quality ice thickness were discarded. Structures were built into cryo-EM maps in Coot^57^ and refined in Phenix^59^.

LRRC8D: The data processing of the LRRC8D complex has proceeded as detailed in Supplementary Fig. 3. To generate templates for automated particle picking, an initial particle set was obtained from 27’816 corrected micrographs using blob picker with a particle diameter of 100–250 Å and a minimal separation factor of 0.5. All selected particles were subjected to two rounds of 2D classification. 2D classes with various particle orientations and high-resolution protein features were used as templates for automated template picking. The template 2D classes were submitted to an initial ab-initio reconstruction with 5 classes, which were used in the initial rounds of heterogenous refinement described below. Selected particles from template picking were extracted with a box size of 336 pixels (down-sampled to 168 pix, 2.604 Å per pixel) and subjected to two rounds of 2D classification. 2D classes were separated into two sets, one with high-resolution protein features and a second lacking protein features (decoy). Decoy 2D classes were submitted to an ab-initio reconstruction with 2 classes (decoy volume). Particles of classes with high resolution protein features were submitted to a heterogenous refinement cycle with a decoy volume, a TM-only volume containing particles with poorly ordered LRRDs, and a full-length volume where TM region and LRRD of the channels are well-defined. After each round, particles of the full-length class were used for the subsequent heterogenous refinement step. In the last cycle of heterogeneous refinement, particles of the final full-length protein class were submitted to an ab-initio reconstruction with 3 classes. Particles of each of the three ab-initio volumes and the decoy volume of the final heterogenous refinement step were re-extracted with a box size of 336 pixels (1.302 Å per pixel). After re-extraction, all particles were subjected to separate ab-initio reconstructions serving as inputs for the following heterogenous refinement. The first heterogenous refinement cycle after particle re-extraction included the particles of all three full-length protein classes and the decoy volume. From the three full-length protein volumes, ones with two and six ordered LRRDs contained the majority of particles. Therefore, these particles were combined and resubjected to a final round of heterogenous refinement with a decoy volume and the two volumes containing either two or six ordered LRRDs. In the last step, the final particles contributing to the volume containing six ordered LRRDs were aligned by non-uniform refinement with C1 symmetry applied, yielding a map of LRRC8D at 2.99 Å (Table 1). The model of the TM was rebuilt into the cryo-EM density, starting with a template of the PD of LRRC8A (PDB-ID: 7P5V). For the LRRD, the X-ray structure of the domain determined in this study was placed into the density of subunits D1-3 where the LRRD was well-defined. The resulting hexameric channel containing LRRDs in three position was refined in Phenix. The placement of the LRRDs into the three weakly defined subunits (D4-D6) was based on the refined structure of the D1-D3 subunits followed by another round of refinement.

LRRC8A/D/Sb1: The data processing of the LRRC8A/D/Sb1 complex has proceeded as detailed in Supplementary Fig. 5. An initial particle set was obtained from 45’646 corrected micrographs using blob picker with a particle distance of 120 Å, a circular blob shape and images low pass filtered at 20 Å. From the output of blob picker, particles were extracted with a box size of 366 pixels (down-sampled to 183 pixels, 2.604 Å per pixel) and subjected to two rounds of 2D classification. 2D classes showing high resolution LRRC8 features with clear density of Sb1 were used as templates for automated particle picking. Particles were extracted with a box size of 366 pixels (downsampled to 183 pixels) and subjected to another round of 2D classification. 2D classes were sorted into three subsets, one displaying high resolution LRRC8 features with clear density of Sb1 corresponding to LRRC8A/D channels, a second subset with clear LRRC8 features but lacking Sb1 density corresponding to LRRC8D homomers and a third subset lacking any protein features. Each subset was submitted to separate ab-initio reconstruction with various numbers of classes. Particles from high quality LRRC8A/D 2D classes were subjected to two rounds of heterogenous refinement and sorted into two volumes containing protein density and another volume lacking such features. In the following steps particles of the protein-containing volumes were subjected to additional steps of heterogenous refinement. To follow the progress of heterogenous refinement, protein-containing classes were subjected to a non-uniform refinement step to obtain an estimate of the resolution. As soon as the Nyquist limit was reached, particles were re-extracted with a box size of 366 pixels (1.302 Å/pix) and subjected to a final ab-initio reconstruction. The re-extracted particles and ab-initio volumes were subsequently used in a final heterogenous refinement step. The following non-uniform refinement step with C1 symmetry yielded final maps of the LRRC8A/D/Sb1 complex in two conformations, at 3.02 Å and 3.16 Å, respectively. For model building in Coot, subunits of the respective homomeric complexes (LRRC8A/Sb1 complex PDB-ID: 7P5V, LRRC8D: model determined in this study) were inserted into the density. For the LRRC8A/D/Sb1^c1^ conformation, a pair of tightly interacting LRRC8A subunits in complex with Sb1 were placed into regions showing strong density of both parts of the channel (A1, A2), whereas for the other subunits (A3, A4) the respective PDs were initially placed and the partial structure was used for an initial round of refinement in Phenix. Subsequently, the LRRDs of the weaker defined subunits were introduced and fitted into the low-resolution envelopes. In case of the two D subunits, the domains were obtained from the D2 and D3 positions of the LRRC8D homomer. The full-length protein was finally refined in Phenix. For LRRC8A/D/Sb1^c2^, the refined structure of LRRC8A/D/Sb1^c1^ was placed into the density and the relative orientation of the LRRDs of the four A subunits in complex with Sb1 was adjusted to improve their fit. Due to the lack of clear density even at low contour, the LRRDs of the two D subunits and the C-terminal half of the LRRD in the A2 position were omitted.

LRRC8A/D/Sb4: The data processing of the LRRC8A/D/Sb4 complex has proceeded as detailed in Supplementary Fig. 8. The set of 8’556 micrographs was submitted to blob picker with a particle distance of 120 Å, circular blob shape and images low pass filtered to 20 Å. The particle set from blob picking was extracted with a box size of 336 pixels (downsampled to 168 pixels, 2.604 Å per pixel) and subjected to 2D classification. 2D classes presenting high resolution LRRC8 features were used as templates for automated template picking. Particles were subjected to a second round of 2D classification with classes containing LRRC8 features used for an initial ab-initio reconstruction with 3 classes, two of which contained LRRC8 features. Particles of the two high quality ab-initio volumes were submitted to two steps of heterogenous refinement including a decoy volume and volume containing LRRC8 features obtained from the previous ab-initio reconstruction. As described for LRRC8A/D in complex with Sb1, particles of the class displaying LRRC8 features were re-subjected to three rounds of heterogenous refinement followed by an ab-initio reconstruction and non-uniform refinement. After reaching the Nyquist limit, particles were re-extracted from unbinned images with a 336-pixel box size (1.302 Å per pixel). After re-extraction, particles were subjected to an ab-initio reconstruction followed by a non-uniform refinement with C1 symmetry yielding in a final map of the LRRC8A/D/Sb4 complex at 3.97 Å resolution. This density contained a single resolved LRRD with bound Sb4, which was assigned as the A2 position. For map interpretation, the LRRC8A/D/Sb1^c1^ structure was fitted into the density with the coordinates of Sb1 and the LRRDs of all subunits except for A2 deleted. The position of Sb4 was placed based on the X-ray structure of the LRRD of the A subunit in complex with Sb4 determined in this study, and the structure was refined in Phenix. Figures of maps and models were prepared in DINO (http://www.dino3d.org).

### Electrophysiology

For all electrophysiology experiments, HEK293T, HEK293 LRRC8^B,C,E−/−^ and HEK293 LRRC8^−/−^ cells were seeded into 6 cm Petri dishes at 5% confluency 24 h prior to measurements. For recordings from overexpressed mouse and human LRRC8A/D channels, HEK293 LRRC8^−/−^ were transiently transfected 6-10 h after seeding. For transient transfection of each 6 cm dish, DNA constructs (0.8 µg LRRC8A, 1.6 µg LRRC8D and 1.6 µg Venus-only or Sb1) were diluted in 250 µl of high-glucose DMEM media (Gibco) supplemented with 4 mM L-glutamine and 1 mM sodium pyruvate. 10 µg of PEI_linear_ (25 kDa) was diluted in a separate 250 µl aliquot of high-glucose DMEM media supplemented with 4 mM L-glutamine and 1 mM sodium pyruvate. After 5 min incubation at RT, the two solutions were mixed and incubated at RT for 20-30 min before application to the cells. Currents of overexpressed channels were recorded between 14–28 h post-transfection. All measurements were performed at 20 °C. Patch pipettes were pulled from borosilicate glass capillaries (Sutter, inner diameter of 0.86 mm and outer diameter of 1.5 mm) with a micropipette puller (Sutter) and fire-polished with a Microforge (Narishige). The typical pipette resistance was 2–7.2 MΩ when filled with pipette solution. Seals with a resistance of 4 GΩ or higher were used to establish the whole-cell configuration. Access resistance was between 6–13 MΩ. Data were recorded with an Axopatch 200B amplifier and digitized with Digidata 1440/1550B (Molecular Devices). Analog signals were digitized at 10–20 kHz and filtered at 5 kHz using the in-built four-pole Bessel filter. Data acquisition was performed using the Clampex 10.7/11.7 software (Molecular Devices). Cells were locally perfused using a gravity-fed system.

Experiments to characterize LRRC8A/D in HEK293 LRRC8^B,C,E-/-^ cells and the effect of Sb1 on mouse LRRC8A/D were performed using the Δmannitol buffer system^43^. Under these conditions, the patch pipette was filled with buffer composed of 10 mM HEPES-NMDG, pH 7.4, 150 mM NMDG-Cl, 1 mM EGTA and 2 mM Na_2_ATP (with a total osmolarity of 270 mmol kg^−1^). After break-in into the cell and establishment of the whole-cell configuration, cells were perfused with isotonic buffer (10 mM HEPES-NMDG, pH 7.4, 95 mM NaCl, 1.8 mM CaCl_2_, 0.7 mM MgCl_2_ and 100 mM mannitol, 300 mmol kg^−1^). After 60 s, cell swelling was initiated by switching the perfusion to hypotonic buffer (10 mM HEPES-NMDG, pH 7.4, 95 mM NaCl, 1.8 mM CaCl_2_ and 0.7 mM MgCl_2_, 194 mmol kg^−1^). Currents were monitored in 2 s intervals for 5 min using a ramp protocol illustrated in Supplementary Fig. 1a (15 ms at 0 mV, 100 ms at −100 mV, a 500 ms linear ramp from −100 mV to 100 mV, 100 ms at 100 mV, 200 ms at −80 mV, 1,09 ms at 0 mV). The values at 100 mV, 10 ms after the ramp, are displayed in the activation curves. The described protocol avoids the clamping at positive voltages for longer periods of time, allows the monitoring of the VRAC-characteristic outwardly rectifying current-voltage relationships during activation, and yields maximum currents at positive potential before desensitization. Current-voltage (I-V) relationships after cell swelling were also obtained from a voltage-step protocol (from −100 to 120 mV in 20-mV steps). After the voltage step protocol, cells were perfused with hypotonic buffer for an additional 30 s before switching to isotonic solution for 5 min, initiating cell shrinkage. The inactivation of currents at positive potential was monitored with the same ramp protocol as described above. For measurements in hypotonic conditions, only one cell per dish was used. Data were analyzed using Clampfit 10.7/11.7 (Molecular Devices) and Excel (Microsoft).

All other experiments including ones to characterize the effect of activating Sb4 on HEK293 LRRC8^B,C,E−/−^ cells were performed under Δsalt conditions^43^. Under these conditions, the patch solution was composed of 10 mM HEPES, pH 7.4, 40 mM CsCl, 100 mM Cs-Met-sulfonate, 1 mM MgCl_2_, 1.9 mM CaCl_2_, 5 mM EGTA, 4 mM Na_2_ATP, 290 mmol kg^−1^). For testing the effect of the sybody, the patch pipette solution was further supplemented with 1 µM of purified Sb4. After break-in into the cell and establishment of the whole-cell configuration, cells were perfused with isotonic buffer (10 mM HEPES, pH 7.4, 150 mM NaCl, 6 mM KCl, 1 mM MgCl_2_, 1.5 mM CaCl_2_, 10 mM glucose, 320 mmol kg^−1^) for 5 min to allow diffusion of Sb4 into the cell. To initiate cell swelling, the perfusion buffer was switched to hypotonic buffer (10 mM HEPES, pH 7.4, 105 mM NaCl, 6 mM KCl, 1 mM MgCl_2_, 1.5 mM CaCl_2_, 10 mM glucose, 240 mmol kg^−1^) for 7 min to reach maximum activation. Currents were recorded with the same perfusion, ramp and voltage-step protocols as described above.

The inhibition of VRAC currents by the inhibitor DCPIB (Sigma) was characterized with fully activated currents in the presence of Sb4. In this case, activation proceeded as described above, except that after the voltage-step protocol, the cells were perfused with hypotonic bath solution for additional 30 s before switching to DCPIB-supplemented hypotonic bath solution (10 mM HEPES, pH 7.4, 105 mM NaCl, 6 mM KCl, 1 mM MgCl_2_, 1.5 mM CaCl_2_, 10 mM glucose, 50 µM DCPIB, 240 mmol kg^−1^) for 60 s.

The electrophysiological characterization of human LRRC8A/D and mouse LRRC8A/D channels was performed under Δsalt conditions as described above, with minor adaptations, where the initial isotonic perfusion was reduced to 60 s and the hypotonic perfusion to 5 min.

### Surface expression analysis

To quantify the surface expression of LRRC8A subunits in transfected human and mouse LRRC8A/D channels, HEK293 LRRC8^-/-^ cells were cultured in high-glucose DMEM (Gibco) supplemented with 10% FBS, 4 mM glutamine, 1 mM sodium pyruvate and 100 U ml^–1^ Penicillin-Streptomycin (Sigma) at 37 °C and 5% CO_2_. 24 h prior to transfection, cells were seeded at 40% confluency. The following transfection preparation is described for one 10 ml plate. 4 µg of total DNA were diluted in 500 µl high-glucose DMEM (Gibco) supplemented with 4 mM glutamine, 1 mM sodium pyruvate. The ratio (w/w) of LRRC8A in pcDxc3MS, LRRC8D in pcDxc3MS and Venus-only was kept at 1:2:2. 20 µg of PEI_MAX_ (40 kDa, Polysciences) was diluted in 500 µl high-glucose DMEM (Gibco) supplemented with 4 mM glutamine, 1 mM sodium pyruvate. Both mixtures were incubated at RT for 5 min before mixing. The transfection mixture was incubated at RT for 20–30 min prior to application to cells. Transfected cells were grown for ~20 h. The surface-biotinylation was performed with a Pierce^TM^ Cell Surface Protein Isolation Kit (ThermoFisher Scientific) according to manufacturer's instructions with minor adaptations. For biotinylation, cells from a 10 ml culture were washed twice with 8 ml PBS before 10 ml Sulfo-NHS-SS Biotin solution with a concentration of 0.25 mg ml^–1^ was applied. For non-biotinylated control, cells were incubated with 10 ml of PBS. After 30 min biotinylation at 4 °C, biotinylation reaction was stopped with 500 μl of quenching solution. Cells were harvested by centrifugation at 600 g for 15 min at 4 °C. Cell pellets were washed twice with ice-cold TBS, prior to being flash frozen in liq. N_2_ and stored at −80 °C until further use. Extraction and purification steps were carried out at 4 °C. Each thawed cell pellet was resuspended in 200 µl of the extraction buffer containing 25 mM HEPES pH 7.5, 250 mM NaCl, 2% (w/v) GDN, 0.05 µg/mL DNaseI, 1 µg/ml Leupeptin, 1 µg/ml Pepstatin, 1 µg/ml Benzamidin, 1 µg/ml PMSF and incubated for 2 h under gentle agitation. Insoluble fractions were removed by centrifugation at 14,000 *g* for 30 min. The supernatant was mixed with 100 µl of bed volume NeutrAvidin Agarose resin equilibrated in wash buffer (25 mM HEPES pH 7.5, 250 mM NaCl, 0.05% GDN) and incubated for 2 h under gentle agitation. The flow-through was discarded, and the resin was washed four times with 400 µl of wash buffer. Resin was resuspended in 20 µl of SDS PAGE loading buffer and incubated at 95 °C for 3 min prior to supplementation with DTT at a final concentration of 30 mM. 12 µl of sample was loaded on a Mini-PROTEAN SDS PAGE gel (4-20% gradient, BioRad). After separation by SDS-PAGE, proteins were transferred to a PVDF membrane and analyzed by Western blot using a mouse anti-LRRC8A primary antibody (Sigma, SAB1412855, 1:1000 dilution) and a peroxidase-conjugated goat anti-mouse secondary antibody (Jackson ImmunoResearch, 115-035-146, 1:10,000 dilution). The luminescent signal was developed with ECL substrate (GE Healthcare) and recorded with a Viber Fusion FX7 imaging system. Specific bands were quantified using Fiji^47^.

### Statistics and reproducibility

Electrophysiology data were obtained from the indicated number of biological replicates. Data from overexpression were reproduced with different transfections. Traces were selected based on the criteria described in the electrophysiology section, and measurements meeting this criterion were included in the evaluation regardless of the magnitude of the current response. Conclusions of experiments were not changed upon inclusion of further data. Values of individual traces were averaged, and the displayed errors are s.e.m. Averaged values and errors were compared by two-tailed unpaired *t* testing with applied Welch correction. Statistical analysis was performed using GraphPad Prism 11.

### Reporting summary

Further information on research design is available in the Nature Portfolio Reporting Summary linked to this article.

## Supplementary information

Supplementary Information

Reporting Summary

Transparent Peer Review file

## Source data

Source Data

## Data Availability

The three-dimensional cryo-EM density maps generated in this study have been deposited in the Electron Microscopy Data Bank under accession numbers EMD-55589 (https://www.ebi.ac.uk/emdb/EMD-55589) (LRRC8D), EMD-55590 (https://www.ebi.ac.uk/emdb/EMD-55590) (LRRC8A/D/Sb1^c1^), EMD-55591 (https://www.ebi.ac.uk/emdb/EMD-55591) (LRRC8A/D/Sb1^c2^) and EMD-55592 (https://www.ebi.ac.uk/emdb/EMD-55592) (LRRC8A/D/Sb4). Coordinates for the models of LRRC8D, LRRC8A/D/Sb1^c1^, LRRC8A/D/Sb1^c2^ and LRRC8A/D/Sb4 have been deposited in the Protein Data Bank under accession numbers 9T5M, 9T5N, 9T5O and 9T5P, respectively. Coordinates and structure factors of the X-ray structures of the LRRD of LRRC8D and the LRRC8A/Sb4 complex generated in this study have been deposited in the PDB under accession number 9T5R and 9T5Q, respectively. All other data needed to evaluate the conclusions of the study are present in the paper or in the supplementary materials. Source data are provided with this paper.
