# Supplementary material for: Structures of the volume-regulated anion channel LRRC8A/D in activating and inhibiting conditions
Source: Nat Commun. 2026 Sep 17;17:9884. doi: 10.1038/s41467-026-77508-x (PMC13586245; doi:10.1038/s41467-026-77508-x)
Supplement: Supplementary file 1 [file 41467_2026_77508_MOESM1_ESM.pdf]

Supplementary Information

Structures of the volume-regulated anion channel LRRC8A/D in activating and inhibiting conditions

Elena F. Lehmann, Fabienne Stierli, Sonja Rutz, Dawid Deneka, and Raimund Dutzler

Supplementary Table 1: Electrophysiology recording conditions.

Figure	Cell System	Buffer System	Sybody application
Fig. 1a	$LRRC8^{B,C,E-/-}$	Δmannitol	-
Fig. 1b	mLRRC8A/D overexpressed	Δmannitol	-
Fig. 1b	mLRRC8A/D overexpressed	Δmannitol	Sb1 overexpressed
Fig. 1c, Suppl. Fig. 1e	$LRRC8^{B,C,E-/-}$	Δsalt	-
Fig. 1c, Suppl. Fig. 1f	$LRRC8^{B,C,E-/-}$	Δsalt	Sb4 patch pipette
Fig. 1d, Suppl. Fig. 1i	$LRRC8^{B,C,E-/-}$	Δsalt	Sb4 patch pipette
Suppl. Fig. 1b	mLRRC8A/D overexpressed	Δsalt	-
Suppl. Fig. 1b	hLRRC8A/D overexpressed	Δsalt	-
Suppl. Fig. 1d, g	HEK 293	Δsalt	-
Suppl. Fig. 1d, h	HEK 293	Δsalt	Sb4 patch pipette

Supplementary Figures

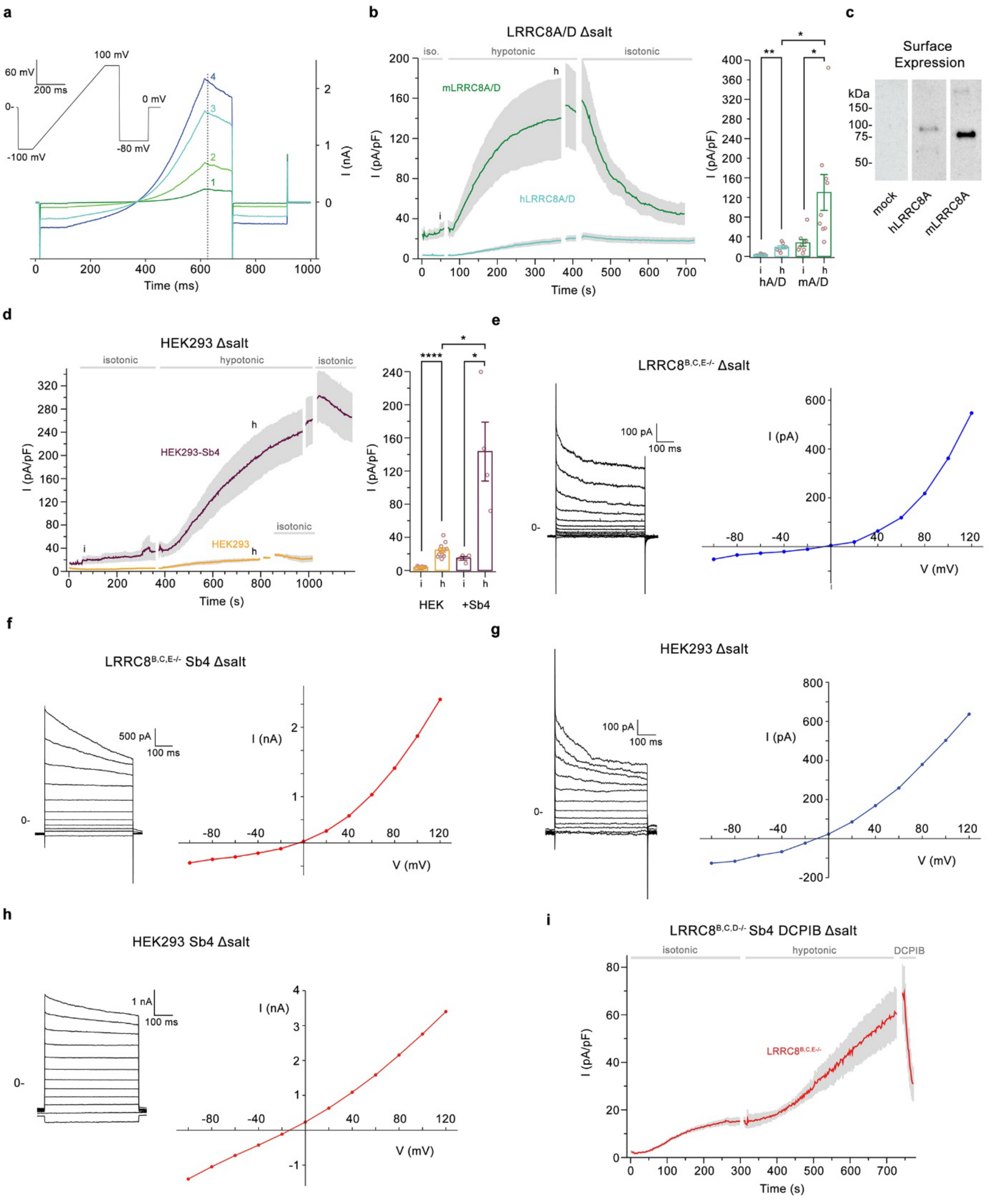

Supplementary Fig. 1: Electrophysiology. a, Representative LRRC8A/D current traces upon cell swelling recorded by a continuous series of ramp protocols. The recordings show representative responses from LRRC8$^{B,C,E-/-}$ cells in presence of Sb4 shown in Fig. 1c at increasing time points after perfusion of hypotonic solutions (1-4). Dashed line indicates time point plotted in traces shown in Fig. 1a-d and Supplementary Fig. 1b, d, i. Inset shows voltage protocol. **b,** VRAC current response to the osmotic swelling of cells. LRRC8$^{-/-}$ cells transfected with constructs of human (cyan, n=7, *p*=0.0031)

and mouse (green, n=9, p=0.23) LRRC8A and D constructs recorded in Δsalt buffers at indicated time points. The difference between activating responses of mouse and human channels is statistically significant (p=0.156). Traces, left, show the mean of 7 (hAD) and 8 (mAD) representative recordings. **c**, Western blots showing the relative expression levels of human and mouse LRRC8A subunits at the plasma membrane from an equivalent number of cells after surface biotinylation. LRRC8A levels were detected with an anti-LRRC8A antibody. The quantification of the band intensity showed an about five-fold higher level of the mouse construct. **d**, VRAC current response to the osmotic swelling of HEK 293 cells in Δsalt buffers. Currents were recorded in absence (orange, n=11, p<0.0001) and presence of Sb4 in the pipette solution (violet, n=4, p=0.036) at indicated time points. The difference between activating responses of mouse and human channels is statistically significant (p=0.044). Traces, left, show the mean of 10 (VRAC) and 4 (VRAC+Sb4) representative recordings. **b**, **d**, **i** and **h** refer to time points for current values displayed in bar graphs. Differences were evaluated by two-tailed unpaired *t* testing with Welch's correction applied. **e-h**, Representative current-voltage (I-V) relationships of the response of $LRRC8^{B,C,E-/-}$ (**e**, **f**) and HEK293 (**g**, **h**) cells in presence and absence of Sb4. Raw current traces are shown left. **i**, Activation of LRRC8A/D currents used to probe the inhibitory effect of DCPIB (shown in Fig. 1d). **b**, **d**, **l**, Current densities display values at 100 mV recorded with the ramp protocol shown in **a**. The perfusion with buffers of distinct composition is indicated. Errors are s.e.m..

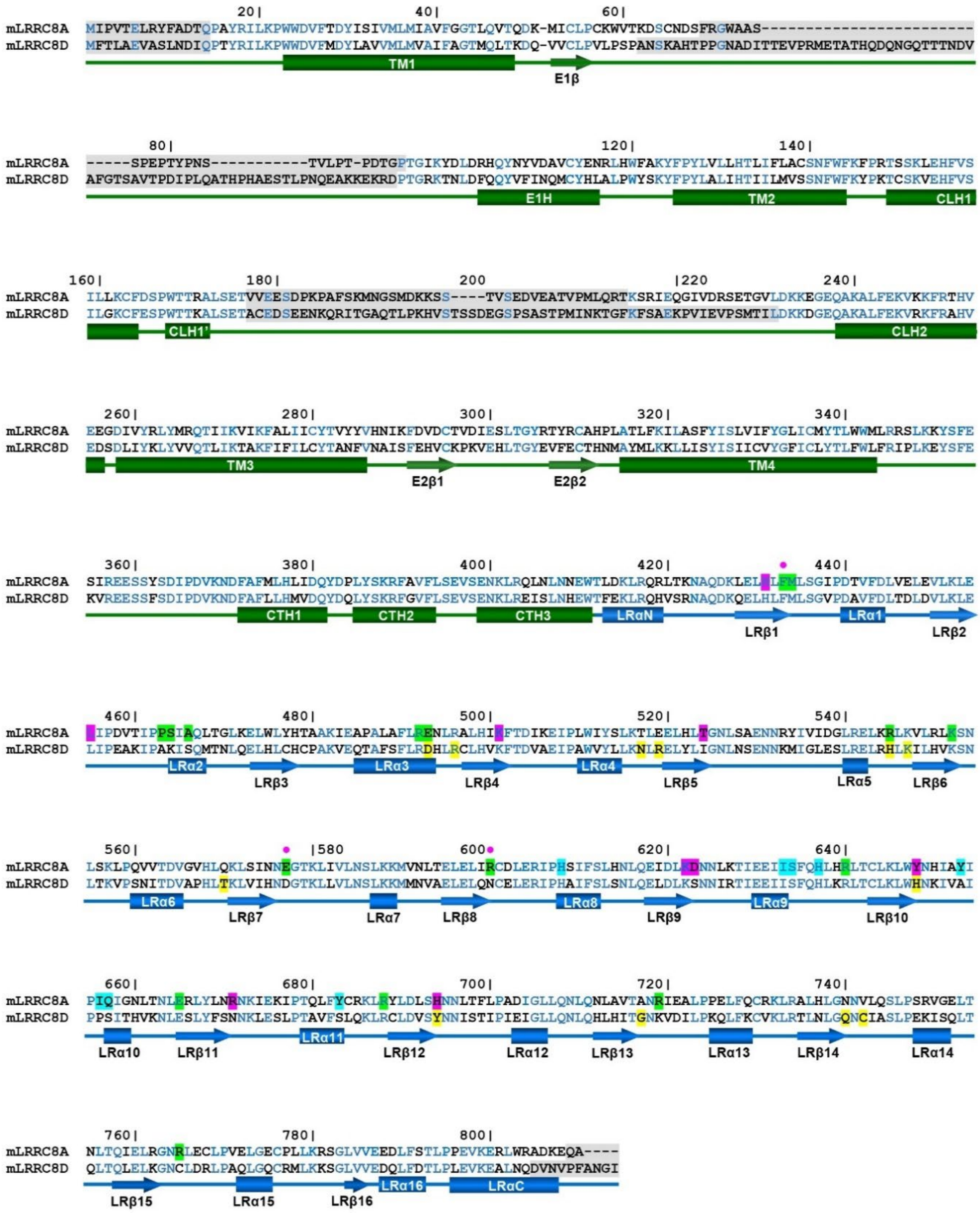

Supplementary Fig. 2: Sequence alignment of murine LRRC8A and D. Sequences of mouse LRRC8A (UniProt ID Q80WG5) and LRRC8D (UniProt ID Q3TI70) are shown with numbers of LRRC8A subunits indicated above and secondary structure elements that are representative for both subunits below. Residues that are not defined in the structure are highlighted in grey. Residues buried in interaction interfaces of the LRRD are highlighted in distinct colors: green, tight interface between LRRDs of LRRC8A, yellow, interface between LRRDs of LRRC8D, cyan, interface with Sb1, magenta, interface with Sb4. Residues interacting with Sb4 that are also part of the tight interface are indicated by dot. Residues that are not defined in the density are highlighted in grey.

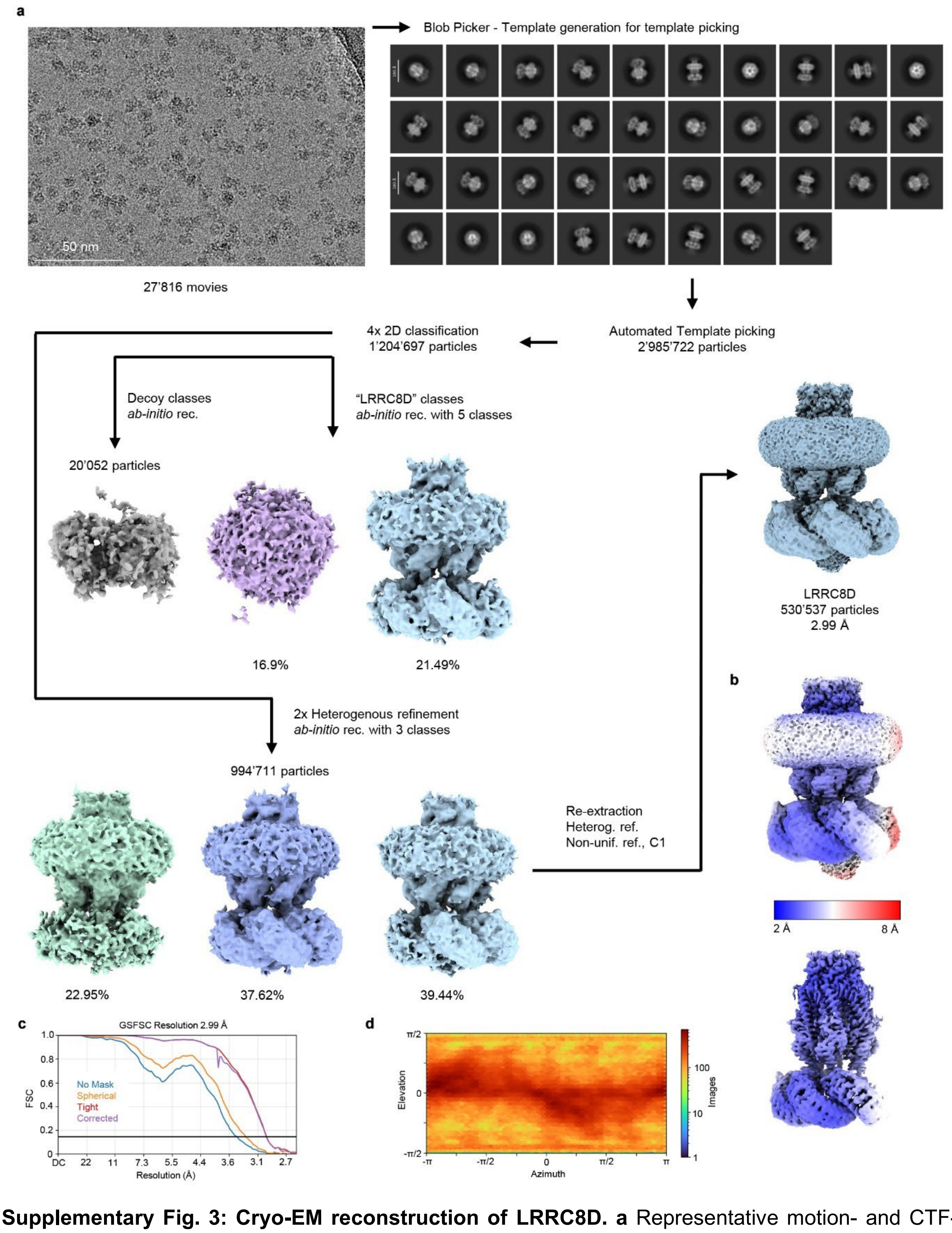

Supplementary Fig. 3: Cryo-EM reconstruction of LRRC8D. a Representative motion- and CTF-corrected micrograph (of a total of 27'816) of the mouse full-length LRRC8D homomer and representation of data processing workflow. The initial particle set was picked using Blob picker with a diameter between 100 and 250 Å, extracted and subjected to 2D classification. Generated 2D class averages were used as templates for automated particle picking. Particles were subjected to four additional rounds of 2D classification. 2D classes presenting clear density of LRRC8D were used for initial ab-initio reconstruction. Two classes with density resembling the protein structure and a single

class lacking respective features (decoy class) were selected for two rounds of heterogenous refinement. After re-extraction, another round of heterogenous refinement, followed by non-uniform refinement with C1 symmetry applied yielded the final map of LRRC8D at 2.99 Å. **b, c,** LRRC8D density at high and low contour level colored according to local resolution. **d** Fourier shell correlation (FSC) plot of the final LRRC8D map. Straight line corresponds to resolution at which the FSC drops below the 0.143 threshold. **e** Heat-map displaying the angular distribution of LRRC8D particles.

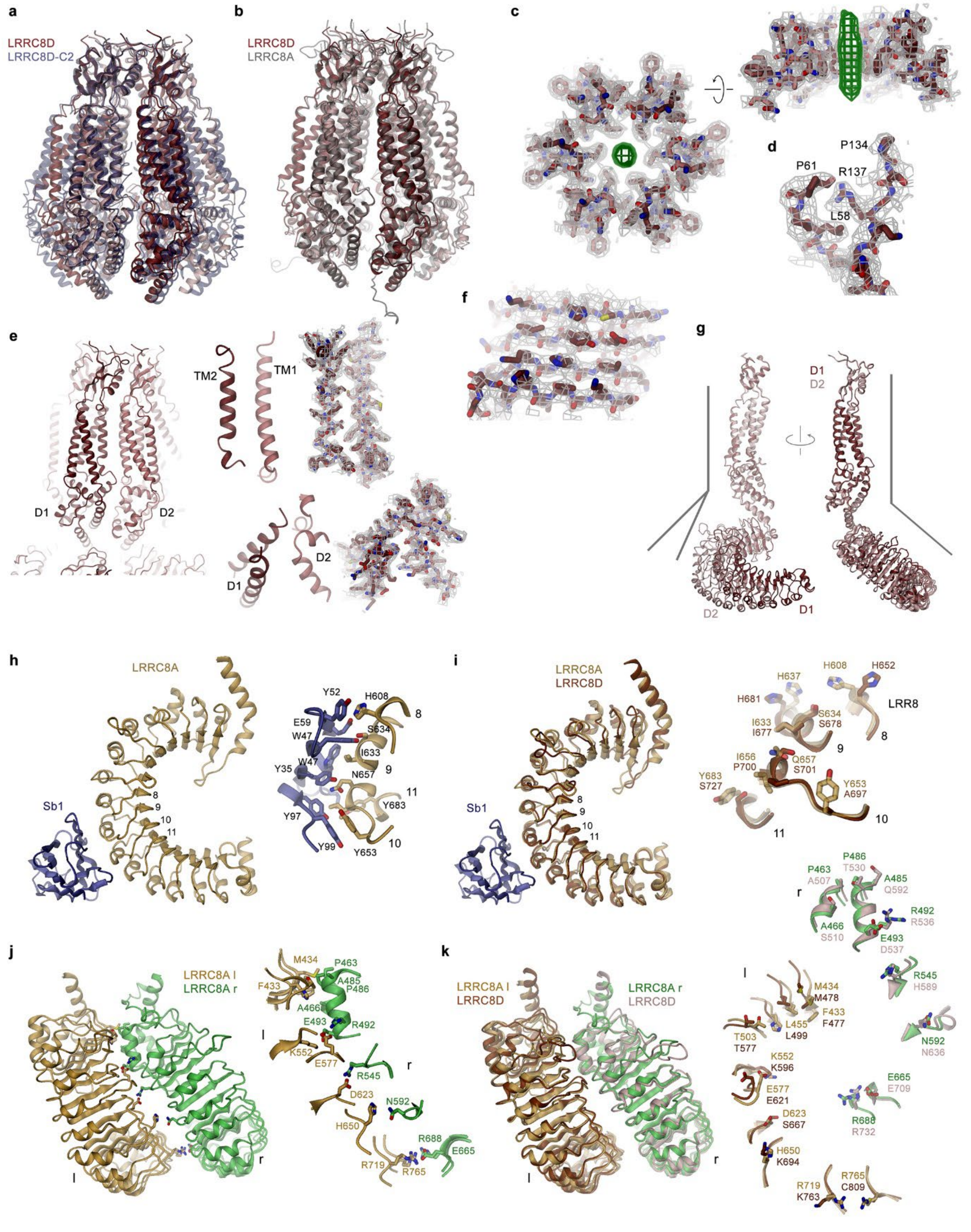

Supplementary Fig. 4: Structural Features of LRRC8D. a, **b,** Superposition of the PDs of the mouse LRRC8D structure determined in this study with, **a**, a previously determined structure of human LRRC8D (PDBID: 6M04, RMSD 4.2 Å) showing a C2-symmetric arrangement of subunits and **b**, the mouse LRRC8A/Sb1 complex (PDB: 7P5V, RMSD 1.7 Å). **c**, Density of the selectivity filter contained in the ISD. Residual density in the pore is highlighted in green. The view is from the extracellular side

(left) and from within the membrane (right). **d**, Density of the extracellular loop region connecting TM1 and TM2. **e**, Subunit interface in the TM and ISD region. Shown is a ribbon representation of the PD (left) and blow-up of the interaction between TM2 and TM1 of adjacent subunits (center) as well as an all-atom representation of the same region (right). **f**, Representative region of the LRRD of the subunit in D1 position. **c-f,** Density (at an overall resolution of 3.0 Å, contoured at 10σ) is shown superimposed on the refined model. **g**, Superposition of the pore domains of the D1 and D2 subunits of the mouse LRRC8D structure shows a similar inclination of the LRRDs with respect to the membrane plane but different tilts from the symmetry axis with the domain in the D2 position having further retracted from the pore. **h**, Interaction interface between LRRC8A and Sb1. Left, ribbon representation of the LRRD of the A subunit in complex with Sb1 as defined in the LRRC8A/Sb1 structure (PDBID: 7P5V). Inset (right) shows blow-up of the interface with interacting residues displayed as sticks. **i**, Comparison of the region mediating Sb1 interactions between LRRC8A and LRRC8D. Superposition of the LRRD of LRRC8D with the LRRD of LRRC8A in complex with Sb1. Inset shows blow-up of residues involved in Sb1 interactions in LRRC8A and their equivalents in LRRC8D. Whereas the residues on the surface of leucine-rich repeats 8 and 9 involved in interactions with CDR1 and 2 of Sb1 are largely conserved between both LRRDs, residues on repeats 10 and 11, which interact with CDR3, show pronounced differences. **j**, Interactions between LRRDs in the tight interface of the LRRC8A homomer with sidechains of interacting residues shown as sticks. Inset (right) shows blow-up of the interaction region. The extended interactions in LRRC8A are hydrophobic between the first repeats, whereas they mainly involve charged residues further downstream. **k**, Superposition of the LRRD of LRRC8D on tightly interacting LRRC8A subunits (left) and blow-up of the interaction region of residues in the l and r position viewed from the interface. While several of the interacting residues in the LRRC8A interface are altered in LRRC8D, their polarity and net charge is generally conserved. Consequently, direct domain interactions within LRRDs of LRRC8D might have been weakened while the overall surface properties of the domain are preserved.

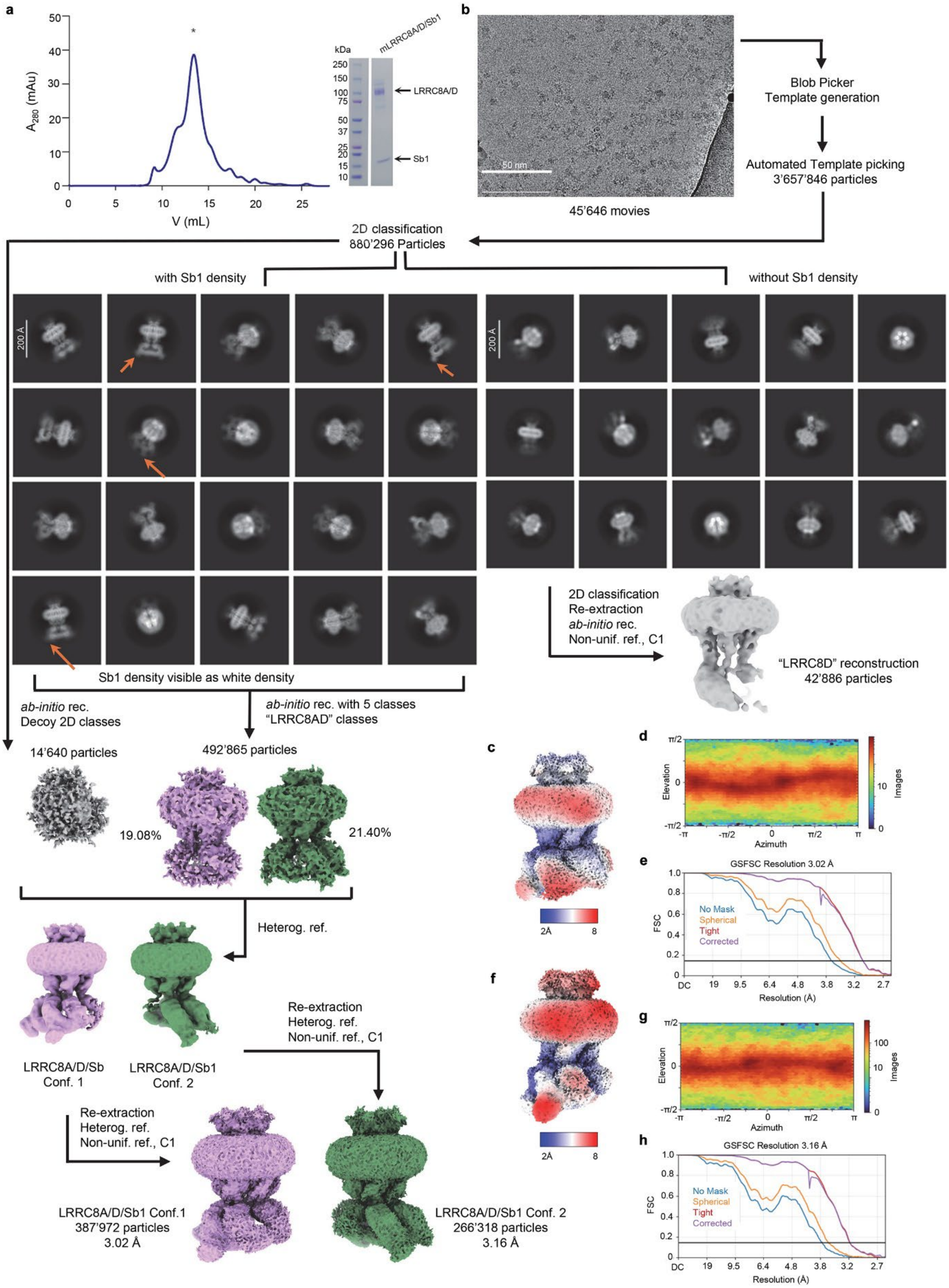

Supplementary Fig. 5: Cryo-EM reconstruction of the LRRC8A/D/Sb1 complex. a, Size exclusion chromatography trace of the sample preparation of the LRRC8A/D complex used for structure determination. An SDS PAGE gel of the peak fraction (*) is shown right. The sample was obtained by affinity purification of tagged D subunits and contains LRRC8A/D heteromers and a smaller fraction of

LRRC8D homomers. **b**, Representative of (a total of 45’646) motion- and CTF-corrected micrographs of the mouse full-length LRRC8A/D heteromer in complex with Sb1 and a summary of the data processing workflow. An initial particle set was picked using blob picker with a minimum particle distance of 120 Å, extracted and subjected to 2D classification. Generated 2D class averages were used as templates for automated particle picking. Particles were subjected into a second round of 2D classification. 2D classes presenting clear density of LRRC8A/D with Sb1 bound in the LRR domain were used for an initial ab-initio reconstruction. Classes without density of Sb1 were assumed to represent LRRC8D homomers and were processed separately. For the LRRC8A/D/Sb1 complex, two classes with clear density resembling the protein and a single class lacking respective features (decoy class) were selected for two rounds of heterogenous refinement. After re-extraction, another round of heterogenous refinement, followed by non-uniform refinement with C1 symmetry applied, yielded final maps of the LRRC8A/D/Sb1 complex in conformation 1 at 3.02 Å and conformation 2 at 3.16 Å resolution. **c,** LRRC8A/D/Sb1 in conformation 1 colored according to local resolution. **d,** Heat-map displaying the angular distribution of particles of the LRRC8A/D in complex with Sb1 in conformation 1. **e,** Fourier shell correlation (FSC) plot of the final maps of the LRRC8A/D/Sb1 complex in conformation 1. **f**, LRRC8A/D/Sb1 in conformation 2 colored according to local resolution. **g**, Heat-map displaying the angular distribution of particles of the LRRC8A/D in complex with Sb1 in conformation 2. **h**, Fourier shell correlation (FSC) plot of the final maps of the LRRC8A/D/Sb1 complex in conformation 2. **e**, **h**, Straight line corresponds to the resolution at which the FSC drops below the 0.143 threshold.

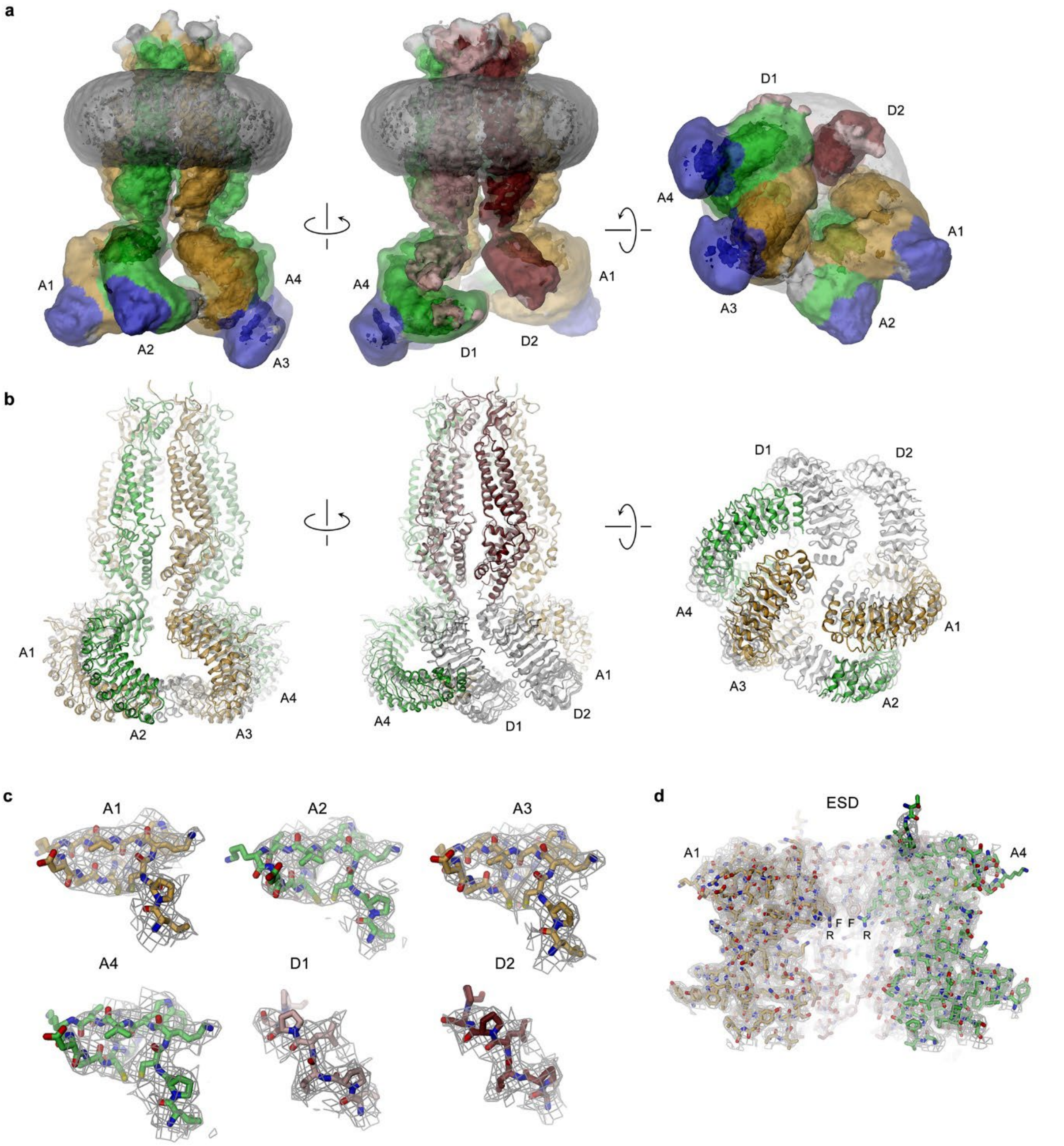

Supplementary Fig. 6: Structural Features of the LRRC8A/D/Sb1 complex. a, Cryo-EM density of conformation 2 (c2) from a dataset of the heteromeric LRRC8A/D/Sb1 complex at 3.2 Å (contoured at 10σ) in indicated orientations. A blurred map at lower contour (b=100, 5σ) is superimposed to also show the envelope of regions with weaker density. Density corresponding to bound sybodies is indicated in blue. **b**, Ribbon representation of the LRRC8A/D/Sb1^{c2} complex with subunit positions labeled. **c**, Density of the extracellular loop connecting TM1 and 2 in different subunits of the LRRC8A/D/Sb1^{c1} complex facilitating subunit assignment. **d**, View of the ESD with two front subunits removed and residues at the pore constriction indicated. **c**, **d**, Cryo-EM density (contoured at 10σ) is shown superimposed on the refined model.

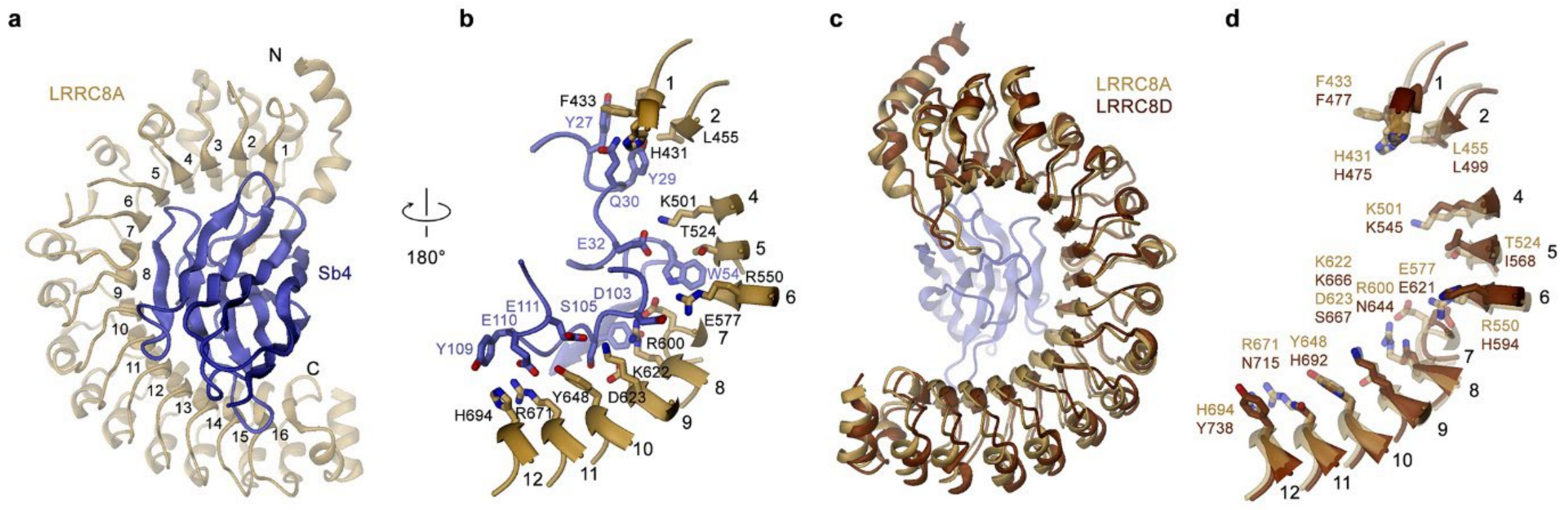

Supplementary Fig. 7 | X-ray Structure the LRRD of LRRC8A in complex with Sb4. a, Ribbon representation of the X-ray structure of the isolated LRRD of LRRC8A in complex with Sb4. **b**, Blow-up of the interaction interface with selected residues shown as sticks. **c**, Superposition of the LRRDs of LRRC8D and the LRRC8A LRRD/Sb4 complex. **d**, Blow-up of the Sb4 interaction region in LRRC8A and the equivalent region in LRRC8D with equivalent residues shown as sticks.

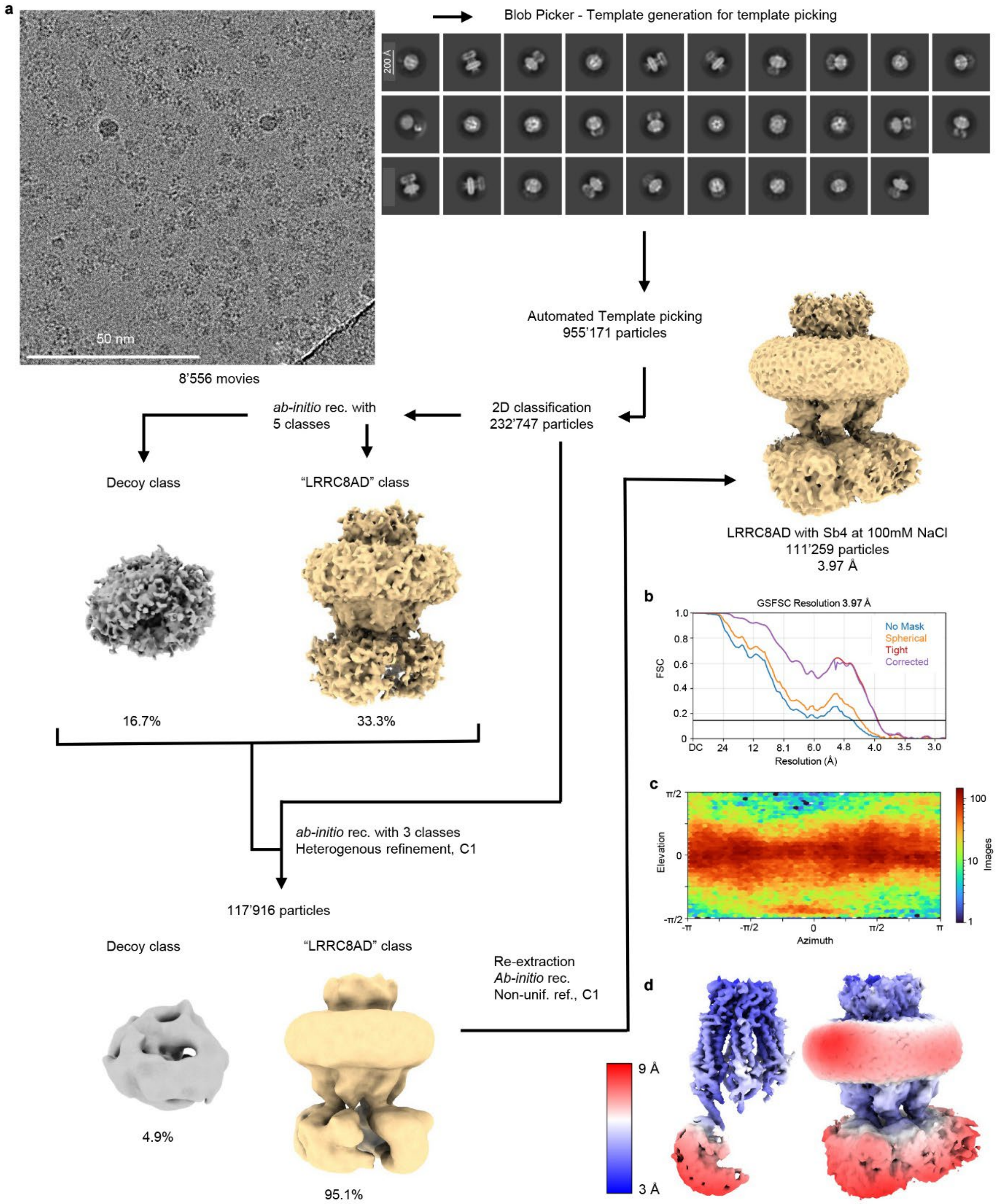

Supplementary Fig. 8: Cryo-EM reconstruction of the LRRC8A/D/Sb4 complex. a Representative of (a total of 8'556) motion- and CTF-corrected micrograph of the mouse full-length LRRC8A/D heteromer in complex with Sb4 and scheme of the data processing workflow. The initial particle set was picked using blob picker with a diameter between 120-250 Å, extracted and subjected to 2D classification. Generated 2D class averages were used as templates for automated particle picking. Particles were subjected into a second round of 2D classification. 2D classes representing protein density were used for an initial ab-initio reconstruction. Two classes with clear density showing features of the channel structure and a single class lacking respective features (decoy class) were selected for

ab-initio reconstructions and three rounds of heterogenous refinement. After re-extraction, particles were submitted to an ab-initio reconstruction followed by a non-uniform refinement with C1 symmetry applied, yielding in a final map of LRRC8A/D/Sb4 at 3.97 Å resolution. **b**, Fourier shell correlation (FSC) plot of the final map of LRRC8A/D in complex with Sb4. Straight line corresponds to the resolution at which the FSC drops below the 0.143 threshold. **c**, Heat-map displaying the angular particle distribution of LRRC8A/D in complex with Sb4. **d**, Final map of LRRC8A/D in complex with Sb4 at high (left) and low contour level (right) colored according to local resolution.

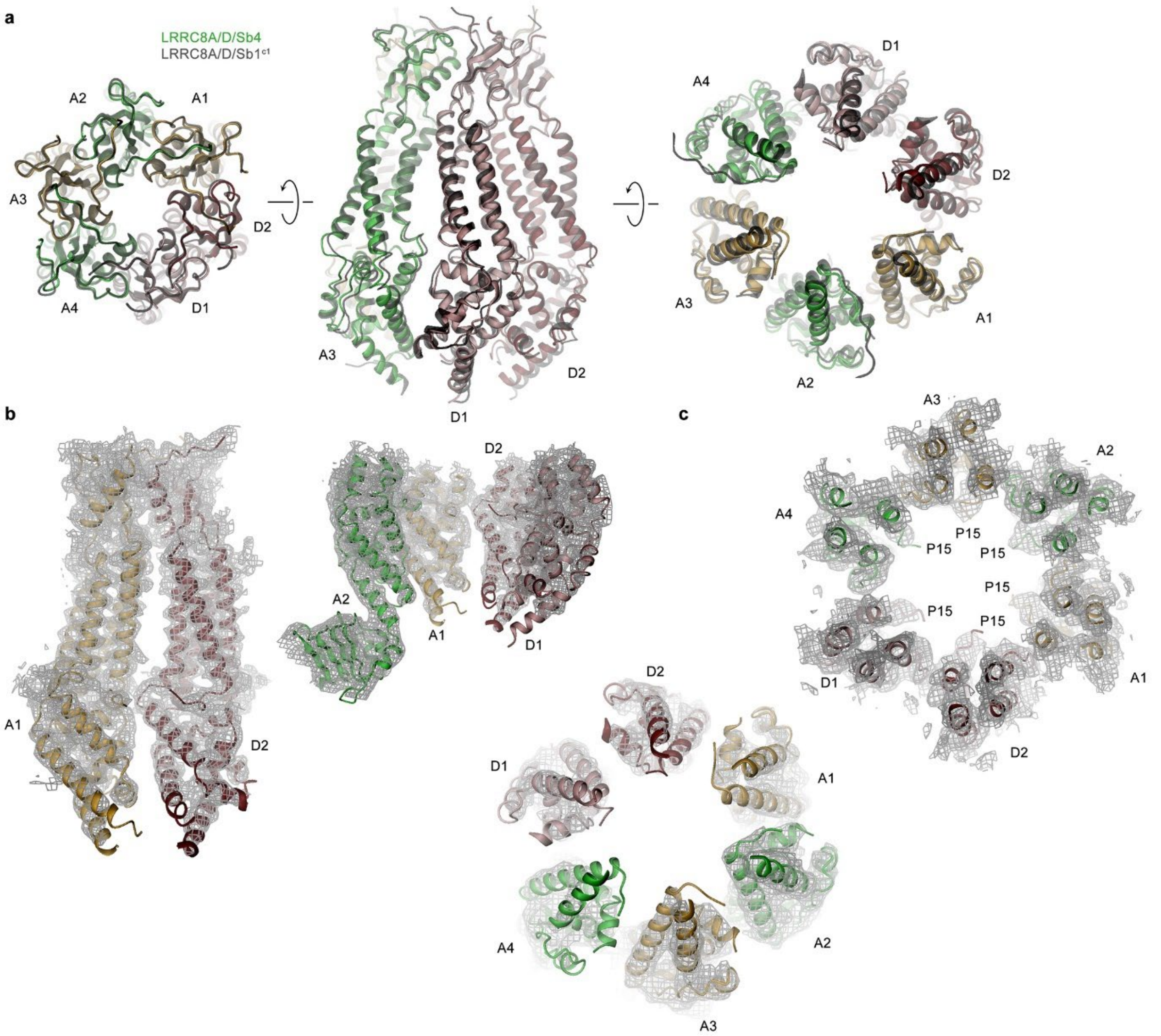

Supplementary Fig. 9: Structural Features of the LRRC8A/D/Sb4 complex. a, Superposition of the PDs of the LRRC8A/D/Sb1c1 and the LRRC8A/D/Sb4 complexes (RMSD: 1.3 Å) in indicated orientations. **b**, Properties of the PD of the LRRC8A/D/Sb4 complex. Left, PDs of interacting subunits. Right, ISD region of three interacting subunits. The subunit assignment is tentative. **c**, View of the PDs of the hexameric channel from the cytoplasmic side (bottom), and view of the pore region surrounding the N-termini from the extracellular side (top). The first residue of the N-terminal region that is defined in the density is labelled. **b**, **c**, Cryo-EM density (contoured at 3σ) is shown superimposed on a ribbon of the model.

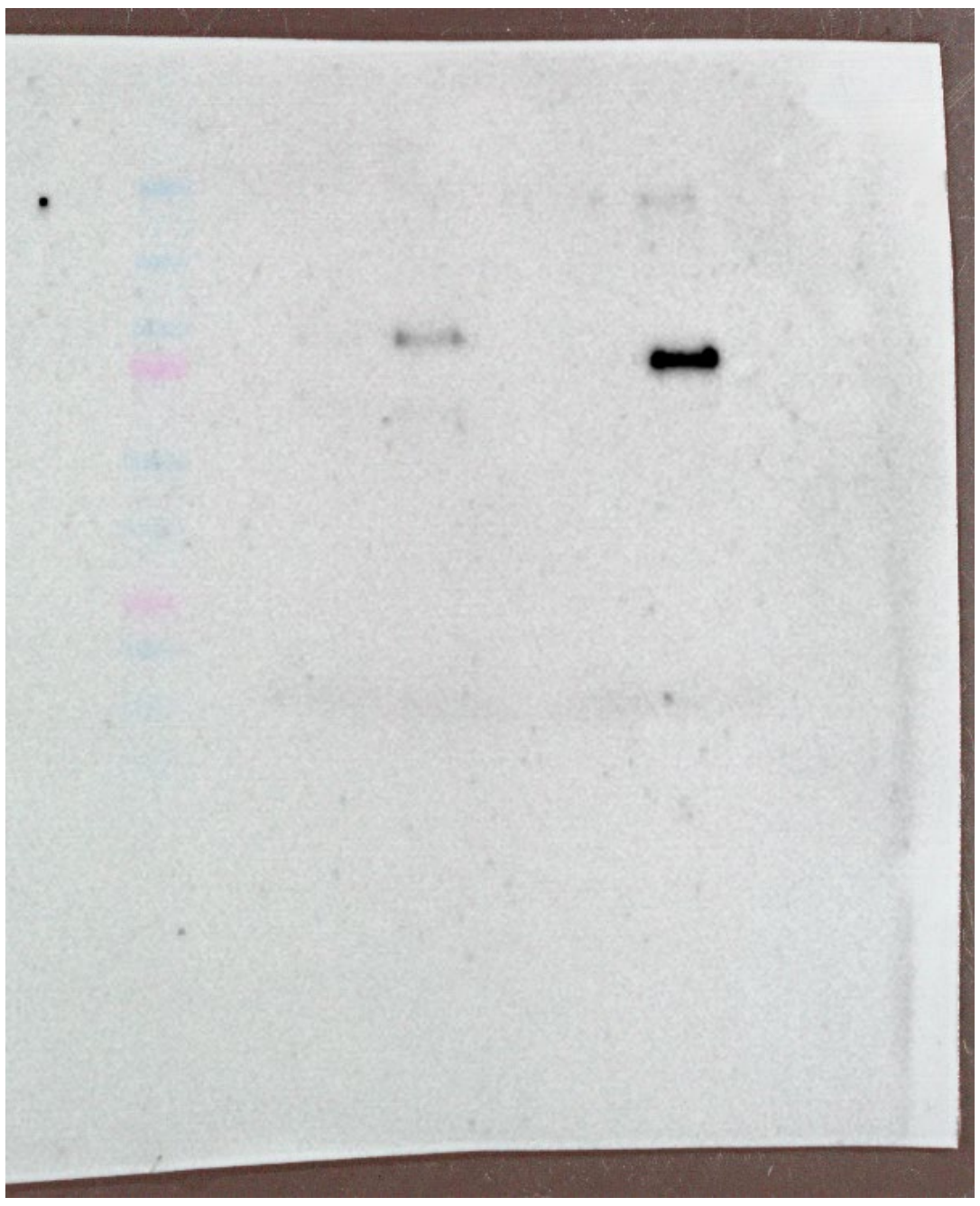
Western Blot for Supplementary Fig. 1c

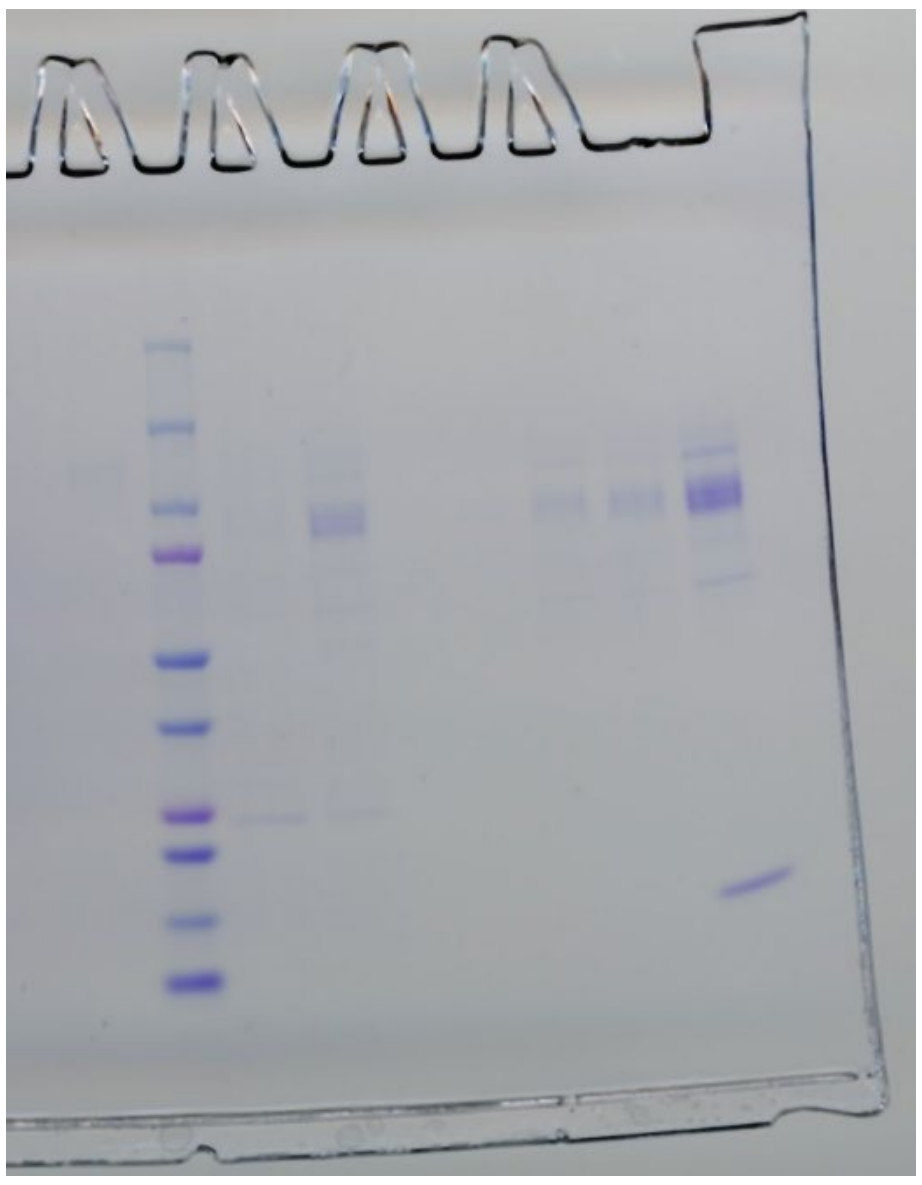
SDS-Page gel for Supplementary Fig. 5a
