# Supplementary material for: Structures of the volume-regulated anion channel LRRC8A/D in activating and inhibiting conditions
Source: Nat Commun. 2026 Sep 17;17:9884. doi: 10.1038/s41467-026-77508-x (PMC13586245; doi:10.1038/s41467-026-77508-x)
Supplement: Supplementary file 2 [file 41467_2026_77508_MOESM2_ESM.pdf]

nature portfolio

Corresponding author(s): Raimund Dutzler

Last updated by author(s): Jul 28, 2026

Reporting Summary

Nature Portfolio wishes to improve the reproducibility of the work that we publish. This form provides structure for consistency and transparency in reporting. For further information on Nature Portfolio policies, see our Editorial Policies and the Editorial Policy Checklist.

Statistics

For all statistical analyses, confirm that the following items are present in the figure legend, table legend, main text, or Methods section.

n/a	Confirmed	
☐	☒	The exact sample size (*n*) for each experimental group/condition, given as a discrete number and unit of measurement
☐	☒	A statement on whether measurements were taken from distinct samples or whether the same sample was measured repeatedly
☐	☒	The statistical test(s) used AND whether they are one- or two-sided<br>*Only common tests should be described solely by name; describe more complex techniques in the Methods section.*
☒	☐	A description of all covariates tested
☒	☐	A description of any assumptions or corrections, such as tests of normality and adjustment for multiple comparisons
☐	☒	A full description of the statistical parameters including central tendency (e.g. means) or other basic estimates (e.g. regression coefficient) AND variation (e.g. standard deviation) or associated estimates of uncertainty (e.g. confidence intervals)
☐	☒	For null hypothesis testing, the test statistic (e.g. *F*, *t*, *r*) with confidence intervals, effect sizes, degrees of freedom and *P* value noted<br>*Give P values as exact values whenever suitable.*
☒	☐	For Bayesian analysis, information on the choice of priors and Markov chain Monte Carlo settings
☒	☐	For hierarchical and complex designs, identification of the appropriate level for tests and full reporting of outcomes
☒	☐	Estimates of effect sizes (e.g. Cohen's *d*, Pearson's *r*), indicating how they were calculated

Our web collection on statistics for biologists contains articles on many of the points above.

Software and code

Policy information about availability of computer code

Data collection	EPU v2.9-3.9, Clampex v10.7 and v11.7, Dectris, Viber Fusion FX7 imaging system
Data analysis	Excel 2019, CryoSPARC v4, Phenix v1.20.1, Coot v0.9.8, DINO v0.9.6, Chimera 1.16, ChimeraX 1.10, GraphPad Prism v10.5, Clampfit v10.7 and v11.7, Fiji v1.54pi, XDS, Phaser

For manuscripts utilizing custom algorithms or software that are central to the research but not yet described in published literature, software must be made available to editors and reviewers. We strongly encourage code deposition in a community repository (e.g. GitHub). See the Nature Portfolio guidelines for submitting code & software for further information.

Data

Policy information about availability of data

All manuscripts must include a data availability statement. This statement should provide the following information, where applicable:

- Accession codes, unique identifiers, or web links for publicly available datasets
- A description of any restrictions on data availability
- For clinical datasets or third party data, please ensure that the statement adheres to our policy

The three-dimensional cryo-EM density maps have been deposited in the Electron Microscopy Data Bank under accession numbers EMD-55589 (LRRC8D), EMD-55590 (LRRC8A/D/Sb1 c1), EMD-55591 (LRRC8A/D/Sb1 c2), EMD-55592 (LRRC8A/D/Sb4) The deposition includes maps of full-length proteins and corresponding both half-maps. Coordinates have been deposited in the Protein Data Bank under accession numbers 9T5M (LRRC8D), 9T5N (LRRC8A/D/Sb1 c1),

9T5O (LRRC8A/D/Sb1 c2) and 9T5P (LRRC8A/D/Sb4). Coordinates and structure factors of the X-ray structure of the LRRD of LRRC8D and LRRD of LRRC8A in complex with Sb4 have been deposited in the PDB under accession number 9T5Q and 9T5R, respectively.

Research involving human participants, their data, or biological material

Policy information about studies with human participants or human data. See also policy information about sex, gender (identity/presentation), and sexual orientation and race, ethnicity and racism.

Reporting on sex and gender	N/A
Reporting on race, ethnicity, or other socially relevant groupings	N/A
Population characteristics	N/A
Recruitment	N/A
Ethics oversight	N/A

Note that full information on the approval of the study protocol must also be provided in the manuscript.

Field-specific reporting

Please select the one below that is the best fit for your research. If you are not sure, read the appropriate sections before making your selection.

☒ Life sciences ☐ Behavioural & social sciences ☐ Ecological, evolutionary & environmental sciences

For a reference copy of the document with all sections, see nature.com/documents/nr-reporting-summary-flat.pdf

Life sciences study design

All studies must disclose on these points even when the disclosure is negative.

Sample size	Sample size was not calculated. Functional experiments were performed multiple times with similar results observed. Sample size was determined, when the addition of further data points did not change the conclusions of the study. Complete cryoEM and Xray statistics are provided in Supplementary Table 2 and 3.
Data exclusions	While the electrophysiology experiments were performed multiple time with similar results, only leaky recordings were removed from the final data set.
Replication	To ensure reproducibility of the experimental findings, electrophysiological recordings were performed multiple times at different cell passage numbers and from different transfections. Cells with leaky currents were excluded from the analysis (as described under data exclusions), remaining data set was presented as a total. Final set of electrophysiology data is presented as the mean of the indicated number of biological replicates, respective errors are indicated.
Randomization	Randomization was not applicable to this study, as it was conducted entirely in-vitro under standardized experimental conditions.
Blinding	Blinding was not applied as this is deemed not practically feasible.

Reporting for specific materials, systems and methods

We require information from authors about some types of materials, experimental systems and methods used in many studies. Here, indicate whether each material, system or method listed is relevant to your study. If you are not sure if a list item applies to your research, read the appropriate section before selecting a response.

Materials & experimental systems

n/a	Involved in the study	
☐	☒	Antibodies
☐	☒	Eukaryotic cell lines
☒	☐	Palaeontology and archaeology
☒	☐	Animals and other organisms
☒	☐	Clinical data
☒	☐	Dual use research of concern
☒	☐	Plants

Methods

n/a	Involved in the study	
☒	☐	ChIP-seq
☒	☐	Flow cytometry
☒	☐	MRI-based neuroimaging

Antibodies

Antibodies used	The primary antibody used in this study (Mouse anti-LRRC8A IgG) and secondary antibody)Peroxidase-conjugated AffiniPure Goat Anti-Mouse IgG) are commerically available (Sigma, SAB1412855, clone 8H9; Jackson Immuno Research cat #115035003).
Validation	Validation report of the primary Mouse anti-LRRC8A antibody (Sigma, Cat# SAB1412855, Lot# K9161-8H9) are provided by the supplier. The antibody specifically reacts with the C terminal region of LRRC8A. The antibody was verified by Western Blot using purified LRRC8A protein (Deneka D. et al., Allosteric modulation of LRRC8 channels by targeting their cytoplasmic domains. Nat. Commun., doi: 10.1038/s41467-021-25742-w (2021)). The antibody was used in a 1:1000 dilution. Validation data for the Peroxidase-conjugated AffiniPure Goat Anti-Mouse IgG (H+L) (Jackson ImmunoResearch, Cat# 115-035-003, Lot# 131'600) are provided by the manufacturer. The antibody is affinity-purified and specifically reacts with mouse IgG (heavy and light chains). It is widely used and validated for Western blot applications. Lot-specific information is available on the product page. The antibody was used in 1:10'000 dilution.

Eukaryotic cell lines

Policy information about cell lines and Sex and Gender in Research

Cell line source(s)	HEK293 GnTI- cells were obtained from ATCC (CRL-3022); HEK293LRRC8-/ and HEK293LRRC8B,C,E-/- were generously provided by Prof. Thomas Jentsch
Authentication	HEK293 GnTI- - authenticated by the manufacturer via STR profiling.<br>HEK293 LRRC8-/- and HEK293B,C,E-/- - Expression or lack thereof was confirmed by electrophysiology experiments and Western blots with anti-LRRC8A antibody.
Mycoplasma contamination	The cell lines were tested and are free from mycoplasma contamination
Commonly misidentified lines (See ICLAC register)	No commonly misidentified lines were used in the study

Plants

Seed stocks	N/A
Novel plant genotypes	*Describe the methods by which all novel plant genotypes were produced. This includes those generated by transgenic approaches, gene editing, chemical/radiation-based mutagenesis and hybridization. For transgenic lines, describe the transformation method, the number of independent lines analyzed and the generation upon which experiments were performed. For gene-edited lines, describe the editor used, the endogenous sequence targeted for editing, the targeting guide RNA sequence (if applicable) and how the editor was applied.*
Authentication	*Describe any authentication procedures for each seed stock used or novel genotype generated. Describe any experiments used to assess the effect of a mutation and, where applicable, how potential secondary effects (e.g. second site T-DNA insertions, mosiacism, off-target gene editing) were examined.*
