# Supplementary material for: Structures of the volume-regulated anion channel LRRC8A/D in activating and inhibiting conditions
Source: Nat Commun. 2026 Sep 17;17:9884. doi: 10.1038/s41467-026-77508-x (PMC13586245; doi:10.1038/s41467-026-77508-x)
Supplement: Supplementary file 3 [file 41467_2026_77508_MOESM3_ESM.pdf]

nature portfolio

Peer Review File

Structures of the volume-regulated anion channel LRRC8A/D in activating and inhibiting conditions

Corresponding Author: Professor Raimund Dutzler

This file contains all reviewer reports in order by version, followed by all author rebuttals in order by version.

Version 0:

Reviewer comments:

Reviewer #1

(Remarks to the Author)
The excellent study by Elena Lehmann et al. provides a clear and compelling structural and functional analysis of heteromeric LRRC8A/D volume-regulated anion channels, which play important roles in diverse physiological processes. The contrasting effects of inhibitory and potentiating sybodies elegantly link movements of the cytoplasmic LRR domains to channel activation. The structural data are of high quality; however, additional electrophysiological data are required to fully support the conclusions prior to publication.
Major:
1. In Fig. 1a, the traces appear atypical for VRAC. The baseline increased at a similar rate under isotonic conditions as during hypotonic perfusion, and the currents decreased only modestly upon isotonic reperfusion. The authors may wish to replace the panel with higher quality data. In addition, because the currents did not reach a plateau, the method used to quantify maximal currents in the bar graph (and other similar data) should be stated in the figure legend or methods.
2. There is currently no evidence that endogenous A/D channels from human and mouse differ to such an extent in activity. The pronounced difference observed in Fig. S1a may therefore arise from other factors, including differences in expression levels or trafficking of the overexpressed proteins, which should be addressed with appropriate control experiments (i.e. WB of the total and surface proteins, and IF). Alternatively, the authors may wish to remove the data of human A/D overexpression. In addition, the rectification observed in Fig. S1b and 1d appears excessively strong, even for A/D channels. This is likely attributable to the low chloride concentration in the internal solution. To avoid potential confusion, the authors may consider removing these data and the associated results, as appropriate controls using other VRAC channels under comparable recording conditions are lacking.
3. In Fig. S1c, the endogenous VRAC currents recorded in WT HEK293 cells (typically ~150 pA/pF) appear uncharacteristically small. Do the authors have an explanation for this discrepancy? The unusually low baseline currents make the apparent Sb4-mediated potentiation seem disproportionately dramatic. However, in prior work, the authors presented convincing evidence that Sb4 mainly enhances VRAC currents under isotonic conditions, but not when the channels are fully activated (by intracellular low ionic strength). This apparent inconsistency is confusing (the main paragraph in page 6). If the authors intend to revise or overturn their previous conclusion, it would be important to repeat and directly compare the relevant experiments performed in the earlier study.
4. The authors previously reported that Sb4 can activate LRRC8A homomeric channels. In BCE KO cells, LRRC8A homomers may exist. Therefore, in the presence of Sb4, currents recorded in these cells cannot be unambiguously attributed to LRRC8A/8D heteromeric channels. The effects observed in Fig. 1c may reflect a combined contribution from both LRRC8A/8D and LRRC8A channels, complicating the interpretation of the data. In addition, the authors may consider replacing the control data as suggested in comment #1 and noting this explicitly in the figure legend, as the same dataset appears to be reused. Finally, please also see comment #3. In the authors' previous work, the primary effect of Sb4 was reported on baseline VRAC currents rather than on fully activated currents, which further complicates the interpretation of the results presented here.
5. For Fig. 1d, the recorded currents again cannot be unambiguously attributed to LRRC8A/8D channels, for the same reasons discussed above. In addition, the presentation of this figure is suboptimal and raises several questions. On the left, why are the currents markedly smaller than those shown in Fig. 1c under apparently similar conditions? On the right, what is the rationale for selecting two closely spaced time points (52 and 60 s) for quantification? Overall, the clarity and presentation of Fig. 1d could be improved.
6. So far, there is no evidence that 8A/8D channels are particularly permeable to ATP. In contrary, 8A/8C and 8A/8E channels (but not 8A/8D) appears to be more selective to larger nucleotides (such as cGAMP), which is consistent with their

larger pore (L105 in 8C and 8E vs. F143 in 8D) as reported by the authors and others. Therefore, the basis for the lower part of Fig. 7b and the associated discussion (line 393-399) may not actually exist.
Minor:
1. The authors may consider adding a brief note in the results explaining why the apo structure of LRRC8A/8D was not determined, so that readers can better understand the rationale for directly solving the structures in the presence of the two binders.
2. It would be helpful to clearly indicate which structure is superimposed in Fig. 3b. According to the manuscript, this appears to be the LRRC8A/Sb1 structure, but this is not explicitly stated in either the figure or its legend.
3. In Fig. 3d legend, it is unclear what "the corresponding LRRD domain pair" is.
4. Is it possible to distinguish which LRRD moves up, from LRRC8A or LRRC8D? In Fig. 6b and 7a, it is shown as LRRC8A only. It would be helpful to clarify that in the results.
5. Missing hyphen: volume-regulated anion channel
6. Please check the formatting of reference superscripts in the first page of introduction.
7. Capitalize consistently: “Functional Characterization of LRRC8A/D channels”

Reviewer #2

(Remarks to the Author)
In this paper, Lehmann and colleagues determine structures of the specific A/D subunit-VRAC channel in the presence of activating and inhibiting sybodies targeting the A subunit. They demonstrate that the channel exists in a ratio of A:D, 4:2 as a trimer of dimers, and that the inhibiting sybody Sb1 stabilizes the tight interactions of the A subunits, whereas the activating sybody Sb4 disrupts the interactions amongst the A subunits, leading the movement of the LRRDs and an opening of the channel. Overall, this study provides novel findings, and takes care to appropriately interpret these findings. They authors make sure to not over-interpret their data, and to mention pitfalls and lack of clarity in their data through the paper (for example, they see obvious movement of the LRRDs of LRRC8A with Sb4, but no evidence of gate opening, which would be expected in an activated channel). This study is well done, but there are some specific, largely minor, critiques for the authors to address, which can be found below.
1. The authors demonstrate that mouse LRRC8A/D currents are much larger than those of human LRRC8A/D, which is their reasoning for utilizing mouse LLRC8A/D for the rest of the experiments. However, they also mention that the geometry of the mouse LRRC8D homomer is different from that of humans. While the use of mouse is reasonable, it’s a little hard to follow the overall conclusions. It appears that the rigidity of the LRRDs matters. Does the apparent symmetry impact gating? Flexibility? Given the differences, would you predict the sybodies would act the same on other orthologues (like human).
2. The caption for Figure 1 has some spelling errors and could be more concise.
3. There is an unexplained decrease in the size of the current at the end of the hypotonic condition in Figure 1b before the switch to isotonic. Can the authors please clarify the experimental design so that this decrease is explained and accounted for.
4. The authors should include statistical significance in their Figure 1, as opposed to just within the figure legend to help readers see more clearly.
5. The sentence that ends at line -274 is confusing and should be clarified by the authors.
6. For the electrophysiology methods sections, the authors provide typical pipette resistances and the resistance used to establish whole-cell, but they do not provide their criteria for cells they accepted for analysis (such as access resistance cutoff, membrane resistance cutoff, etc.). The authors should provide a detailed explanation of the requirements for cells utilized in the electrophysiology section.
7. While the authors present their statistical methods in their caption for Figure 1, the paper would benefit from a small data analysis and statistics section in the methods.

Reviewer #3

(Remarks to the Author)
The manuscript by Lehmann and colleagues addresses the gating mechanism of the volume regulated anion VRAC channel LRRC8A/D. Under physiological conditions, a VRAC channel is formed with A subunit(s) together with at least one other type of subunit. Different compositions are responsible for the varied permeation specificity of ion/small molecules. Using cryo-EM, these authors showed that A and D subunits form one type of hexamer with a stoichiometry of 4:2, and they determined the structures of the channel in several states. The pore domain of the channel revealed a selectivity filter with a greater diameter, due to the smaller residues contributed by the D subunits. This explains the higher permeation of this channel type for larger molecules such as amino acids. Using two protein binders, Sb1 and Sb4, that act as inhibitor and activator, respectively, the authors also obtained the structure of LRRC8A/D in inhibited and activated states. The work represents significant progress in the mechanic understanding of this important class of channels. After some improvement on text and figure clarity, the manuscript should certainly be seen by the broad audience of Nature Communications.

Her are some minor comments.

1. About the LRRC8A/D-Sb1 structure. Line 200 says 4, but only 3 are shown in Fig. 3a.

2. I have the same question for LRR8A/D-Sb4 complex, is it only 1 Sb4 bound? If so, is it possible that more Sb4s need to bind simultaneously to yield a fully open channel? Such a case has been observed for the acetylcholine receptor, to which the binding of two ligands is required to open the channel.

3. Based on this and other structures of heteromeric VRACs, is it possible that the same type of subunits forms pairs?

4. The coloring scheme in the structures in Figs 2-7 is not explained clearly. Some A subunits are colored green and other purple without an explanation.

5. For Figs. 4&6, the heights of the top-view cross-sections should be indicated in a side-view figure.

6. In Fig. 5a, a box is needed to indicate the insert.

7. SI Table 1: um -> µm.

8. SI Tables 1&2: many numbers are shown in a German or French style, which needs to be changed.

Reviewer #4

(Remarks to the Author)
In this manuscript, Lehmann and colleagues investigate the structure and function of heteromeric LRRC8A/D volume-regulated anion channels using a combination of patch-clamp electrophysiology, cryo-EM, and X-ray crystallography. The authors present structures of the LRRC8D homomer, LRRC8A/D heteromers bound to the inhibitory sybody Sb1, the isolated LRRD of LRRC8A bound to the potentiating sybody Sb4, and the LRRC8A/D complex in the presence of Sb4. The technical quality of the structural data appears to be high (provided links to download and directly examine maps and models are not functional), and the work represents a substantial experimental effort.

While the dataset itself is strong, I have several fundamental concerns regarding the interpretation, novelty, and functional framing of the results. In particular, the manuscript would benefit from clearer motivation for how these structures advance our understanding of LRRC8A/D channel activation and permeation, improved clarity and consistency in the electrophysiological analysis, and a more cautious interpretation of structural observations in relation to channel “activation” and substrate selectivity.

1. Definition and evidence for channel “activation”

A central concern throughout the manuscript is the definition of activation. In Figure 1a, the authors state that LRRC8B/C/E–/– cells (endogenously expressing A/D) are activated by hypotonic conditions. However, inspection of the current–time traces suggests that the slope under hypotonic conditions closely resembles that observed under isotonic conditions. This makes it difficult to conclude that hypotonic exposure induces a discrete activation event, as opposed to reflecting a slow time-dependent current increase that would also occur if isotonic conditions were simply extended.

In contrast, overexpressed mouse LRRC8A/D channels (Fig. 1b) show a much more robust response to hypotonicity, followed by recovery upon re-exposure to isotonic buffer. This discrepancy makes it difficult to directly compare endogenous and overexpressed systems, and it raises the question of whether the summary data comparing isotonic and hypotonic currents in Fig. 1a accurately reflect activation rather than baseline drift.

A related issue is evident in Supplementary Figure 1a, where human LRRC8A/D channels appear to produce currents close to the noise level. These observations further complicate the interpretation of activation across species and systems. The authors should extend isotonic recordings to the same time point used for hypotonic measurements and to compare these directly, to more convincingly demonstrate hypotonic activation.

2. Clarity and consistency of electrophysiological conditions

The electrophysiological experiments are difficult to interpret due to inconsistent experimental conditions and incomplete reporting. For example, Figure 1b (green trace) and Supplementary Figure 1a both appear to show mouse LRRC8A/D expressed in LRRC8–/– cells, yet the reported current densities differ substantially (~320 pA/pF versus ~120 pA/pF). Based on the manuscript, it is not clear why these datasets differ so markedly, particularly given the similar labeling and apparent experimental setup.

I strongly recommend that the authors include a summary table listing, for each electrophysiological dataset: cell line, species, expression method, bath and pipette solutions, osmotic protocol (Δsalt vs Δmannitol), and mode of sybody delivery. This would greatly improve transparency and reproducibility.

In addition, the manuscript states that activation curves are derived from currents measured at “100 mV, 10 ms after the ramp.” Given the complexity of the voltage protocol, a schematic indicating where this measurement is taken would be extremely helpful. It would also be useful to explain why such an extensive voltage protocol is employed if only a small portion of it is used for quantifying activation, and whether other parts of the protocol reveal additional differences between conditions.

3. Comparison of Sb1 and Sb4 effects

The functional effects of Sb1 and Sb4 are evaluated using markedly different experimental approaches. Sb1 is introduced via intracellular expression, whereas Sb4 is delivered through the patch pipette. In addition, different osmotic paradigms (Δmannitol versus Δsalt) are used across experiments. While each approach may be justified individually, the lack of symmetry makes direct comparison between inhibitory and potentiating effects difficult.

If it is not feasible to standardize these conditions, the manuscript would benefit from a clearer explanation of why different strategies were required and what limitations this imposes on the interpretation of the results. As presented, the variability in experimental design obscures the functional comparison between Sb1- and Sb4-mediated modulation.

4. Biochemical characterization of purified complexes

The manuscript does not include biochemical data (e.g., SDS-PAGE gels or SEC profiles) demonstrating the purity and homogeneity of the LRRC8A/D complexes used for structural studies. This is particularly important given that LRRC8D may form homomeric assemblies (PMID: 32415200) and that only a one-step affinity purifications appear to have been used.

The authors should add this data to assess whether LRRC8D homomers are present in the samples and how effectively heteromeric assemblies were isolated. Including representative purification data would significantly improve confidence in the reproducibility and interpretation of the structural results.

5. Structural interpretation of pore selectivity and activation state

The authors attribute differences in pore-lining residues between A and D subunits to altered substrate selectivity and propose that these changes facilitate permeation of larger anions. However, these claims are based solely on static structural observations. Functional validation through mutagenesis (e.g., Phe-Arg substitutions, alanine scanning, or charge reversals) combined with permeability measurements are necessary to directly support these conclusions.

Related to this, the manuscript repeatedly refers to LRRC8A/D structures as representing an “activated” state. However, despite large rearrangements of the LRRDs—particularly in the presence of Sb4—the pore domain itself shows no detectable conformational changes. This suggests that the captured conformation may represent a primed or pre-activated state rather than an open, conducting channel. I would strongly caution against describing these structures as activated unless this distinction is clearly articulated.

Finally, while the Discussion proposes that Sb4-induced conformations could allow permeation of large anions, the electrophysiological experiments presented only examine chloride currents. No data are shown demonstrating enhanced permeation of larger substrates under Sb4 conditions. These claims should therefore be substantially tempered and additional functional data would be required to challenge this hypothesis

Reviewer #5

(Remarks to the Author)
I co-reviewed this manuscript with one of the reviewers who provided the listed reports. This is part of the Nature Communications initiative to facilitate training in peer review and to provide appropriate recognition for Early Career Researchers who co-review manuscripts.

Reviewer #6

(Remarks to the Author)
I co-reviewed this manuscript with one of the reviewers who provided the listed reports. This is part of the Nature Communications initiative to facilitate training in peer review and to provide appropriate recognition for Early Career Researchers who co-review manuscripts.

Reviewer #7

(Remarks to the Author)
This manuscript describes a study of the function and structure of heteromeric LRRC8A/D channels. The authors demonstrate that, as with homo-oligomeric LRRC8A channels, heteromeric A/D channels are activated by the previously described potentiating Sb4, which binds to the A subunit, and inhibited by Sb1. In order to investigate the mechanism underlying this inhibition and potentiation, the respective structures were determined. These structures reveal the 2:1 (A:D) subunit stoichiometry of the A/D heteromer and suggest that increased flexibility of the cytoplasmic LRRDs is the basis for channel activation. Unfortunately, no changes in the pore domain were observed between the activated and inhibited states, which leaves the mechanistic basis of the conducting state elusive.
The work supports the conclusions and claims. The methodology is sound and meets the standard in the field. The methods allow the work to be reproduced.

I have the following comments:
* the authors determine the structure of homomeric LRRC8D. It is not immediately clear whether this assembly itself is an active form nor whether this specific assembly has any physiological relevance. Please elaborate beyond the rather technical motivation (line 123).

* line 212 > neighboring D subunits. Is this the correct term? One may argue that A1 and A2 are also neighbors to the respective D subunits, yet these are well resolved.
* line 219 > A2 and A3, while A3 and A4 are meant
* line 250 > filter. The manuscript would benefit from detailing the difference in substrate specificity between the LRRC8A homomer and the A/D heteromer (that may be explained by the altered filter) in this section instead of line 332.
* line 268 > the high-resolution structure of the LRRD of LRRC8A in complex with Sb4 does not provide conceptually new information and does has limited impact on the main message of the manuscript. Given that this manuscript is already very heavy on structure figures, I suggest to move this section and Fig. 5 to the supplement.
* LRR8CA/D/Sb4 structure. The authors appear to suggest that a previous LRR8CA/Sb4 structure did not reveal these dynamics in the LRRDs due to the substoichiometric amount of Sb4, whereas for the new structure a molar excess of Sb4 is added. However, the relative amount of Sb4 is only relevant if the respective epitope is accessible. In this respect, please explain the relevance of the lower salt concentration used to obtain the new structure. Are the Sb4 epitopes in all A subunits also accessible without decreasing the salt concentration?
* line 335 > LRRD mobility. The LRRD domains of LRRC8D are mobile in the Sb1-LRRC8A/D structure (Fig. 3) that represents an inhibited state. Please nuance the statement to reflect that activation appears to be correlated with mobility of the LRRD domain of A subunits specifically.
* line 373 > membrane interaction. Comment on the surface properties of the respective LRRD and to what extent that would reconcile with an interaction with the membrane.

Version 1:

Reviewer comments:

Reviewer #1

(Remarks to the Author)
The authors have added significant new data and analysis. I have no other comments except for the question raised about Fig. 6b. Because the whole paper is about A/D channel and A/D is specifically labelled in Fig. 6b, the figure in the current form does imply that A/D channel would conduct ATP. However, the evidence so far suggests that A/C and A/E channels, but not A/D, preferentially conduct larger nucleotides (at least for cGAMP). This is likely due to their different pore residues as discussed previously. I strongly encourage the authors to run their analysis with an experimentally validated A/D substrate such as Blasticidin to make their point validate. This is very important because in addition to the two possibilities the authors discussed, it is also possible that the pore residue sidechain dynamics underlies the (very low) permeability of metabolites as it has been recently suggested for the related large-pore pannexin channel (PMID: 41381453). Based on the new analysis, the authors may want to entertain this third possibility in which the sidechain movement may be enough to accommodate larger substrates than chloride.

Reviewer #2

(Remarks to the Author)
The authors did a good job of addressing reviewer comments and the manuscript is greatly improved.

Reviewer #3

(Remarks to the Author)
The authors have properly addressed all of my concerns regarding the manuscript, and I now recommend its publication.

Reviewer #4

(Remarks to the Author)
The authors have thoroughly addressed the reviewers' comments, and the revised manuscript is substantially improved

Reviewer #5

(Remarks to the Author)
I co-reviewed this manuscript with one of the reviewers who provided the listed reports. This is part of the Nature Communications initiative to facilitate training in peer review and to provide appropriate recognition for Early Career Researchers who co-review manuscripts.

Reviewer #6

(Remarks to the Author)
I co-reviewed this manuscript with one of the reviewers who provided the listed reports. This is part of the Nature Communications initiative to facilitate training in peer review and to provide appropriate recognition for Early Career Researchers who co-review manuscripts.

Reviewer #7

(Remarks to the Author)
The revised version of the manuscript satisfactorily addresses my comments.

Open Access This Peer Review File is licensed under a Creative Commons Attribution 4.0 International License, which permits use, sharing, adaptation, distribution and reproduction in any medium or format, as long as you give appropriate credit to the original author(s) and the source, provide a link to the Creative Commons license, and indicate if changes were made.
In cases where reviewers are anonymous, credit should be given to 'Anonymous Referee' and the source.
The images or other third party material in this Peer Review File are included in the article's Creative Commons license, unless indicated otherwise in a credit line to the material. If material is not included in the article's Creative Commons license and your intended use is not permitted by statutory regulation or exceeds the permitted use, you will need to obtain permission directly from the copyright holder.
To view a copy of this license, visit https://creativecommons.org/licenses/by/4.0/

We thank the reviewers for their constructive comments, which we have considered in our revised manuscript and which we addressed in detail below.

Our revised manuscript contains additional data including

- A novel set of patch-clamp data of LRRC8$^{B/D/E-/-}$ cells that show low but robust currents that activate upon a change to hypotonic conditions.
- Additional patch-clamp recordings for several other conditions.
- An analysis of the content of D homomers in our sample used for the structure determination of the LRRC8A/D/Sb1 heteromer and conclusive evidence that these homomeric channels are efficiently sorted out during 2D classification.
- Data on the surface expression of mouse and human LRRC8A/D channels showing the higher expression of the mouse orthologs.
- We also have also changed the title of our manuscript to 'Structures of the volume regulated channel LRRC8A/D in activating and inhibiting conditions' to better emphasize that we do not claim that the observed structures would necessarily show all features of the annotated conformations.

As several reviewers have commented on the design of the functional investigations by patch clamp electrophysiology, we want to emphasize that the focus of this study is on the elucidation of the organization of heteromeric channels containing of A and D subunits and the characterization of structural features adopted in conditions favoring non-conducting and conducting states of the channel. These were obtained by the binding of synthetic nanobodies that target different regions of the LRR domains of A subunits. In light of this focus, the goal of functional experiments was to demonstrate the distinct effects of sybody binding with one (Sb1) acting as allosteric inhibitor and the second (Sb4) as potentiator of activation. In our recordings, we have selected constructs and conditions that best illustrate the effect of these binders. For the potentiating effect of Sb4, we have used a knockout HEK cell line endogenously expressing only LRRC8A and D. As shown before[1], the current response of these cells is low and we have intentionally applied weakly activating conditions to better illustrate the strong potentiation by Sb4. Conversely, we have used overexpression of the murine orthologs in a HEK cell line where all five LRRC8 genes were knocked out under strongly activating conditions, to illustrate the pronounced inhibitory effect of the co-expressed Sb1. In both cases, the aim was to demonstrate the robust functional effects of the respective sybodies on a qualitative level. While the described data is in close agreement with a previous study[2], they show for the first time the effect of the binders on channels consisting of A and D subunits in response to cell swelling. In our previous study, the effect of the sybodies was demonstrated for endogenous VRACs and overexpressed A subunits at isotonic conditions upon decreasing the cytoplasmic ionic strength, which is an alternative protocol to activate the channels.

In our current study study, we did not aim for a characterization of the detailed substrate selectivity of LRRC8A/D channels as these properties were described previously[1,3]. Our only reference to permeant substrates was made in the discussion, in the context of the description of the extracellular selectivity filter, but these comments are hypothetical. We thus refrained from including additional data relating to substrate selectivity as these investigations go beyond the scope of this study and would require methods other than electrophysiology for a reliable characterization[1]. Instead, we have tried to better emphasize the aim of the functional investigations and the limits of our interpretation of functional and structural data. We hope that in this manner we have addressed the concerns of the reviewers in a satisfactory manner. A detailed response to their comments is provided below.

REVIEWER COMMENTS

Reviewer #1 (Remarks to the Author):

The excellent study by Elena Lehmann et al. provides a clear and compelling structural and functional analysis of heteromeric LRRC8A/D volume-regulated anion channels, which play important roles in diverse physiological processes. The contrasting effects of inhibitory and potentiating sybodies elegantly link movements of the cytoplasmic LRR domains to channel activation. The structural data are of high quality; however, additional electrophysiological data are required to fully support the conclusions prior to publication.

We thank the reviewer for the positive comments. In our revision, we have added additional electrophysiology data to strengthen our main points regarding the effect of respective sybodies on the activation state of the protein. As the main focus in this study was not on the selectivity properties of A/D channels, we have reworded the text to clarify that any references to the selectivity properties of A/D channels were made on the basis of published work[1,3].

Major:

1. In Fig. 1a, the traces appear atypical for VRAC. The baseline increased at a similar rate under isotonic conditions as during hypotonic perfusion, and the currents decreased only modestly upon isotonic reperfusion. The authors may wish to replace the panel with higher quality data. In addition, because the currents did not reach a plateau, the method used to quantify maximal currents in the bar graph (and other similar data) should be stated in the figure legend or methods.

We have now remeasured the current response in $LRRC8^{B,C,E-/-}$ cells in buffers with a larger osmotic gradient under hypotonic conditions (referred to as Δmannitol) and replaced Fig. 1a. The original recordings are still shown in Fig. 1c to illustrate the strong potentiating effect of Sb4 in equivalent conditions (Δsalt). The low current response in $LRRC8^{B,C,E-/-}$ HEK cells is in general agreement with previous studies[1] and might be additionally affected by our recording setup where we locally perfuse the hypotonic conditions on the cells via a theta tube instead of changing the media of the entire outside solution.

2. There is currently no evidence that endogenous A/D channels from human and mouse differ to such an extent in activity. The pronounced difference observed in Fig. S1a may therefore arise from other factors, including differences in expression levels or trafficking of the overexpressed proteins, which should be addressed with appropriate control experiments (i.e. WB of the total and surface proteins, and IF). Alternatively, the authors may wish to remove the data of human A/D overexpression.

Please note that the previous Supplementary Fig. 1a (now Supplementary Fig 1b) shows currents from LRRC8A/D channels that were overexpressed in $LRRC8^{-/-}$ cells. To quantify differences in levels, we have assayed the surface expression of A subunits in cells transfected with murine and human LRRC8A/D constructs and did indeed find a five-fold stronger expression for the murine orthologs, which would at least in part account for the stronger response (Supplementary Fig 1c). We have also remeasured the current response of human AD channels overexpressed in $LRRC8^{-/-}$ cells and updated the recordings shown in Supplementary Fig. 1b.

We have made the following changes to the Text, line 120-146:

'In parallel, we also probed the response after transfection of human and murine constructs in LRRC8-/- cells in the weaker activating Δsalt conditions. While we found a moderate increase of currents for human LRRC8A/D channels, these were pronounced for their mouse orthologs (Supplementary Fig. 1b). The lower response of human channels appears to be both, a consequence of the lower surface expression and the weaker responsiveness of the human ortholog (Supplementary Fig. 1c).

In addition, the rectification observed in Fig. S1b and 1d appears excessively strong, even for A/D channels. This is likely attributable to the low chloride concentration in the internal solution. To avoid potential confusion, the authors may consider removing these data and the associated results, as appropriate controls using other VRAC channels under comparable recording conditions are lacking.

Please note that the I-V relationships displayed in the original Supplementary Fig 1b and d were obtained from representative recordings contributing to the current responses displayed in Fig. 1c (measured 400 sec after switching to hypotonic conditions). While the strong rectification might to some extend be a consequence of the lower intracellular Cl^- concentration, it should with 44 mM be sufficiently high to avoid artifacts. It should also be emphasized that the strong rectification of A/D channels is in agreement with previous work[1]. We have now also provided current traces from WT cells recorded under equivalent conditions, which show a generally similar behavior with somewhat less pronounced rectification properties. These data are displayed as Supplementary Fig. 1g, h.

3. In Fig. S1c, the endogenous VRAC currents recorded in WT HEK293 cells (typically ~150 pA/pF) appear uncharacteristically small. Do the authors have an explanation for this discrepancy? The unusually low baseline currents make the apparent Sb4-mediated potentiation seem disproportionately dramatic. However, in prior work, the authors presented convincing evidence that Sb4 mainly enhances VRAC currents under isotonic conditions, but not when the channels are fully activated (by intracellular low ionic strength). This apparent inconsistency is confusing (the main paragraph in page 6). If the authors intend to revise or overturn their previous conclusion, it would be important to repeat and directly compare the relevant experiments performed in the earlier study.

Please note that we have intentionally recorded WT and LRRC8A/D currents at conditions where the response is weak to better emphasize the effect of Sb4 as potentiator of channel activity.

We have emphasized this in the revised manuscript, line:

The lower response observed here compared to published recordings under equivalent conditions could be a consequence of our measurement setup, where the buffer solutions were applied to cells via a theta tube leading to a local perfusion on the cell instead of a complete exchange of the buffer. We have so far never shown the effect of Sb4 in response to cell swelling, as in previous studies the currents were activated in response to a decrease of the intracellular salt concentration[2], which provides an alternative stimulus that causes channel activation. There is thus no inconsistency to previous experiments.

4. The authors previously reported that Sb4 can activate LRRC8A homomeric channels. In BCE KO cells, LRRC8A homomers may exist. Therefore, in the presence of Sb4, currents recorded in these

cells cannot be unambiguously attributed to LRRC8A/8D heteromeric channels. The effects observed in Fig. 1c may reflect a combined contribution from both LRRC8A/8D and LRRC8A channels, complicating the interpretation of the data.

We have indeed observed the strong potentiation of currents in homomeric LRRC8A channels expressed in LRRC8$^{-/-}$ cells in response to low intracellular ionic strength (i.e. 75 mM NaCl) in a previous study[2], whereas the effect in WT cells was already observed at higher NaCl concentrations. This suggests that the effect of Sb4 would be more pronounced in heteromeric LRRC8 channels, which are more readily activated. We thus expect that the observed currents are dominated by LRRC8A/D heteromers, as also indicated by the characteristic current-voltage relationships and the desensitization at positive voltage, which is a hallmark of channels containing D subunits (Supplementary Fig. 1f). Please also note that the main purpose of this experiment is to emphasize the general ability of Sb4 to potentiate LRRC8A/D currents.

In addition, the authors may consider replacing the control data as suggested in comment #1 and noting this explicitly in the figure legend, as the same dataset appears to be reused.

In our revised manuscript we now show data of LRRC8A/D channels endogenously expressed in LRRC8$^{B,C,E-/-}$ cells in response to Δmannitol conditions in Fig. 1a to illustrate the general activation properties of these cells. Conversely, we show the data recorded in Δsalt conditions in Fig. 1c to illustrate the potentiating effect of Sb4 under the same conditions.

Finally, please also see comment #3. In the authors' previous work, the primary effect of Sb4 was reported on baseline VRAC currents rather than on fully activated currents, which further complicates the interpretation of the results presented here.

As mentioned above, our previous studies of Sb4 were performed with a different activation protocol[2] and do not contradict the results presented here that Sb4 potentiates LRRC8A/D currents. We suspect that Sb4 acts by increasing the open probability of the channel, which likely remains low in absence of the sybody, as reflected in the fact that currents have not reached steady state under the applied conditions.

5. For Fig. 1d, the recorded currents again cannot be unambiguously attributed to LRRC8A/8D

channels, for the same reasons discussed above. In addition, the presentation of this figure is suboptimal and raises several questions. On the left, why are the currents markedly smaller than those shown in Fig. 1c under apparently similar conditions? On the right, what is the rationale for selecting two closely spaced time points (52 and 60 s) for quantification? Overall, the clarity and presentation of Fig. 1d could be improved.

Fig. 1d shows the currents in response to the same activation protocol as shown in Fig. 1c. The full activation protocol is now displayed in Supplementary Fig. 1i where it is also apparent that the currents have not reached steady state. The difference in average current density (60% of the mean currents shown in Fig. 1c) reflects the variability between the recordings of different cells as illustrated by the scattering of data points in the bar graphs. In our revised figure, we now only show the current ratio reached towards the end of the recordings.

6. So far, there is no evidence that 8A/8D channels are particularly permeable to ATP. In contrary, 8A/8C and 8A/8E channels (but not 8A/8D) appears to be more selective to larger nucleotides (such as cGAMP), which is consistent with their larger pore (L105 in 8C and 8E vs. F143 in 8D) as reported by the authors and others. Therefore, the basis for the lower part of Fig. 7b and the associated discussion (line 393-399) may not actually exist.

We did not imply that A/D channels would conduct ATP. The figure is meant to relate the pore size of a hexameric channel composed of LRRC8A and D subunits determined in this study to molecules that have been suggested to permeate endogenous VRAC channels. The aim of this comparison is to define open questions concerning the permeation of larger substrates that on a structural level is still poorly understood. As mentioned above, we do not want to engage in detailed speculations concerning the conduction of nucleotides and want to emphasize that the size of cGAMP would even exceed that of ATP.

We have introduced the following changes in the manuscript, line 434-439:

'While the observed pore geometry would be generally compatible with substrates up to the size of acidic amino acids, the dimensions of larger molecules that were also ascribed as substrates of certain VRACs such as ATP and the dinucleotide cGAMP would exceed the observed pore diameter (Fig. 6b) and either require a strongly altered pore conformation or a larger oligomeric organization as found for homomeric LRRC8C channels.'

Minor:

1. The authors may consider adding a brief note in the results explaining why the apo structure of LRRC8A/8D was not determined, so that readers can better understand the rationale for directly solving the structures in the presence of the two binders.

We decided to determine the LRRC8A/D/Sb1 structure since this allows a more effective identification of A subunits. This step was essential to define the subunit arrangement and separate LRRC8A/D heteromers from the population of LRRC8D homomers present in our sample. Additionally, the addition of Sb1 rigidifies the structure in a non-conducting state of the channel, which allows for a better comparison with the complex with Sb4, which stabilizes a conducting state.

We have introduced the following sentence to the revised manuscript, line 224-228:

'In this dataset, the presence of Sb1 allowed the unambiguous assignment of A subunits and it stabilized a non-conducting state of the protein. During data processing, particles of heteromeric LRRC8A/D containing bound Sb1 were readily identified during 2D classification and 3D reconstruction allowing their separation from particles not containing Sb1 density (Supplementary Fig 5b).'

2. It would be helpful to clearly indicate which structure is superimposed in Fig. 3b. According to the manuscript, this appears to be the LRRC8A/Sb1 structure, but this is not explicitly stated in either the figure or its legend.

We have now clarified that the structure corresponds to the LRRC8A/Sb1 complex.

Line 1165-1166:

The superimposed structure of LRRC8A (PDBID 7P5V) is shown in grey for comparison.

3. In Fig. 3d legend, it is unclear what "the corresponding LRRD domain pair" is.

We have clarified this in the text.

Line 1169-1171:

The superposition of the isolated LRRD domain pair of the D2 and D3 subunits of the LRRC8D homomer demonstrates the preservation of the relative orientation between both domains.

4. Is it possible to distinguish which LRRD moves up, from LRRC8A or LRRC8D? In Fig. 6b and 7a, it is shown as LRRC8A only. It would be helpful to clarify that in the results.

We cannot assign the strongly displaced LRRD with certainty, since the density of Sb4 could not be identified unambiguously at the low resolution of this map. However, we have observed a similar strong LRRD movement in a mutant of the A subunit with strongly activating phenotype described in an accompanying study[4], suggesting that this might be an A subunit.

5. Missing hyphen: volume-regulated anion channel

We have corrected this mistake.

6. Please check the formatting of reference superscripts in the first page of introduction.

We have corrected the references.

7. Capitalize consistently: "Functional Characterization of LRRC8A/D channels"

We have corrected the text.

Reviewer #2 (Remarks to the Author):

In this paper, Lehmann and colleagues determine structures of the specific A/D subunit-VRAC channel in the presence of activating and inhibiting sybodies targeting the A subunit. They demonstrate that the channel exists in a ratio of A:D, 4:2 as a trimer of dimers, and that the inhibiting sybody Sb1 stabilizes the tight interactions of the A subunits, whereas the activating sybody Sb4 disrupts the interactions amongst the A subunits, leading the movement of the LRRDs and an opening of the channel. Overall, this study provides novel findings, and takes care to appropriately interpret these findings. They authors make sure to not over-interpret their data, and to mention pitfalls and lack of clarity in their data through the paper (for example, they see obvious movement of the LRRDs of LRRC8A with Sb4, but no evidence of gate opening, which would be expected in an activated channel). This study is well done, but there are some specific, largely minor, critiques for the authors to address, which can be found below.

We thank the reviewer for the generally supportive comments.

1. The authors demonstrate that mouse LRRC8A/D currents are much larger than those of human LRRC8A/D, which is their reasoning for utilizing mouse LLRC8A/D for the rest of the experiments. However, they also mention that the geometry of the mouse LRRC8D homomer is different from that of humans. While the use of mouse is reasonable, it's a little hard to follow the overall conclusions. It appears that the rigidity of the LRRDs matters. Does the apparent symmetry impact gating? Flexibility? Given the differences, would you predict the sybodies would act the same on other orthologues (like human).

We do not want to make structural conclusions based on the difference in the activation properties of mouse and human homologs as the difference in the observed current density is partly a consequence of the altered expression levels (Supplementary Fig. 1c). Instead, we show that Sb4 enhances the open probability even of the poorly activated human channel (Fig. 1c) and Sb1 efficiently inhibits even the more easily activated murine orthologs (Fig. 1d), underlining the property of the sybodies stabilize distinct states, thereby facilitating the assignment of observed structural features.

With respect to the LRRC8D homomer, we cannot conclude whether the structural differences compared to a previous human protein structure is a consequence of the paralog or the expression system. In any case, the main motivation for the structure determination of LRRC8D homomer was to define the architecture of this subunit at high resolution, to facilitate its placement in the heteromeric protein. Please note that the murine LRRC8D structure determined here is of much higher resolution than the previous structure obtained from the human homolog.

The interaction of the sybodies is confined to epitopes on single LRRDs of the A subunit at sites that are highly conserved. We thus do not expect any differences between human and murine homologues.

2. The caption for Figure 1 has some spelling errors and could be more concise.

We have rewritten the caption accordingly.

3. There is an unexplained decrease in the size of the current at the end of the hypotonic condition in Figure 1b before the switch to isotonic. Can the authors please clarify the experimental design so that this decrease is explained and accounted for.

We occasionally saw a 20% decrease in currents after the interruption of the continuous ramp protocol for mouse LRRC8A/D channels recorded in Δmannitol buffers. The interruption was used for the recording of current-voltage relationships by a step protocol. The protocol is equivalent to equivalent experiments displayed in Fig. 1 and Supplementary Fig. 1, where we did not observe a similar behavior, including recordings of the same overexpressed channels in Δsalt conditions (Supplementary Fig. 1b). We noticed that the current density upon perfusion of mouse LRRC8A/D channels with the stronger activating Δmannitol buffers is on average twice as high as with Δsalt conditions, reflecting the general difference in VRAC response we observe for these buffer conditions. We thus cannot exclude that the observed effect is related to the high current density observed under such conditions. Please note that we have deliberately selected Δmannitol conditions to illustrate the strong inhibitory effect upon co-expression of sybody Sb1. This effect is already observed at a much earlier stage (below 350 sec) and the last part of the recording is merely carried out to illustrate the VRAC-characteristic deactivation of the channel upon return to isotonic conditions. We have now added additional recordings where the current density decrease is less pronounced (Fig. 1b).

4. The authors should include statistical significance in their Figure 1, as opposed to just within the figure legend to help readers see more clearly.

We now display the statistical significance in bar graphs of Fig. 1 and Supplementary Fig. 1.

5. The sentence that ends at line -274 is confusing and should be clarified by the authors.

We have corrected this sentence in our revision.

Line 280-281:

'This structure has provided a detailed view of the ESD harboring the most constricted part of the pore acting as selectivity filter.'

6. For the electrophysiology methods sections, the authors provide typical pipette resistances and the resistance used to establish whole-cell, but they do not provide their criteria for cells they accepted for analysis (such as access resistance cutoff, membrane resistance cutoff, etc.). The authors should provide a detailed explanation of the requirements for cells utilized in the electrophysiology section.

We have now specified the criteria of recordings included in the analysis in the method section. We want to emphasize that we have not discarded any data with low current response under activating conditions, which is also reflected in the bar graphs including all recordings.

We have made the following changes, Line 831:

Access resistance was between 6-13 MΩ.

Line 923-925

Traces were selected based on the criteria described in the electrophysiology section and measurements meeting these criteria were included in the evaluation irrespectively of the magnitude of the current response.

7. While the authors present their statistical methods in their caption for Figure 1, the paper would benefit from a small data analysis and statistics section in the methods.

We have now included a brief statistics section in the methods (line 921-929).

Reviewer #3 (Remarks to the Author):

The manuscript by Lehmann and colleagues addresses the gating mechanism of the volume regulated anion VRAC channel LRRC8A/D. Under physiological conditions, a VRAC channel is formed with A subunit(s) together with at least one other type of subunit. Different compositions are responsible for the varied permeation specificity of ion/small molecules. Using cryo-EM, these authors showed that A and D subunits form one type of hexamer with a stoichiometry of 4:2, and they determined the structures of the channel in several states. The pore domain of the channel revealed a selectivity filter with a greater diameter, due to the smaller residues contributed by the D subuits. This explains the higher permeation of this channel type for larger molecules such as amino acids. Using two protein binders, Sb1 and Sb4, that act as inhibitor and activator, respectively, the authors also obtained the structure of LRRC8A/D in inhibited and activated states. The work represents significant progress in the mechanic understanding of this important class of channels. After some improvement on text and figure clarity, the manuscript should certainly be seen by the broad audience of Nature Communications.

We thank the reviewer for the supportive comments.

Her are some minor comments.

1. About the LRRC8A/D-Sb1 structure. Line 200 says 4, but only 3 are shown in Fig. 3a.

The density of Sb1 in the LRRC8A/Sb1 complex is indicated in blue. The binding to two subunits (A1 and A2) is evident already at high map contour, which illustrates the low structural heterogeneity of both A subunits. The density is less pronounced in the subunit located at position A3 and even weaker in the A4 position, probably due to the increased mobility of both subunits as consequence of the neighboring D subunits. However, the density of bound sybodies is even in this case still visible in a blurred map at lower contour.

2. I have the same question for LRR8A/D-Sb4 complex, is it only 1 Sb4 bound? If so, is it possible that more Sb4s need to bind simultaneously to yield a fully open channel? Such a case has been observed for the acetylcholine receptor, to which the binding of two ligands is required to open the channel.

We indeed assume that all four A subunits in the LRRC8A/D/Sb4 complex are binding Sb4. We have investigated the binding of Sb4 to homomeric LRRC8A at different stoichiometric ratios and found the amount of sybodies eluting with the channel on size exclusion chromatography to double when changing from a sub-stoichiometric LRRC8A:Sb4 ratio (i.e. 2:1) to a ratio where Sb4 is added in three times molar access (i.e. at a 1:3 LRRC8A:Sb4 ratio, Fig. R1). This observation conforms with our previous study on the structure of the LRRC8A/Sb4 complex[2], which revealed the binding of three sybodies to the exposed epitope at the loose interface of the hexameric channel when added at the same substoichiometric ratio. In a dataset where Sb4 was added in a 1:1 ratio to LRRC8A subunits, a much higher conformational heterogeneity in the LRRD region was observed indicating binding of more than three sybodies (maximally six) and a disruption of the tight interface burying the epitope[2]. We expect a similar case here, where we have added Sb4 at a stoichiometric excess of three Sb4 molecules per LRRC8 subunit. The observed structure with a single LRRD resolved thus probably reflects the strongly increased structural heterogeneity of the LRRDs in a complex where statistically one of the A subunits might reside in a familiar conformation as found in the r position of homomeric LRRC8A/Sb4 complexes[2], while the other A subunits are more mobile as a consequence of the steric hindrance imposed by Sb4 binding.

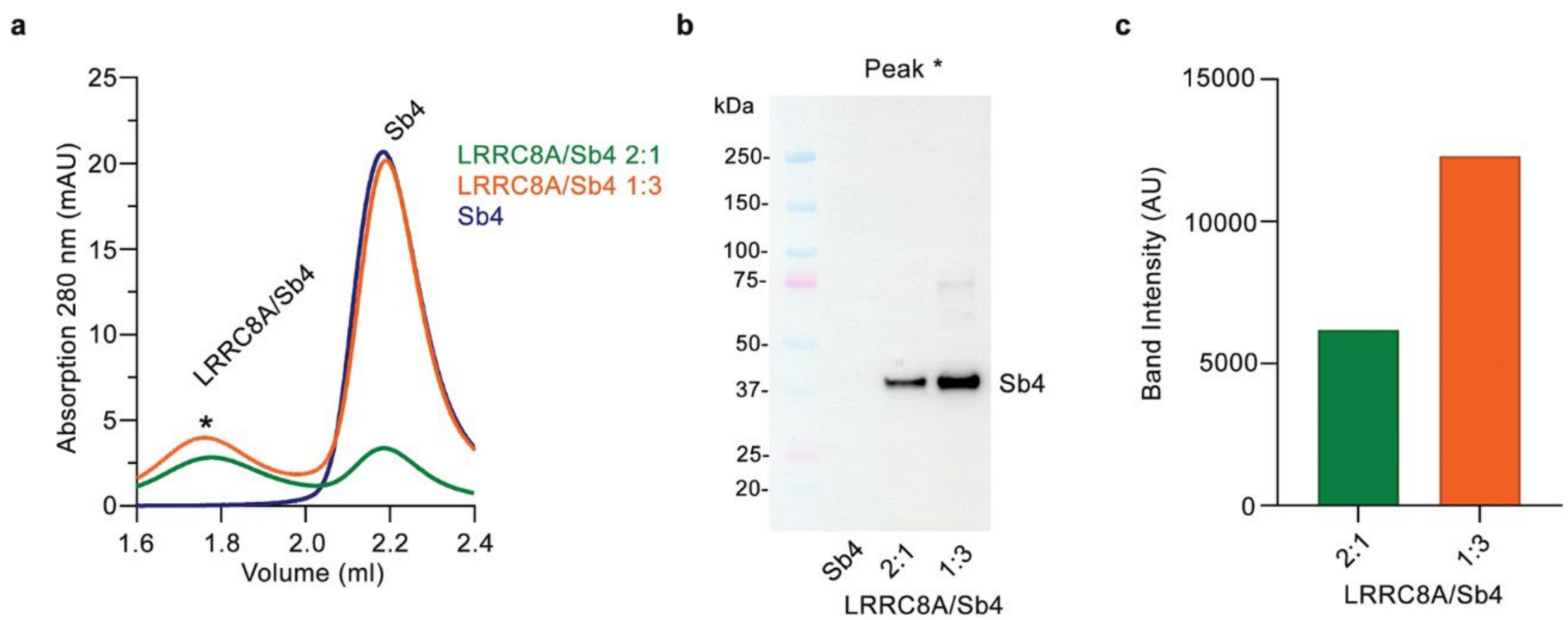

Fig. R1: Sb4 binding to LRRC8A at different stoichiometric ratios. **a**, Size exclusion chromatography profiles of the peak fraction containing LRRC8A (*) for indicated samples. **b**, Western blot of LRRC8A containing peak fractions shown in A (*), stained with an antibody recognizing the Myc-tag attached to Sb4. The difference in the intensity of the bands is apparent. **c**, Quantification of the band intensity from b illustrates the 2:1 ratio of bound sybody, suggesting that all six binding sites in the hexameric LRRC8A would be occupied in a stoichiometric excess of the binder.

3. Based on this and other structures of heteromeric VRACs, is it possible that the same type of subunits forms pairs?

In case of the LRRC8A/D/Sb1 complex it is obvious that the four A subunits arrange in two pairs and the two D subunits as single pair. As the sybody was added briefly before sample vitrification, we do not expect it to influence the subunit distribution and thus assume an equivalent organization in case of the LRRC8A/D/Sb4 complex, where the LRRDs of the A subunits binding Sb4 have now become more mobile and are consequently no longer resolved, except for one subunit. In this case, we do not know for certain whether the subunit with defined LRRD density always corresponds to the same relative position in the channel (designated as A1-A4) and we thus refrain from a definitive assignment of the other subunits in this structure.

We have explicitly stated this in our manuscript.

Line 343-346:

While the limited resolution of the PD and the disorder of most LRRDs has prevented a definitive assignment, we have arbitrarily allocated the A2 position to the subunit with observed Sb4 density and numbered the other subunits according to their position in the LRRC8A/D/Sb1[c1] complex (Figs. 2b, 5d).

4. The coloring scheme in the structures in Figs 2-7 is not explained clearly. Some A subunits are colored green and other purple without an explanation.

Throughout the manuscript we have used green and orange for alternating subunits of LRRC8A and pink and red for alternating LRRC8D subunits. We have now explained the coloring scheme in the legend of Fig. 3.

5. For Figs. 4&6, the heights of the top-view cross-sections should be indicated in a side-view figure.

We have marked the requested region in Fig. 5d (the former Fig. 6) and switched the order of panels a and b in Fig. 4 where we have indicated the displayed region panel a.

6. In Fig. 5a, a box is needed to indicate the insert.

The insert of Fig. 5a shows representative density at the interface between LRRC8A and Sb4, with interacting proteins displayed in unique colors to emphasize the high resolution defining the interaction. This resolution is isotropic across the entire structure. The exact region is in this case not relevant and would be hidden in the displayed orientation of the complex. The panel was now integrated in the figure showing the LRRC8A/D/Sb4 complex as Fig. 5a.

7. SI Table 1: um -> µm.

We have corrected this typo.

8. SI Tables 1&2: many numbers are shown in a German or French style, which needs to be changed.
We have corrected the supplementary tables.

Reviewer #4 (Remarks to the Author):

In this manuscript, Lehmann and colleagues investigate the structure and function of heteromeric LRRC8A/D volume-regulated anion channels using a combination of patch-clamp electrophysiology, cryo-EM, and X-ray crystallography. The authors present structures of the LRRC8D homomer, LRRC8A/D heteromers bound to the inhibitory sybody Sb1, the isolated LRRD of LRRC8A bound to the potentiating sybody Sb4, and the LRRC8A/D complex in the presence of Sb4. The technical quality of the structural data appears to be high (provided links to download and directly examine maps and models are not functional), and the work represents a substantial experimental effort.

While the dataset itself is strong, I have several fundamental concerns regarding the interpretation, novelty, and functional framing of the results. In particular, the manuscript would benefit from clearer motivation for how these structures advance our understanding of LRRC8A/D channel activation and permeation, improved clarity and consistency in the electrophysiological analysis, and a more cautious interpretation of structural observations in relation to channel "activation" and substrate selectivity.

We thank the reviewer for the generally constructive comments. With respect to the interpretation of the data, we want to emphasize that we aim to do this in a conservative manner. We have thus tried to better explain what we mean with activation and how this translates to the features in the observed structures. We also want to point out that we do not claim that our LRRC8A/D/Sb4 structure would necessarily represent a fully activated state.

As explained in the introduction of this document, the aim of the functional experiments in this study was to illustrate the strong effects of the two sybodies on the open probability of LRRC8A/D channels from humans and mouse on a qualitative level and we have thus used suitable conditions that better emphasize these effects. We do here not want to engage in a detailed investigation of LRRC8A/D substrate selectivity as this was already performed in previous studies[1,3] and since such investigations would go beyond the scope of this manuscript. We now also made it clearer in the text that our LRRC8A/D/Sb4 structure might not show a fully activated state and consequently changed the title to 'Structures of the volume-regulated anion channel LRRC8/D in activating and inhibiting conditions'.

1. Definition and evidence for channel "activation"

A central concern throughout the manuscript is the definition of activation. In Figure 1a, the authors state that LRRC8B/C/E−/− cells (endogenously expressing A/D) are activated by hypotonic

conditions. However, inspection of the current–time traces suggests that the slope under hypotonic conditions closely resembles that observed under isotonic conditions. This makes it difficult to conclude that hypotonic exposure induces a discrete activation event, as opposed to reflecting a slow time-dependent current increase that would also occur if isotonic conditions were simply extended.

We have now replaced Figure 1a, with new data showing the current response upon a change from isotonic to hypotonic conditions using the stronger activating Δmannitol system. In this case, the hypotonicity-induced currents are apparent. The low current density in this HEK293 derived cell-line is consistent with previous data[1]. The original data recorded in the Δsalt system showing the low currents upon hypotonic perfusion is still displayed in Fig. 1c, with the emphasis on the strong potentiation in response to the intracellular exposure to Sb4.

In contrast, overexpressed mouse LRRC8A/D channels (Fig. 1b) show a much more robust response to hypotonicity, followed by recovery upon re-exposure to isotonic buffer. This discrepancy makes it difficult to directly compare endogenous and overexpressed systems, and it raises the question of whether the summary data comparing isotonic and hypotonic currents in Fig. 1a accurately reflect activation rather than baseline drift.

As mentioned before, we do not want to directly compare human and mouse homologs under endogenous and overexpression condition but instead emphasize the effect of the sybodies, which selectively bind to both LRRC8A orthologs in an equivalent manner since the binding site is conserved[2]. To this end we have selected conditions that best illustrate the respective effect of sybody addition. We also showed that the stronger response of mouse homologs is related their larger expression levels at the plasma membrane (Supplementary Fig. 1c).

A related issue is evident in Supplementary Figure 1a, where human LRRC8A/D channels appear to produce currents close to the noise level. These observations further complicate the interpretation of activation across species and systems. The authors should extend isotonic recordings to the same time point used for hypotonic measurements and to compare these directly, to more convincingly demonstrate hypotonic activation.

The only purpose of this Figure panel was to show that the current response under equivalent conditions is smaller in cells transfected with the human orthologs compared to the mouse

orthologs. Part of this can be attributed to the five-fold lower expression levels on the surface (explained above and shown as Supplementary Fig. 1c), another part could be a generally weaker responsiveness of the human channel under the applied conditions. We have now recorded additional traces in which we have observed a somewhat more pronounced activation.

2. Clarity and consistency of electrophysiological conditions.

The electrophysiological experiments are difficult to interpret due to inconsistent experimental conditions and incomplete reporting. For example, Figure 1b (green trace) and Supplementary Figure 1a both appear to show mouse LRRC8A/D expressed in LRRC8−/− cells, yet the reported current densities differ substantially (~320 pA/pF versus ~120 pA/pF). Based on the manuscript, it is not clear why these datasets differ so markedly, particularly given the similar labeling and apparent experimental setup.

Please note that the two recordings show the response in two different buffer systems with the Δmanitol conditions showing stronger current response than the Δsalt conditions, which is consistent with the larger osmotic gradient of the former. Since both conditions contain similar outside chloride levels, this is likely an effect of the higher open probability of the channel reached in the former buffer as already observed in case of the endogenous currents in LRRC8$^{B,C,E-/-}$ cells (Fig. 1a, c). We have now specified the recording conditions in the title of the respective figure panels.

I strongly recommend that the authors include a summary table listing, for each electrophysiological dataset: cell line, species, expression method, bath and pipette solutions, osmotic protocol (Δsalt vs Δmannitol), and mode of sybody delivery. This would greatly improve transparency and reproducibility.

We have now provided such a summary table as new Supplementary Table 1. The reproducibility of the measurements is illustrated in the displayed repeats containing up to 17 independent recordings (in case of LRRC8A/D in presence of Sb4 shown in Fig. 1c). A statistical analysis is provided for all datasets.

In addition, the manuscript states that activation curves are derived from currents measured at "100 mV, 10 ms after the ramp." Given the complexity of the voltage protocol, a schematic

indicating where this measurement is taken would be extremely helpful. It would also be useful to explain why such an extensive voltage protocol is employed if only a small portion of it is used for quantifying activation, and whether other parts of the protocol reveal additional differences between conditions.

The mentioned protocol is now described in Supplementary Fig. 1a. This protocol allowed the continuous recording over a long period of time without clamping the voltage at positive potentials for too long affecting the cellular fitness. The obtained current-voltage relationships show similar properties during the activation process providing evidence that the increase in currents is due to the open probability increase of LRRC8A/D channels. The selected timepoints used to blot the current allows the evaluation of the current evolution at maximal point with pronounced outward rectification but before desensitization. The voltage protocol and selected traces are now shown in Supplementary Fig. 1a.

We have added the following sentence to the Methods, line 820-823:

The described protocol avoids the clamping at positive voltages for longer periods of time, allows the monitoring of the VRAC-characteristic outwardly rectifying current-voltage relationships during activation, and yields maximum currents at positive potential before desensitization.

3. Comparison of Sb1 and Sb4 effects

The functional effects of Sb1 and Sb4 are evaluated using markedly different experimental approaches. Sb1 is introduced via intracellular expression, whereas Sb4 is delivered through the patch pipette. In addition, different osmotic paradigms (Δmannitol versus Δsalt) are used across experiments. While each approach may be justified individually, the lack of symmetry makes direct comparison between inhibitory and potentiating effects difficult.

If it is not feasible to standardize these conditions, the manuscript would benefit from a clearer explanation of why different strategies were required and what limitations this imposes on the interpretation of the results. As presented, the variability in experimental design obscures the functional comparison between Sb1- and Sb4-mediated modulation.

As mentioned before, the protocols were optimized to clearly demonstrate the effects of the potentiating Sb4 and inhibiting Sb1 sybodies. For Sb4, we have chosen conditions under which in

its absence, the current response would be small to better emphasize its role as potentiator of activation. In this case, the application of the sybody via the patch pipette was required since its overexpression impaired cellular fitness. In contrast, we have used currents of overexpressed murine LRRC8A/D channels in Δmannitol conditions to obtain maximal response to better demonstrate the inhibitory effect by the expression of Sb1. As shown in our previous study, this protocol has enhanced the effect of applying Sb1 in the patch pipette[2]. We have now more explicitly explained this motivation in our manuscript and want to emphasize again that the comparison is qualitative and the conclusions are sound: Sb1 inhibits and Sb4 potentiates LRRC8A/D currents thus facilitating the interpretation of the structural data.

We have made the following changes to our manuscript, line 91-95:

'For our measurements, we have relied on two different buffer compositions termed Δsalt and Δmannitol, with the latter imposing a larger osmotic gradient leading to enhanced activating effects. The evoked currents were recorded by a consecutive series of ramp protocols that allowed the contiguous monitoring of current voltage relationships during the activation process (Supplementary Fig. 1a, Supplementary Table 1).'

Line 118-124:

'In our experiments with sybody Sb4, which was previously shown to enhance VRAC activity, endogenous currents were recorded in different HEK293 cell-lines in Δsalt conditions, where the response upon the change to hypotonic conditions was weak (Fig. 1c, Supplementary Fig. 1d). Since the overexpression of Sb4, as carried out for Sb1, affected the cellular fitness, the binder was applied via the pipette solution at a concentration of 1 µM and was allowed to equilibrate with the cytoplasm after establishment of the whole-cell configuration before application of hypotonic buffer. '

4. Biochemical characterization of purified complexes

The manuscript does not include biochemical data (e.g., SDS-PAGE gels or SEC profiles) demonstrating the purity and homogeneity of the LRRC8A/D complexes used for structural studies. This is particularly important given that LRRC8D may form homomeric assemblies (PMID: 32415200) and that only a one-step affinity purifications appear to have been used. The authors should add this data to assess whether LRRC8D homomers are present in the samples and how effectively heteromeric assemblies were isolated. Including representative purification data would significantly improve confidence in the reproducibility and interpretation of the structural results.

We have now provided a gel-filtration chromatogram and an SDS PAGE as Supplementary Fig. 5a for a representative LRRC8A/D preparation used for sample preparation of the LRRC8A/D/Sb1 complex. While A and D subunits are not separated by these procedures, the data shows the general quality of the sample.

In our study, we have relied on a single step purification via tagged D subunits, since initially used tandem purification protocols strongly decreased yields, which compromised data quality and consequently restricted the final map resolution. To quantify the relative ratio of both subunits, we have monitored their abundance during expression and purification and confirmed that a sample purified via the D subunit still contains a fraction of D homomers. To estimate the fraction of LRRC8D homomers, we have compared the fluorescence of differentially tagged A and D subunits between the cleared lysate and after affinity purification showing an enrichment of D by a factor of 3.5. Assuming the expression level of LRRC8D to be half of LRRC8A, as observed in the preparation of homomeric proteins, we estimate a ratio of LRRC8A/D : LRRC8D channels of 1 : 0.4. During data processing, we were able to efficiently separate LRRC8D homomers from LRRC8A/D channels due to their distinct conformational properties, which were defined in our structure of the LRRC8D homomer (Supplementary Fig. 3) and the absence of bound Sb1, which was well visible in 2D classes (Supplementary Fig. 5).

As described in our manuscript, we were also able to assign subunits based on distinct features of the extracellular subdomain in a high-resolution reconstruction, which further ensured the homogeneity of particles after 2D classification and 3D reconstruction. From 2D classes not showing any density of Sb1, a low-resolution reconstruction of LRRC8D homomers was obtained and showed general features of a LRRC8D homomer defined in the dataset of the homomeric channel at higher resolution (Supplementary Figures 3 and 5).

We have now better documented our classification strategy during processing in our revised manuscript in the novel Supplementary Fig. 5 and added the following sentences to the results, line 215-221:

'With detailed knowledge of the structural features of its constituents, we continued with investigations of LRRC8A/D channels that were overexpressed by co-transfection of LRRC8-/- cells with appropriate constructs and purified via an SBP tag attached to the less abundant LRRC8D subunit (Supplementary Fig. 5a), whose yield was about half of that of homomeric LRRC8A channels if expressed on its own. Based on this observation and the monitoring of the fluorescence ratio of the differentially tagged LRRC8 subunits during purification, we expect a ratio of D:A subunits of 1.75:1 in this preparation procedure.'

Line 224-229:

‘In this dataset, the presence of Sb1 allowed the unambiguous assignment of A subunits and it stabilized a non-conducting state of the protein. During data processing, particles of heteromeric LRRC8A/D containing bound Sb1 were readily identified during 2D classification and 3D reconstruction allowing their separation from particles not containing Sb1 density (Supplementary Fig 5b). The latter presumably represent LRRC8D homomers that constitute about 29% of all particles.’

5. Structural interpretation of pore selectivity and activation state

The authors attribute differences in pore-lining residues between A and D subunits to altered substrate selectivity and propose that these changes facilitate permeation of larger anions. However, these claims are based solely on static structural observations. Functional validation through mutagenesis (e.g., Phe-Arg substitutions, alanine scanning, or charge reversals) combined with permeability measurements are necessary to directly support these conclusions.

The structure of the pore lining at the extracellular ESD can be accurately determined based on the high-resolution data of the LRRC8A/D/Sb1 complex at a resolution of 3 Å and has allowed the unambiguous assignment of subunits. Towards the intracellular side, the detailed description is prevented by the unresolved conformation of the initial 12 residues at the N-termini of each subunit that project into the pore. In the ESD, the constriction is formed by an Arg residue of the A subunits which is replaced by a Phe in the D subunits. In a previous study, we have probed the role of this Arg and found that its replacement by a hydrophobic residue as found in C subunits decreases the selectivity of anions over cations and the residual permeability for the divalent anion sulfate[5]. We want to emphasize that the detailed investigation of substrate selectivity is beyond the scope of this work and we instead refer to previous studies describing the role of the D subunit[1,3].In the discussion, we also clarified that the current structural data is unable to explain all aspects of the distinct subunit selectivity between VRACs.

This is explained in the discussion in line 430-440:

‘Our structures of LRRC8A/D channels have also defined the organization of the constricting ESD acting as selectivity filter in VRACs. With two rigid Phe sidechains in D subunits replacing the long and flexible arginines found at the same position in LRRC8A, these residues have altered the shape of the pore constriction to make the permeation of large substrates such as glutamate plausible (Fig. 6b). While the observed pore geometry would be generally compatible with substrates up to the size of acidic amino acids, the dimensions of larger molecules that were also ascribed as substrates of certain VRACs such as ATP and the dinucleotide cGAMP would exceed

the observed pore diameter (Fig. 6b) and either require a strongly altered pore conformation or a larger oligomeric organization as found for homomeric LRRC8C channels. Whereas the former appears unlikely in light of the rigid organization of the ESD, there is currently no strong evidence for the latter.'

Related to this, the manuscript repeatedly refers to LRRC8A/D structures as representing an "activated" state. However, despite large rearrangements of the LRRDs—particularly in the presence of Sb4—the pore domain itself shows no detectable conformational changes. This suggests that the captured conformation may represent a primed or pre-activated state rather than an open, conducting channel. I would strongly caution against describing these structures as activated unless this distinction is clearly articulated.

We agree that the LRRC8A/D/Sb4 complex might not display a fully activated state of the channel and made this clearer in the manuscript. We also have changed the title of the manuscript to 'Structures of the volume regulated anion channel LRRC8A/D in activating and inhibiting conditions' and mentioned explicitly that the structure might represent an intermediate towards activation, line 418-420:

'We thus expect that the LRRC8A/D/Sb4 complex displays a potential intermediate towards activation rather than a fully activated conformation.'

Finally, while the Discussion proposes that Sb4-induced conformations could allow permeation of large anions, the electrophysiological experiments presented only examine chloride currents. No data are shown demonstrating enhanced permeation of larger substrates under Sb4 conditions. These claims should therefore be substantially tempered and additional functional data would be required to challenge this hypothesis

As detailed above, this part of the discussion refers to previous studies describing the role of the LRRC8D subunit in the permeation of amino acids osmolytes and anti-cancer drugs[1,3]. We have also emphasized that our current structural data does not fully explain such permeabilities for bulky substrates.

Reviewer #7 (Remarks to the Author):

This manuscript describes a study of the function and structure of heteromeric LRRC8A/D channels. The authors demonstrate that, as with homo-oligomeric LRRC8A channels, heteromeric A/D channels are activated by the previously described potentiating Sb4, which binds to the A subunit, and inhibited by Sb1. In order to investigate the mechanism underlying this inhibition and potentiation, the respective structures were determined. These structures reveal the 2:1 (A:D) subunit stoichiometry of the A/D heteromer and suggest that increased flexibility of the cytoplasmic LRRDs is the basis for channel activation. Unfortunately, no changes in the pore domain were observed between the activated and inhibited states, which leaves the mechanistic basis of the conducting state elusive.

The work supports the conclusions and claims. The methodology is sound and meets the standard in the field. The methods allow the work to be reproduced.

We thank the reviewer for the supportive comments.

I have the following comments:

** the authors determine the structure of homomeric LRRC8D. It is not immediately clear whether this assembly itself is an active form nor whether this specific assembly has any physiological relevance. Please elaborate beyond the rather technical motivation (line 123).*

It was previously shown that LRRC8 channels require the obligatory A subunits to localize at the plasma membrane[6]. Consequently, there exists currently no evidence that a homomeric LRRC8D channel would be of physiological relevance. In our study we have studied this assembly to better define the properties of the D subunits in a homomeric assembly and in the heteromeric LRRC8A/D channel.

We have added the following sentence, lines 147-149:

'While such homomeric LRRC8D channels do properly assemble, they would not localize at the plasma membrane and are thus presumably not relevant in a cellular setting.'

** line 212 > neighboring D subunits. Is this the correct term? One may argue that A1 and A2 are also neighbors to the respective D subunits, yet these are well resolved.*

At that part of the manuscript, we refer to the vicinity of D Subunits to A subunits in the A3 and A4 positions that plausibly disturb the conformation in these positions in the c1 conformation of the LRRC8A/D/Sb1 complex. Since the interactions on both sides of the D-subunit pair is not symmetric, they are not expected to exert the same influence on the neighboring LRRC8A

subunits in A1 and A2 positions. Please also note that we show a second conformation of the same complex (c2, Suppementary Fig. 6a) where the effect was stronger on the subunits in A1 and A2 positions.

** line 219 > A2 and A3, while A3 and A4 are meant*

We have corrected this mistake in our revised manuscript.

** line 250 > filter. The manuscript would benefit from detailing the difference in substrate specificity between the LRRC8A homomer and the A/D heteromer (that may be explained by the altered filter) in this section instead of line 332.*

As mentioned before, a detailed characterization of substrate selectivity is beyond the scope of this study and we instead refer to data published in previous studies, which have identified the D subunit as mandatory for the permeation of larger substrates such as amino acids, osmolytes and anti-cancer drugs[1,3]. In the indicated chapter, we interpret the structural features of the filter region that is well-defined in the LRRC8A/D/Sb1^{c1} structure mentioning that the replacement of an Arg to Phe might influence the permeation properties as described previously. In contrast, the reference to substrate selectivity in the later section describing the LRRC8A/D/Sb4 complex was intended to state that this structure does not fully explain the permeation of such large substrates.

We have introduced the following changes to the manuscript, line 280-286:

'This structure has provided a detailed view of the ESD harboring the most constricted part of the pore acting as selectivity filter. In the filter region, the two Phe 143 residues of the D subunits alter the chemical properties and expand the cross-section of the filter, which is otherwise defined by the long and flexible Arg sidechains located at equivalent positions on the A subunits. Presumably these features alter the properties of the filter to expand the substrate selectivity towards amino acids and osmolytes as described in previous studies16,18 (Fig. 4a, b, Supplementary Fig. 6d). '

** line 268 > the high-resolution structure of the LRRD of LRRC8A in complex with Sb4 does not provide conceptually new information and does has limited impact on the main message of the manuscript. Given that this manuscript is already very heavy on structure figures, I suggest to move this section and Fig. 5 to the supplement.*

In this case we do not agree with the reviewer. As a consequence of their higher mobility, the density of the LRRDs in LRRC8 channels is usually at an insufficient resolution to permit a detailed molecular interpretation. This is particularly the case for the interaction of LRRC8A with Sb4, where a previous structure homomeric LRRC8A channel[2] in complex with Sb4 has defined the approximate epitope but it did not reveal any details on the binding site and the respective interactions. The resolution of LRRD is even considerably worse in case of the LRRC8A/D/Sb4 complex described here, where the Sb4 has been added at a stoichiometric excess to maximize its occupancy, and where the interaction was only resolved in a single subunit. The previously unknown molecular details of the Sb4 binding have now been revealed in the X-ray structure of the LRRD of the A subunit in complex with Sb4 at an exceptionally high resolution (1.6 Å), which defines all interactions with high confidence.

In response to the comments of the reviewer we have merged the chapter describing the LRRD-Sb4 structure with the following chapter on the LRRC8A/D/Sb4 complex. We have also integrated panel a of the previous Fig. 5 in Fig. 6 (to create a new Fig. 5) and moved the remaining panels of this figure, describing details of the interaction, to a new Supplementary Fig. 7.

** LRR8CA/D/Sb4 structure. The authors appear to suggest that a previous LRR8CA/Sb4 structure did not reveal these dynamics in the LRRDs due to the substoichiometric amount of Sb4, whereas for the new structure a molar excess of Sb4 is added. However, the relative amount of Sb4 is only relevant if the respective epitope is accessible. In this respect, please explain the relevance of the lower salt concentration used to obtain the new structure. Are the Sb4 epitopes in all A subunits also accessible without decreasing the salt concentration?*

In our study, we have used the lower salt concentration in combination with the stoichiometric excess of Sb4, as both factors increase channel activity. We consequently think that the applied conditions alter the conformational heterogeneity of the LRRDs to make also previously buried interfaces accessible and consequently shift the equilibrium towards a fully occupied, activated state of LRRC8A/D. As detailed above in the response to comment 2 by Reviewer 2, we have estimated the occupancy of Sb4 to the LRRC8A channels and found evidence for interactions with all six A subunits when Sb4 is added in excess (Fig. R1). While, in principle, Sb4 should also shift the equilibrium towards the open state at resting conditions, our recordings in this and a previous study suggest that the effect is strongly enhanced upon application of activating conditions by either cell swelling (Fig. 1c) or decreasing the intracellular ionic strength[2].

** line 335 > LRRD mobility. The LRRD domains of LRRC8D are mobile in the Sb1-LRRC8A/D*

structure (Fig. 3) that represents an inhibited state. Please nuance the statement to reflect that activation appears to be correlated with mobility of the LRRD domain of A subunits specifically.

While we expect the A domains to play an important role in the regulation of pore opening, the correlation between mobility and activation has also been observed for other subunits. This includes a study of disease-causing mutations in LRRC8C, which increase domain mobility in this subunit[7] and observations that the presence of A subunits also destabilize the preferred orientation of interacting subunits in heteromeric channels. The destabilization of non-A subunits is even enhanced in case of the addition of Sb1, which rigidifies the LRRDs of A subunits in the observed conformation as observed previously for LRRC8A/C[8] and here for LRRC8A/D heteromers.

We have made the following changes, line 503-507:

'Still, the binding of Sb4 has led to a clear rearrangement of the tight interactions between A domains, and in light of its potentiating effect on channel activity, our data has reiterated the close relationship between LRRD mobility and channel activation that while apparent for LRRC8A also extends to other subunits as observed in previous studies'

** line 373 > membrane interaction. Comment on the surface properties of the respective LRRD and to what extent that would reconcile with an interaction with the membrane.*

Please note that the proposal was made based on the strong observed tilt of the LRRD found in this and an accompanying study[4], while there is currently no other evidence for a membrane interaction of the LRRD. A direct correlation with the surface properties would be ambiguous as the exact region of such potential interactions is still undefined. The chemical properties at the convex molecular surface of the LRRD facing the outside of the channel are diverse and contain a combination of ionic and hydrophilic residues but also aromatic and hydrophobic patches. Such hydrophobic patch would exist around Leu 510 at leucine rich repeat 4, which could get close to the region where the domain would interact with the membrane. However, in light of the absence of complementary data, we prefer at this stage not to engage in further speculations.

1 Lutter, D., Ullrich, F., Lueck, J. C., Kempa, S. & Jentsch, T. J. Selective transport of neurotransmitters and modulators by distinct volume-regulated LRRC8 anion channels. *J Cell Sci* **130**, 1122–1133 (2017). https://doi.org/10.1242/jcs.196253

2 Deneka, D. *et al.* Allosteric modulation of LRRC8 channels by targeting their cytoplasmic domains. *Nat Commun* **12**, 5435 (2021). https://doi.org/10.1038/s41467-021-25742-w

3 Planells-Cases, R. *et al.* Subunit composition of VRAC channels determines substrate specificity and cellular resistance to Pt-based anti-cancer drugs. *EMBO J* **34**, 2993–3008 (2015). https://doi.org/10.15252/embj.201592409
4 Bertelli, S., Wang, L., Klüssendorf, M., Gavazzo, P., Pusch, M., Dutzler, R., & Stauber, T. The intracellular subdomain of the volume regulated anion channel subunit LRRC8A is a hotspot of channel activation. *submitted*
5 Deneka, D., Sawicka, M., Lam, A. K. M., Paulino, C. & Dutzler, R. Structure of a volume-regulated anion channel of the LRRC8 family. *Nature* **558**, 254–259 (2018). https://doi.org/10.1038/s41586-018-0134-y
6 Voss, F. K. *et al.* Identification of LRRC8 heteromers as an essential component of the volume-regulated anion channel VRAC. *Science* **344**, 634–638 (2014). https://doi.org/10.1126/science.1252826
7 Quinodoz, M. *et al.* De novo variants in LRRC8C resulting in constitutive channel activation cause a human multisystem disorder. *EMBO J* **44**, 413–436 (2025). https://doi.org/10.1038/s44318-024-00322-y
8 Rutz, S., Deneka, D., Dittmann, A., Sawicka, M. & Dutzler, R. Structure of a volume-regulated heteromeric LRRC8A/C channel. *Nat Struct Mol Biol* **30**, 52–61 (2023). https://doi.org/10.1038/s41594-022-00899-0

REVIEWER COMMENTS

Reviewer #1 (Remarks to the Author):

The authors have added significant new data and analysis. I have no other comments except for the question raised about Fig. 6b. Because the whole paper is about A/D channel and A/D is specifically labelled in Fig. 6b, the figure in the current form does imply that A/D channel would conduct ATP. However, the evidence so far suggests that A/C and A/E channels, but not A/D, preferentially conduct larger nucleotides (at least for cGAMP). This is likely due to their different pore residues as discussed previously. I strongly encourage the authors to run their analysis with an experimentally validated A/D substrate such as Blasticidin to make their point validate. This is very important because in addition to the two possibilities the authors discussed, it is also possible that the pore residue sidechain dynamics underlies the (very low) permeability of metabolites as it has been recently suggested for the related large-pore pannexin channel (PMID: 41381453). Based on the new analysis, the authors may want to entertain this third possibility in which the sidechain movement may be enough to accommodate larger substrates than chloride.

In response to the comment of Reviewer 1, we have altered Fig. 6b to show Blasticidin instead of ATP.

We have also modified our discussion to include the mentioned manuscript of the pannexin channel.

Line 430-451:

Our structures of LRRC8A/D channels have also defined the organization of the ESD acting as selectivity filter of VRACs[23]. The rigid Phe sidechains in consecutive D subunits replacing the long and flexible arginine at the equivalent position of LRRC8A have altered the shape of the pore constriction (Fig. 4a, b). The consequent changes in the chemical properties of the filter combined with alternative conformations of the remaining arginine side-chains of A subunits could transiently expand the filter to permit the permeation of larger substrates such as glutamate[16,46] (Fig. 6b). A similar filter expansion by side chain rearrangements observed in a recent study of the heptameric pannexin PNX1 was proposed to underlie the permeation of ATP in this large-pore channel[47]. However, while such expanded pore geometry in LRRC8A/D channels would be generally compatible with substrates up to the size of acidic amino acids, the dimensions of larger molecules that were also ascribed as substrates of certain VRAC channels, such as blasticidin S[19], ATP[48,49] and the dinucleotide cGAMP[11,50] could even exceed the increased pore diameter resulting from side-chain rearrangements (Fig. 6b). These substrates might either require a strongly altered pore

conformation, resulting from conformational changes in the filter backbone, or a larger oligomeric organization as found in homomeric LRRC8C channels[21,28]. Whereas, in light of the rigid organization of the ESD (Figs. 4a and 5e), large conformational changes in the filter appear unlikely, there is currently also no evidence for a higher oligomeric organization in studies of heteromeric channels composed of two different subunits[21,22,43] (Figs. 3 and 5). Finally, the poor responsiveness and low current amplitudes of human LRRC8A/D heteromers points towards a possible organization of heteromeric VRACs as assemblies containing more than two different LRRC8 subunits, which was proposed in previous studies[16].
